# Indications for a genetic basis for big bacteria and description of the giant cable bacterium *Candidatus* Electrothrix gigas sp. nov.

**DOI:** 10.1128/spectrum.00538-23

**Published:** 2023-09-21

**Authors:** Jeanine S. Geelhoed, Casper A. Thorup, Jesper J. Bjerg, Lars Schreiber, Lars Peter Nielsen, Andreas Schramm, Filip J. R. Meysman, Ian P. G. Marshall

**Affiliations:** 1 Department of Biology, Research Group Geobiology, University of Antwerp, Wilrijk, Belgium; 2 Department of Biology, Center for Electromicrobiology, Aarhus University, Aarhus, Denmark; 3 Department of Biotechnology, Delft University of Technology, Delft, the Netherlands; University of Southern Denmark, Odense, Denmark

**Keywords:** cable bacteria, cell size, phylogenomics, actin, *Candidatus *Electrothrix gigas

## Abstract

Bacterial cells can vary greatly in size, from a few hundred nanometers to hundreds of micrometers in diameter. Filamentous cable bacteria also display substantial size differences, with filament diameters ranging from 0.4 to 8 µm. We analyzed the genomes of cable bacterium filaments from 11 coastal environments of which the resulting 23 new genomes represent 10 novel species-level clades of *Candidatus* Electrothrix and two clades that putatively represent novel genus-level diversity. Fluorescence *in situ* hybridization with a species-level probe showed that large-sized cable bacteria belong to a novel species with the proposed name *Ca*. Electrothrix gigas. Comparative genome analysis suggests genes that play a role in the construction or functioning of large cable bacteria cells: the genomes of *Ca*. Electrothrix gigas encode a novel actin-like protein as well as a species-specific gene cluster encoding four putative pilin proteins and a putative type II secretion platform protein, which are not present in other cable bacteria. The novel actin-like protein was also found in a number of other giant bacteria, suggesting there could be a genetic basis for large cell size. This actin-like protein (denoted big bacteria protein, Bbp) may have a function analogous to other actin proteins in cell structure or intracellular transport. We contend that Bbp may help overcome the challenges of diffusion limitation and/or morphological complexity presented by the large cells of *Ca*. Electrothrix gigas and other giant bacteria.

**IMPORTANCE:**

In this study, we substantially expand the known diversity of marine cable bacteria and describe cable bacteria with a large diameter as a novel species with the proposed name *Candidatus* Electrothrix gigas. In the genomes of this species, we identified a gene that encodes a novel actin-like protein [denoted big bacteria protein (Bbp)]. The *bbp* gene was also found in a number of other giant bacteria, predominantly affiliated to Desulfobacterota and Gammaproteobacteria, indicating that there may be a genetic basis for large cell size. Thus far, mostly structural adaptations of giant bacteria, vacuoles, and other inclusions or organelles have been observed, which are employed to overcome nutrient diffusion limitation in their environment. In analogy to other actin proteins, Bbp could fulfill a structural role in the cell or potentially facilitate intracellular transport.

## INTRODUCTION

Bacteria can vary greatly in size, with cell volumes differing over 10 orders of magnitude. Cell diameter ranges from only a few hundreds of nanometer for the free-living cells of *Candidatus* Pelagibacter communis up to 750 µm for the giant spherical cells of sulfur-oxidizing *Candidatus* Thiomargarita namibiensis (1, 2). Moreover, the recently discovered *Candidatus* Thiomargarita magnifica displays extremely long cells with an individual cell length reaching up to ~1 cm and a width of 50–110 μm (3). Like *Candidatus* Thiomargarita, many of the prokaryotes with extraordinarily large cell sizes are involved in sulfur metabolism (4). The giant sulfur bacteria are phylogenetically diverse (found in the Gammaproteobacteria, Campylobacterota, and Desulfobacterota) and display different life forms. The sulfur-oxidizing bacteria *Candidatus* Thiovulum and *Achromatium* grow as individual cells, sulfur-oxidizing *Beggiatoa*, *Thioploca, Thiothrix,* and sulfate-reducing *Desulfonema* grow as large filaments, and *Ca*. Thiomargarita may form loose cell chains, all displaying individual cell sizes up to tens of micrometers (5 – 10). Giant bacteria show large variations in morphologies and size; bacteria with cells that are larger on only one axis or form assemblages (e.g., filaments or cell clusters) have also been called “pseudo-giant” bacteria (11). For the purposes of this study, we define “giant” as encompassing both giant and pseudo-giant.

Giant bacteria show adaptations for large size. Many giant bacteria contain vacuoles or other inclusions that mitigate substrate limitation, both by shortening diffusion distances as the cytoplasmic content is pushed to the cell periphery and by serving as a storage place for nitrate, elemental sulfur, and organic compounds (4). High polyploidy has also been observed for giant cells, supporting high levels of gene expression and limited mRNA diffusion distance (12, 13). The extremely large cells of *Ca*. Thiomargarita magnifica contain membrane-bound organelles inside which genomic material and ribosomes are compartmentalized so that transcription and protein synthesis can take place in multiple places (3). However, a genetic basis for large cell size remains presently elusive.

Cable bacteria are sulfur-oxidizing multicellular filamentous bacteria, of up to several centimeters in length. The diameter of the cable bacterium filaments can vary substantially, with observed filament diameters ranging from 0.4 to 8 µm (14 – 16). Cable bacteria have been found worldwide in a range of marine environments, like tidal and subtidal sediments, salt marshes, and mangroves, as well as freshwater sediments and rice fields (17 – 21). No isolates of cable bacteria are presently available in pure culture, and, therefore, cable bacteria have been studied exclusively in the field or in laboratory enrichments of natural sediment.

Cable bacteria cells display a peculiar division of metabolic labor by spatially separating sulfide oxidation in deeper anoxic sediment and oxygen reduction at the sediment surface (22 – 24). These redox half-reactions are coupled by electron conduction through a network of parallel fibers that are embedded in the periplasm. These fibers bridge the cell division planes within a filament, thereby forming a continuous conductive structure that runs over the entire length of the bacterial filament (14, 23, 25 – 27). The external surface of the cable bacteria shows parallel, longitudinal ridges (23), and the conductive fibers are located within the ridges (14, 26). The size of the conductive fiber compartments is similar across cable bacteria of different sizes, and thick cable bacteria simply embed more ridge compartments compared to thin cable bacteria (14, 16). Detailed electrical investigations have shown that the fibers are highly conductive (27, 28). So-called cartwheel structures located in the cell division planes of the filament are also conductive and function as a fail-safe mechanism in case individual periplasmic fibers are damaged (25, 27).

Cable bacteria present an opportunity where we can study the genetic differences between differently sized cells of closely related bacteria. Our aim was to find out whether large-sized cable bacteria can be distinguished from smaller-sized cable bacteria based on genomic affiliation and whether large-sized cable bacteria possess specific genetic adaptations. To this end, we performed phylogenomic and comparative genomic analyses of cable bacterium filaments retrieved from multiple marine environments. We analyzed 40 genomes of cable bacteria filaments and found that all large-sized cable bacteria belong to a single novel species, with the proposed name *Candidatus* Electrothrix gigas. This species contains a gene encoding an actin-like protein, which is also found in a number of other giant bacteria.

## RESULTS AND DISCUSSION

### New cable bacteria genomes

**Fig 1:**
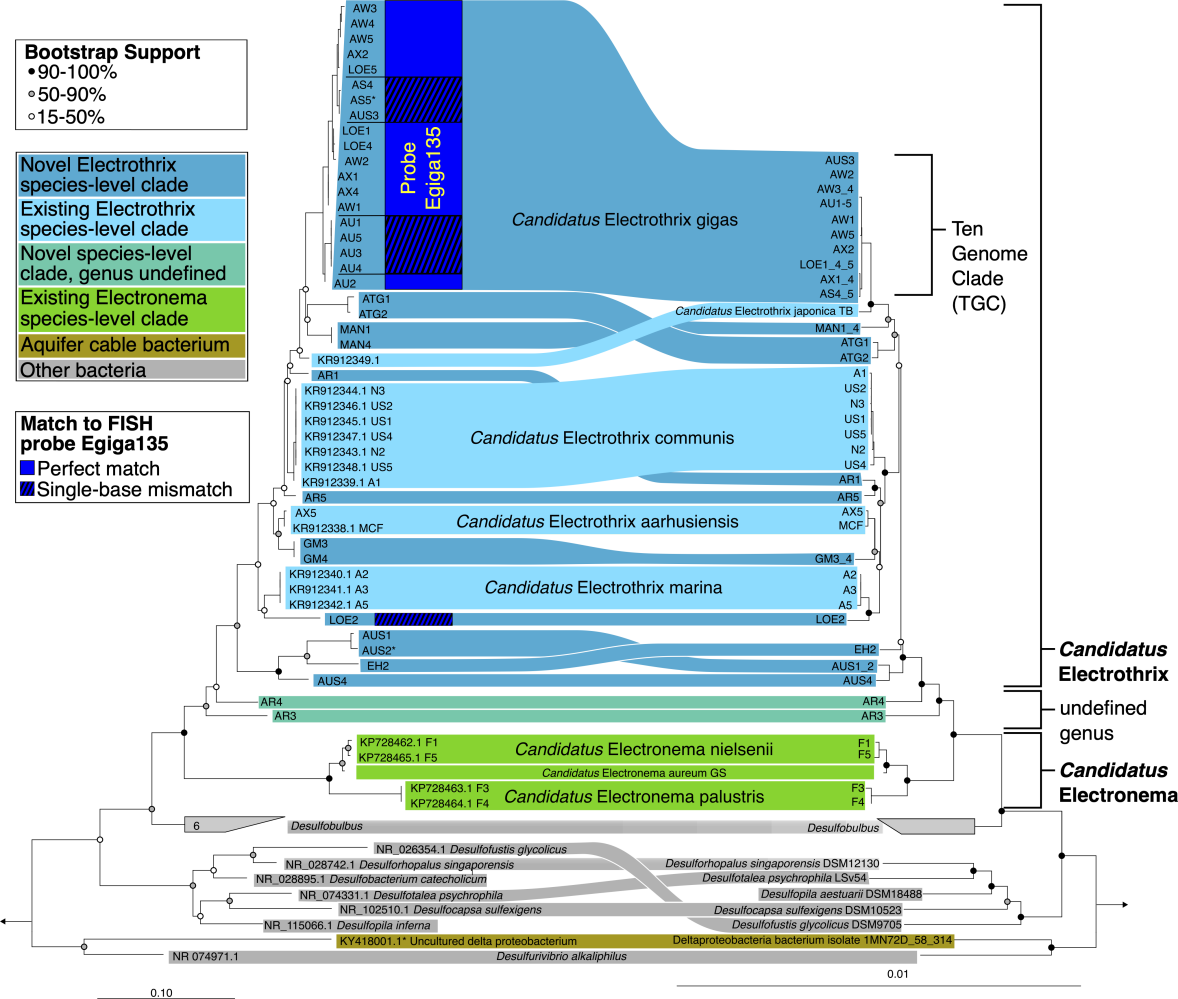
Phylogenetic diversity of cable bacteria based on (left-hand side) 16S rRNA genes extracted from sequenced genomes and (right-hand side) concatenated protein sequences aligned to the GTDB taxonomy. * indicates incomplete 16S rRNA sequences, AUS2 is 1,272 bp with the start missing, AS5 is 1,453 bp with the end missing, and KY418001.1 is a 559-bp-long fragment from 454 sequencing. Circles at nodes depict bootstrap support; scale bars show mean substitutions per site.

Individual filaments of cable bacteria of different sizes were retrieved from the sediments collected in 11 different coastal environments (Table 1). Whole-genome amplification of single filaments, followed by Illumina MiSeq sequencing and genome assembly were performed. Phylogenetic analysis of the complete 16S rRNA sequences extracted from these assemblies revealed that all filaments cluster within or directly adjacent to the genus *Candidatus* Electrothrix [Fig. 1 (16, 24)] in the Desulfobulbaceae family [Desulfobacterota phylum, previously known as Deltaproteobacteria (29)]. None of the filaments clustered within the genus *Candidatus* Electronema, which harbors cable bacteria from freshwater habitats (16, 20), or with the cable bacteria that are exclusive to groundwater aquifers (30, 31). Since all our filaments were retrieved from brackish and marine environments, our observations confirm that the monophyletic clade that includes the genus *Ca*. Electrothrix is confined to marine and brackish environments (16, 18).

**TABLE 1:** Origin and affiliation of cable bacteria filaments used in this study

Field site^*a*^	Environment	GPS coordinates	Affiliation of retrieved cable bacterium filaments	Reference
AUS: Yarra river, Melbourne	Estuary	37.833361 S 145.026528 E	*Ca*. Electrothrix gigas AUS3 *Ca*. Electrothrix sp. AUS1_2 *Ca*. Electrothrix sp. AUS4	This study
AUS: Western Port, Victoria	Mangrove	38.229 S 145.309 E	*Ca*. Electrothrix sp. MAN1_4	This study
B: Station 130, North Sea	Subtidal sediment	51.268833 N 2.903167 E	*Ca*. Electrothrix sp. AR1 *Ca*. Electrothrix sp. AR5 Desulfobulbaceae cable bacterium filament AR3 Desulfobulbaceae cable bacterium filament AR4	This study
DK: Løgten Strand	Intertidal sand	56.288184 N 10.381332 E	*Ca*. Electrothrix gigas LOE1_4_5 *Ca*. Electrothrix sp. LOE2	This study
DK: Aarhus Bay Harbour	Marine sediment	56.13889 N 10.21419 E	*Ca*. Electrothrix aarhusiensis MCF *Ca*. Electrothrix communis A1, N2, N3 *Ca*. Electrothrix marina A2, A3, A5	(16, 24)
J: Tokyo Bay, Tokyo	Marine sediment	35.5367 N 139.9167 E	*Ca*. Electrothrix japonica TB	(16)
NL: Oude Bietenhaven, Eastern Scheldt	Intertidal sand	51.447833 N 4.096500 E	*Ca*. Electrothrix gigas AS4_5	This study
NL: Rattekaai, Eastern Scheldt	Saltmarsh sediment	51.2621 N 4.1011 E	*Ca*. Electrothrix gigas AU1-5	This study
NL: Mokbaai, Texel	Saltmarsh sediment	53.005722 N 4.759472 E	*Ca*. Electrothrix gigas AW1, AW2, AW3_4, AW5	This study
NL: De Cocksdorp, Texel	Oyster reef	53.147889 N 4.901167 E	*Ca*. Electrothrix gigas AX1_4, AX2 *Ca*. Electrothrix aarhusiensis AX5	This study
NL: Lake Grevelingen	Marine lake	51.747 N 3.898 E	*Ca*. Electrothrix sp. ATG1_2	This study
USA: Elkhorn Slough, CA	Saltmarsh sediment	36.82 N 121.74 W	*Ca*. Electrothrix sp. EH2	This study
USA: Gulf of Mexico	Subtidal sediment	27.350533 N 90.562933 W	*Ca*. Electrothrix sp. GM3_4	This study
USA: Sippewissett Salt marsh, MA	Saltmarsh sediment	41.57595 N 70.63588 W	*Ca*. Electrothrix communis US1, US2, US4, US5	(16)
DK: Vennelystparken, Aarhus	Freshwater sediment	56.164874 N 10.207888 E	*Ca*. Electronema aureum GS	(24)
DK: Giber river, Eastern Jutland	River sediment	56.07476 N 10.22028 E	*Ca*. Electronema nielsenii F1, F5 *Ca*. Electronema palustre F3, F4	(16)

^*a*^ AUS: Australia, B: Belgium, DK: Denmark, J: Japan, NL: The Netherlands.

Assemblies that were both highly similar [whole-genome average nucleotide identity (ANI) >99.6%] and originated from filaments from the same field site were pooled for final genome assembly, resulting in 23 new draft genomes (Table S1). Additionally, we reconstructed the genomes of single cable bacterium filaments, for which the 16S rRNA gene and *dsrAB* phylogenies were studied previously (16) and also included six previously published cable bacteria genomes (24), thus providing 17 additional genomes for comparison. These 40 draft genome assemblies of cable bacteria vary greatly in size (0.6–4.4 Mbp) and estimated completeness (20.8–97.1%), but all clades are represented by at least one genome that is >63% complete. Good-quality data sets (nine with completeness >90% and six with completeness >80%; contamination <5%) are available for three previously published genomes (*Ca*. Electrothrix aarhusiensis MCF, *Ca*. Electrothrix communis US4, and *Ca*. Electronema aureum GS) and 12 of the new genomes (AU1-5, AW1, AX1_4, AW3, AW5, AS4_5, AW2, AX2, LOE1_4_5, GM3_4, AX5, and AR4; Table S1).

### Novel species-level and genus-level cable bacteria clades

The current taxonomic classification of cable bacteria is based on the 16S rRNA gene and *dsrAB* gene sequences (16). Phylogenomic analysis of our 40 draft genome assemblies enables us to move beyond single gene analysis and substantially expands the currently known diversity of cable bacteria (24). A whole-genome tree based on concatenated protein sequences of 120 marker genes shows a topology highly similar to the 16S rRNA gene phylogenetic tree (Fig. 1). Adopting a threshold of 95% for whole-genome ANI at the species level (32), only one of the new draft genomes (AX5) belonged to a previously described species (*Ca*. Electrothrix aarhusiensis), while 22 genomes were distributed among 10 new species-level clades (Table S2). Most of these new species clades are represented by one or two genomes, except for one species that is represented by 10 genomes. For this latter 10-genome species-level clade (TGC), we obtained genomes with 78.1–97.1% estimated genome completeness (2.2–4.6% contamination; Table S1), thus enabling a more detailed genomic analysis (as discussed below).

At some of the field sites, multiple (2–4) cable bacteria species were detected (Table 1), thus confirming previous observations that the microbial community within a given sediment can harbor a diversity of cable bacteria species (16, 18, 21, 33). Likewise, some species are present at multiples sites and show a global distribution (e.g., TGC was found in The Netherlands, Denmark, and Australia).

The genomes of filaments AR3 and AR4 are placed between the genera *Ca*. Electrothrix and *Ca*. Electronema in both the phylogenomic tree and the 16S rRNA gene tree. Both the values for 16S rRNA gene sequence identity and whole-genome average amino acid identity (AAI) are close to cutoffs proposed for delineation of a novel genus [94.5 and 65%, respectively (32, 34); Table S3], thus suggesting that AR3 and AR4 may constitute new candidate genera within the marine cable bacteria.

The AR3 and AR4 filaments were collected from sediment in the Belgian part of the North Sea (Station 130, subtidal cohesive sediment, water depth ~10 m), a site where cable bacteria are known to occur throughout the year (35, 36). Previously, 16S rRNA gene amplicon sequences affiliated to AR3 or AR4 were detected in sediments from the Baltic Sea (18) and also found to be associated to seagrass roots in sediments from Denmark, USA, and Australia (21). Overall, AR4 has been found within a salinity range of 20–34, and AR3 at salinities of 7–34. Accordingly, AR3 and AR4 appear to be saltwater-adapted cable bacteria found in the same salinity range and sediment environments as *Ca*. Electrothrix. Additional ecophysiological and genome analyses based on a larger number of strains and more complete genomes are required to see what specific physiological and metabolic differences can be found that separate AR3 and AR4 from *Ca*. Electrothrix.

### Identification of *Candidatus* Electrothrix gigas

From previous studies, it appeared that filament diameter could not be linked to taxonomic affiliation. The observed size differences in the diameter of cable bacteria can be very large [0.4–8 µm (14 – 16)] and considerable variation in the diameter of cable bacteria has been recorded within the same species and even within the same filament (16).

During our investigation of coastal sediments, we encountered thick filaments, with a diameter >2 µm, which exceed the typical diameter reported for the previously described *Ca*. Electrothrix species (15, 16, 33). Thick cable bacteria have earlier been documented for Rattekaai salt marsh [The Netherlands (14)] and Aarhus Bay marine sediments [Denmark (16)]. Upon closer inspection, we noted that in all the sediments harboring TGC genomes (Fig. 1), we were able to find thick filaments. This led to the hypothesis that these very large cable bacteria do not represent an extreme phenotypic variation but belong to a distinct species.

To verify that thick cable bacteria belong to the TGC-species level clade, we set up laboratory incubations with sediment from Rattekaai salt marsh, a site where the TGC has been recovered (Table 1), performed microscopy, and sequenced the 16S rRNA gene of thick filaments. We also applied fluorescence *in situ* hybridization (FISH) with a specific probe targeting the 16S rRNA of the TGC.

**Fig 2:**
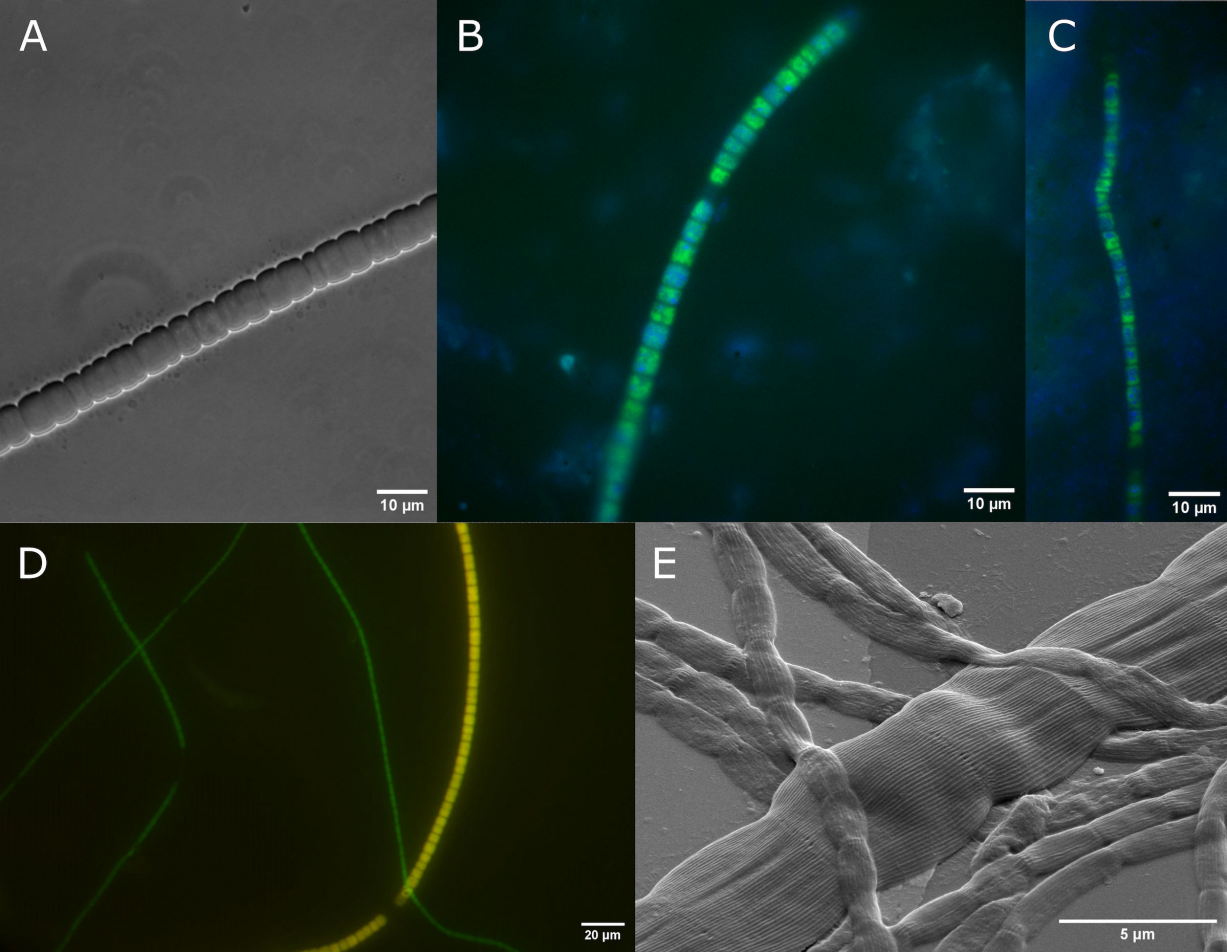
Images of cable bacteria, showing the large diameter of *Ca*. Electrothrix gigas. (**A**) Filament with a diameter of ~8 µm from incubated Rattekaai (NL) sediment. 16S rRNA gene sequences of retrieved filaments with similar diameter show that these belonged to *Ca*. Electrothrix gigas. (**B and C**) FISH with Rattekaai sediment shows *Ca*. E. gigas hybridized with Egiga134-Atto488 (green) and stained with 4’,6-diamidino-2-phenylindole (DAPI; blue). (**D**) FISH with sediment from Hou (DK) with probes DSB706-Atto488 (green) and Egiga134-Atto550 (red). *Ca*. Electrothrix gigas appears yellow from an overlay of red and green images, other cable bacteria appear green. (**E**) Scanning electron microscopy image showing continuous ridges characteristic for cable bacteria on the surface of thick and thin filaments from Hou sediment (DK). Scale bars: panels A–C, 10 µm; panel D, 20 µm; and panel E, 5 µm.

In the lab-incubated sediment from Rattekaai salt marsh, we found filaments with different diameters, including thick filaments that ranged up to 8 µm in diameter (Fig. 2A).

The 16S rRNA gene of three individual thick filaments was amplified using a nested PCR approach and sequenced. The resulting 16S rRNA gene sequences (fragment length 1,376 bp, *n* = 3 identical sequences) were 99.85% identical to the 16S rRNA gene sequence of genomes AX2 and AW5 of the TGC. A FISH probe (Egiga134) that targets the 16S rRNA of the TGC was developed based on the 16S rRNA sequences of all the cable bacteria. Comparison of the small subunit RNA database (SILVA RefNR v1.38), combined with an *in silico* analysis of hybridization conditions and a mismatch analysis, showed that the probe is highly specific (see Materials and Methods; Table S4). FISH with the incubated sediment from Rattekaai salt marsh (Fig. 2A) showed hybridization of thick filaments with a diameter ranging from 3.4 to 6.0 µm (*n* = 8; Fig. 2B) with probe Egiga134. In the second batch of incubated sediment, the diameter of Egiga134-hybridized filaments was 2.6–4.9 µm (*n* = 11; Fig. 2C). Non-hybridized filaments in these sediments ranged from 0.5 to 1.5 µm in diameter (*n* = 12). The length of the individual cells in a filament ranged from 2.6 to 5.5 µm for filaments that hybridized with the Egiga134 probe, and from 0.6 to 3.4 µm for non-hybridized filaments. Cell length within an individual filament varied by maximum 2.3 times.

Double hybridization with probes Egiga134 and DSB706 [targeting Desulfobulbaceae (37, 38)] was performed with intertidal sediment from Hou, Denmark. Thick filaments (5.0 ± 0.5 µm; *n* = 4) in the Hou sediment hybridized with both the Egiga134 and DSB706 probes. Thinner filaments (*d* = 1.6 ± 0.3 µm; *n* = 4) only hybridized to the DSB706 probe and not to the Egiga134 probe (Fig. 2D). Scanning electron microscopy of thick filaments from the same sediment revealed the characteristic ridges that run in parallel along the filament length, thus additionally confirming that these thick filaments are cable bacteria (14, 26) (Fig. 2E). The observed specific hybridization of probe Egiga134 to thick cable bacteria filaments shows that the TGC consists of large-sized cable bacteria, and we, therefore, propose the name *Ca*. Electrothrix gigas for this novel species.

In addition, a live preparation was made with tufts of filamentous bacteria washed out from this intertidal sediment, and thick cable bacteria (*d* = 6.1 ± 0.3 µm; *n* = 3) were observed together with Beggiatoaceae and thinner cable bacteria. These thick cable bacteria filaments contained apparent polyphosphate globules, as observed in the cable bacteria previously (24, 39, 40). The thick cable bacteria showed gliding motility, at a rate that was approximately twice as fast (~1.0 µm s^−1^ at 22°C; Video S1) as reported on average for cable bacteria (41).

### Genes unique to *Candidatus* Electrothrix gigas

To investigate a potential genetic basis for large cell size, we carried out a comparative analysis of cable bacteria genomes to identify genes that were unique to *Ca*. Electrothrix gigas (Fig. S1). Based on a common criterion for gene ortholog analysis [>70% DNA sequence identity and >80% similarity in size (42)], we identified 129 genes that were solely present in *Ca*. Electrothrix gigas. These genes were not all present in all *Ca*. Electrothrix gigas genomes, likely due to genome incompleteness. However, a phylogeny-based analysis of their distribution amongst all *Ca*. Electrothrix showed that these 129 genes were evenly spread within the *Ca*. Electrothrix gigas subtree (Fig. S2). Protein BLAST analysis against the other 30 cable bacteria genomes in the Desulfobulbaceae family (23 *Ca*. Electrothrix, 5 *Ca*. Electronema, and AR3 and AR4) showed that, for 98 of the encoded proteins, a protein with similar sequence (based on >40% amino acid sequence identity and >70% alignment length) was present in one or more cable bacteria genomes (Table S5). These proteins are less similar to the criteria set in the gene ortholog analysis but can be considered as functional homologs. Blastp analysis of the remaining 31 protein sequences against the non-redundant (nr) protein database, excluding cable bacteria, produced no significant hits for 17 of the proteins (e-value cutoff 0.05) indicating that they originate from a yet undescribed lineage or from an evolutionary innovation. Most of the 14 proteins for which similar sequences were found in the nr database contained either no known conserved domains or conserved domains that did not result in a clear functional annotation (Table S5).

Among the genes that were species specific for *Ca*. Electrothrix gigas, two patterns were conspicuous: (i) *Ca*. Electrothrix gigas contains a three-gene cluster that encodes an actin-like protein, a hypothetical protein, and a protein containing a putative actin-interacting Kelch domain, and (ii) *Ca*. Electrothrix gigas contains a cluster of five genes encoding four putative pilin proteins and a putative type II secretion platform protein (GspF).

### Identification of a novel bacterial actin-like protein in *Ca*. Electrothrix gigas

A gene encoding a protein belonging to a previously undiscovered clade of bacterial actin homologs was detected in all 10 *Ca*. Electrothrix gigas genomes and in one sibling genome in the same clade (*Ca*. Electrothrix MAN1_4; partial gene at the end of a contig). In *Ca*. Electrothrix gigas and *Ca*. Electrothrix MAN1_4, the gene coding for the novel actin-like protein was present in addition to the MreB actin homolog found in all rod-shaped bacteria, including cable bacteria.

Bacteria have evolved individual actin homologs for different functions in the cell. These include: MreB, a cell-shape protein present in rod-shaped bacteria; ParM and AlfA, which are involved in the segregation of plasmid DNA; FtsA, which plays a role in cell division; and MamK, which forms a filament to which magnetosomes are attached in magnetotactic bacteria (43 – 50). Actin proteins have ATPase activity and binding of ATP initiates polymerization into filaments, while hydrolysis to ADP results in conversion to the monomeric form of actin (47, 51, 52). The detected novel actin-like protein contains the five parts of the ATPase domain as well as conserved residues for nucleotide binding (53). However, it lacks the interaction site with the transmembrane protein RodZ (54), thus affirming that this protein is not an additional copy of MreB (Fig. S3). Structural prediction showed that the novel actin-like protein has a highly similar structure to MamK (98% of the residues modeled with 100% confidence) of *Magnetospirillum magneticum* AMB-1, which has been resolved to ~6.5 Å resolution (55) (Fig. S3).

**Fig 3:**
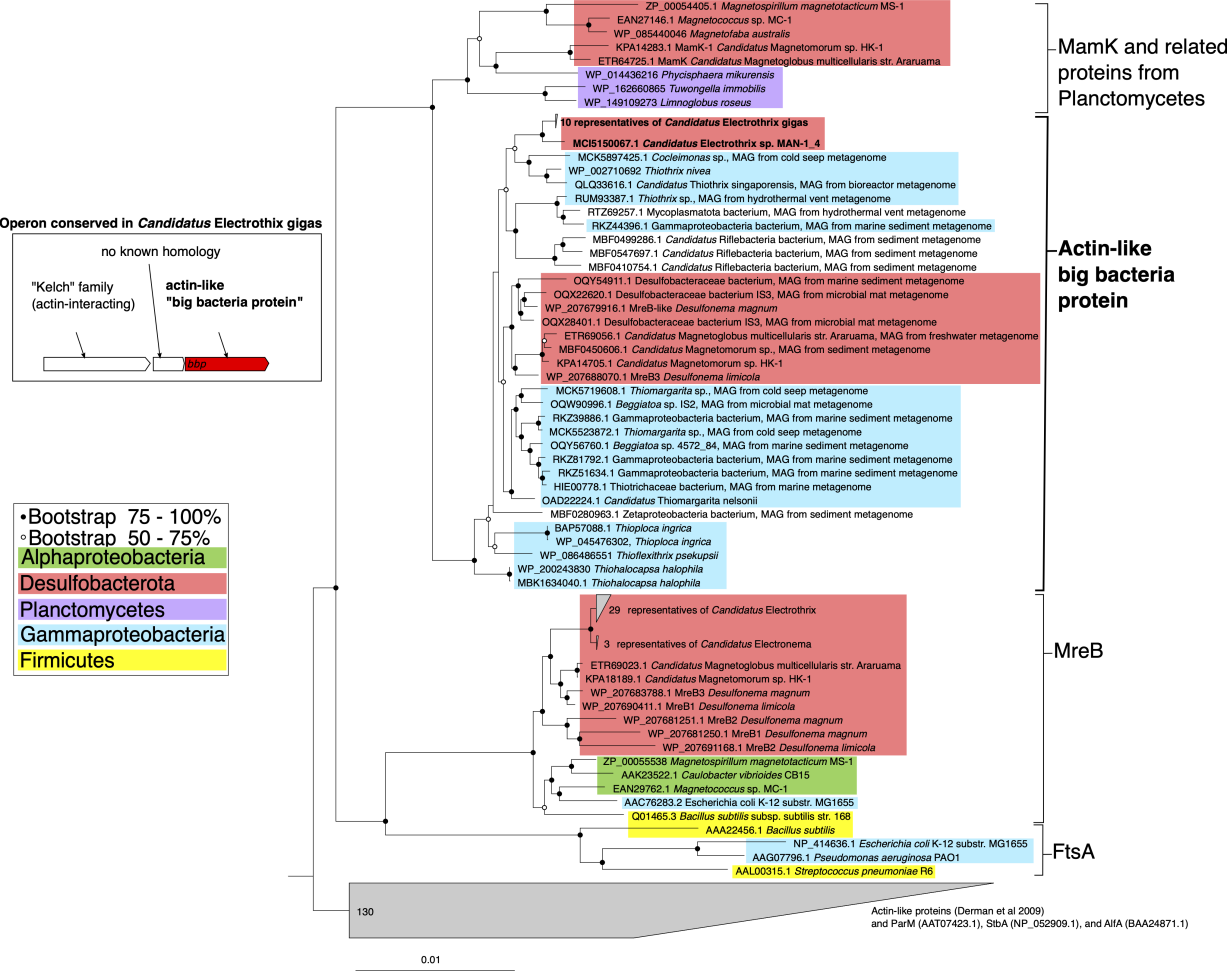
Phylogenetic tree based on actin-like proteins in bacteria, showing the placement of the novel big bacteria protein, Bbp, with an inset diagram showing the operon structure of *bbp* in *Candidatus* Electrothrix gigas. Circles at nodes depict bootstrap support; the scale bar shows mean substitutions per site.

Phylogenetic analysis of the 10 sequences obtained for the different genomes of *Ca*. Electrothrix gigas places the novel actin between MamK and MreB (Fig. 3). Strikingly, a comparison of the novel actin-like protein sequence of *Ca*. Electrothrix gigas with the protein database revealed a number of very similar proteins that are encoded in other large bacteria. We, therefore, named it “big bacteria protein” or Bbp. The bacteria that also encode Bbp occur either as very large cells [e.g., *Thiomargarita* (1, 56)], large cells in filaments [*Beggiatoa*, *Desulfonema*, *Thioploca, Thiothrix,* and *Thioflexithrix* (6, 7, 9, 10, 57 – 60)], or smaller cells in specialized morphologies [*Candidatus* Magnetomorum and *Candidatus* Magnetoglobus form cell clusters of ~5–10 µm diameter (61, 62)] (Fig. 3). Expression of the Bbp actin-like protein has been shown for *Desulfonema limicola* using proteomic analysis (59).

Not all bacteria that encode Bbp appear to be big. Spherical cells of *Thiohalocapsa halophila* encode Bbp but do not seem to be quite as large with diameters of 1.5–2.5 μm (63). A gene encoding Bbp was also detected in a metagenome-assembled genome (MAG) of *Cocleimonas* sp. The only *Cocleimonas* for which dimensions are known is *Cocleimonas flava,* which has rod-shaped cells of regular size [~0.35 µm wide and 1.7 µm long (64)]. The genes in MAGs of the Zetaproteobacteria and Riflebacteria that we identified as encoding Bbp were previously identified as encoding MamK (65). It is unclear whether Bbp is indeed acting as MamK in these bacteria, as magnetosomes have only been surmised based on genomic data. However, the clear absence of magnetosomes in *Ca*. Electrothrix gigas and the majority of other Bbp-containing organisms makes it clear that Bbp is not simply a novel kind of MamK. *Ca*. Magnetomorum and *Ca*. Magnetoglobus are magnetotactic bacteria, and they encode MamK in addition to Bbp, further suggesting that Bbp and MamK have distinct functions (Fig. 3).

The closest phylogenetic relatives of Bbp from *Ca*. Electrothrix gigas (order Desulfobulbales) are found in the Gammaproteobacteria, orders Thiotrichales and Beggiatoales, while sequences from other Desulfobacterota [order Desulfobacterales (e.g., *Desulfonema*, *Ca*. Magnetoglobus, and *Ca*. Magnetomorum)] are more distantly placed. Inference from the reconstructed phylogenetic tree suggests that *bbp* has been transferred horizontally between Gammaproteobacteria and Desulfobacterota several times. Many of these organisms indeed tend to live in close proximity [e.g., dense microbial mats consisting of *Beggiatoa* and *Desulfonema* spp. (9, 66)].

The presence of *bbp* in the genomes of *Ca*. Electrothrix gigas and other giant bacteria suggests a function of this actin-like protein that is related to large cell size. However, some species of large bacteria do not encode Bbp (based on blastp and tblastn options against available genomes in the NCBI database). Some of these are bacteria from other phyla like giant rod-shaped *Epulopiscium fishelsoni* (Firmicutes) and Cyanobacteria. *Ca*. Thiovulum is a large [up to ~50 µm across (5, 67, 68)] sulfur-oxidizing bacterium affiliated to the Campylobacteria (formerly Epsilonproteobacteria) but does not encode Bbp. Interestingly, *Achromatium*, a sulfur-oxidizing bacterium affiliated to the Gammaproteobacteria (order Chromatiales), is relatively closely affiliated to the Bbp-encoding sulfur-oxidizing bacteria, occurs in similar environments, both freshwater and marine (69), but does not seem to encode Bbp either. This suggests that Bbp is part of just one solution for the making or functioning of large cells or cell structures, and that other solutions likely also exist. Analysis of the available genomes for *Ca*. Thiomargarita magnifica did not reveal the presence of *bbp*. Although these genomes are incomplete, it seems unlikely that this gene would be missing in all five incomplete genomes. Perhaps the absence of *bbp* might be explained by cell shape: *Ca*. Thiomargarita magnifica has extremely long cells (~9,000 µm long and ~100 µm wide) in contrast to the very large spherical (up to ~750 µm diameter) cells of other *Ca*. Thiomargarita. While the cell diameter in *Ca*. T. magnifica is still larger than most bacteria at around 100 µm, it is clearly in a smaller size category than other *Ca*. Thiomargarita species, just as other *Ca*. Electrothrix that are smaller than *Ca*. Electrothrix gigas.

The second gene in the *Ca*. Electrothrix gigas-specific gene cluster encodes a hypothetical protein without putative conserved domains. The third gene encodes a protein similar to Kelch(-like) proteins. Eukaryotic Kelch(-like) proteins are involved in a variety of metabolic functions (70, 71), and some Kelch proteins are known to interact with actin (72 – 74). The protein in *Ca*. Electrothrix gigas has a predicted beta-propeller structure similar to Kelch proteins consisting of C-terminal tandem repeats [(73, 75, 76); Fig. S4]. The closest relatives of the *Ca*. Electrothrix gigas Kelch-like protein are found in different taxonomic classes and are proteins of varying size for which sequence homology exists only for the region containing the tandem repeats (~30–40% amino acid identity). Only two of the non-cable bacteria genomes that contain *bbp* also contain a gene encoding a protein with somewhat similar tandem repeats, *Thiothrix* sp. (RUM94246.1) and *Beggiatoa* sp. 4572_84 (OQY47631.1), but these two genes are located on other contigs than *bbp*. The Kelch-like protein is thus not likely to be necessary for the function of Bbp in big bacterial cells and cell clusters but may perform some role unique to *Ca*. Electrothrix gigas.

Currently, it is not clear where big cable bacteria emerge in the phylogenomic tree. The genome of *Ca*. Electrothrix sp. MAN1_4 contained a partial *bbp* gene (at the end of a contig, Fig. S5), the second gene of the *bbp-*cluster was present adjacent to *bbp*, but with opposite orientation, and a gene coding for the Kelch-like protein was not detected (estimated genome completeness ~77%). In a previous study, no thick cable bacteria were observed at the field site from where MAN1_4 cable bacteria filaments were retrieved [diameter 1.3 ± 0.5 µm (35)], but the observed cable bacteria filaments have also not been directly linked to the MAN1_4 species-level clade. *Candidatus* Electrothrix japonica is placed between *Ca*. Electrothrix gigas and *Ca*. Electrothrix MAN1_4 but *bbp* was not found in its genome (estimated genome completeness 73%). FISH analysis with a specific probe has shown that *Ca*. Electrothrix japonica forms thin filaments (16), consistent with the absence of *bbp*. Further investigations of cable bacteria morphology combined with more high-quality genomes are needed to study the emergence of *bbp* and the presence of the other two genes in the bbp-gene cluster.

At the moment, we can only speculate about the function of the novel actin-like protein that we have identified in the genomes of large cable bacteria and other large cells and cell clusters. We propose two possible functions. Bbp is predicted to be localized in the cytoplasm, where it may fulfill a structural function to support the large shape of bacteria or bacterial cell clusters. The presence of Bbp in MAGs of bacteria for which there is no information on cell morphology and size illustrates the need for high-quality genomes of cultures or single cells/filaments/cell assemblages with known cell dimensions. *Beggiatoa* spp. form filaments with different sizes and could, therefore, be a second model organism to study the presence of Bbp in relation to cell size. Another hypothesis is that Bbp could play a role in facilitating intracellular transport in large cells similar to actin filaments in eukaryotic cells (4, 77). Whatever the function of Bbp, not all large bacteria appear to require this function or fulfill it in the same way. Research into the expression and localization of Bbp actin filaments in cable bacteria and other giant bacteria [like recently studied for Lokiactin in an Asgard archaeon (78)] is necessary to shed light on the function of this novel actin.

### PilA genes in *Ca*. Electrothrix gigas

**Fig 4:**
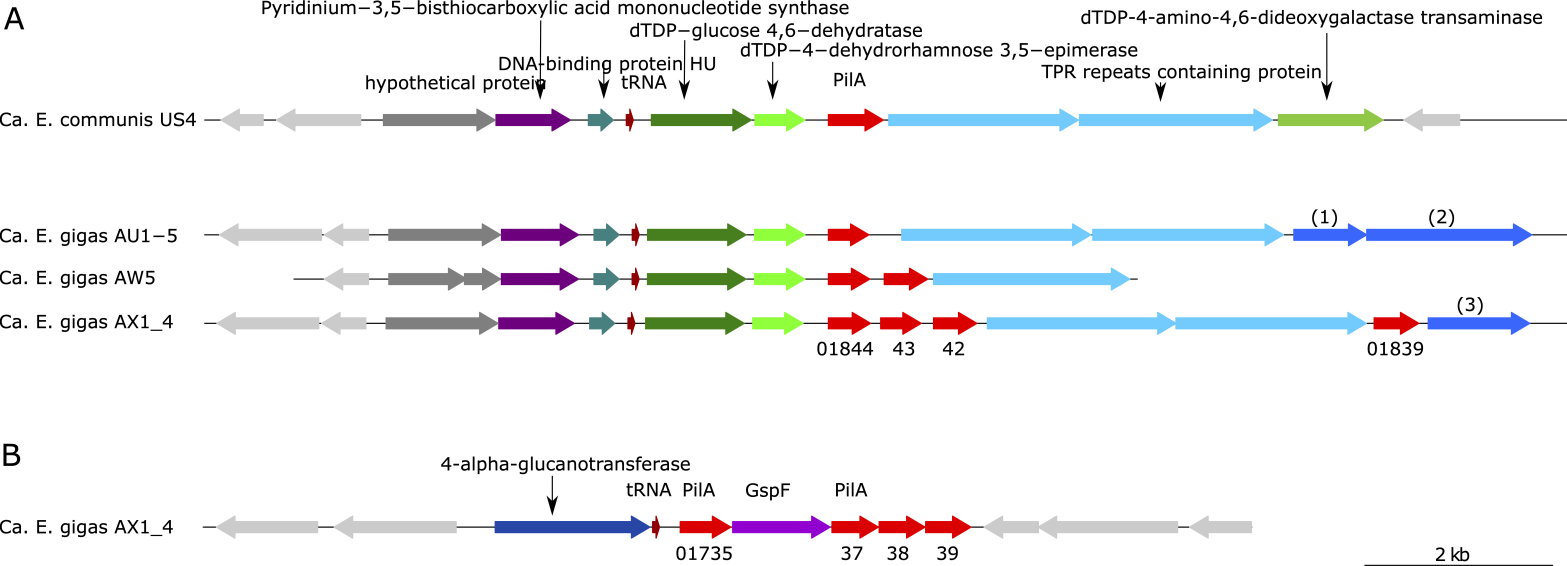
Putative PilA encoding gene clusters in *Ca*. Electrothrix genomes. (**A**) Gene cluster encoding two sugar-modifying enzymes, PilA, and proteins containing TPRs putatively involved in protein-protein interaction. *Ca*. Electrothrix gigas AW5 contains two *pilA* genes in this cluster, and AX1_4 and AW1 (not shown) contain four *pilA* genes of which one is located downstream of the two TPRs genes. (**B**) Putative operon of five genes specific to *Ca*. Electrothrix gigas (shown for AX1_4) consisting of four genes putatively encoding PilA homologs and one gene coding for the type II secretion platform protein GspF. Numbered blue arrows indicate genes with the following annotations: (1) methyltransferase, (2) phospholipid carrier-dependent glycosyltransferase, and (3) predicted glycosyltransferase. Numbering displays the locus tags of *pilA* genes in *Ca*. Electrothrix gigas AX1_4.

Genomes of the genera *Ca*. Electrothrix and *Ca*. Electronema contain a putative *pilA* gene that encodes the major component of the type IV pilus. The genomes of *Ca*. Electrothrix gigas, however, stand apart as they also contain a species-specific gene cluster with additional copies of *pilA*. This putative operon contains five genes encoding four putative pilin proteins and a putative type II secretion protein GspF (Fig. 4).

The type II secretion system consists of many components and is one of the systems involved in the export of proteins over the outer membrane to the extracellular environment. Many proteins of the type II secretion system and type IV pili machinery are homologous in structure and function. Cable bacteria encode the type IVa pili system, which is the most common in Desulfobacterota. GspF is the so-called platform protein and contains transmembrane helices and cytoplasmic domains. It interfaces with the pseudopilin, assembled from the major pseudopilin protein (GspG), in the cytoplasmic membrane. The major pseudopilin subunit GspG is analogous to PilA in the type IVa pili system (79 – 82).

Genomes of *Ca*. Electrothrix gigas encode three putative GspF proteins. Two of these appear to be present in all *Ca*. Electrothrix and *Ca*. Electronema genomes. Both these proteins are most closely related to GspF proteins of *Desulfobulbus* spp. (Fig. S6). The genomes of AR3 and AR4 (estimated genome completeness 80 and 70%, respectively) have at least one gene coding for GspF. The putative GspF protein that is encoded in the *Ca*. Electrothrix gigas specific gene cluster has an entirely different affiliation and is related to Gammaproteobacterial GspF sequences, albeit with relatively low identity (~30% aa sequence identity, ~50% positives) (Fig. S6).

The four genes that, together with *gspF*, are located in the *Ca*. Electrothrix gigas-specific gene cluster encode proteins that are affiliated with PilA and not with GspG (pseudopilin) (Fig. S7). The protein sequences are most closely related to those of Gammaproteobacteria, as was also observed for GspF encoded in this gene cluster, and indicate that these genes were acquired by horizontal gene transfer. It is striking that two of the four putative PilA proteins have amino acid sequences with the largest identity to proteins from members of giant Gammaproteobacteria, e.g., *Thioflexithrix*, *Achromatium*, *Thioploca*, *Thiomargarita,* and *Beggiatoa*.

The gene cluster containing *pilA* that has been detected in all *Ca*. Electrothrix species-level clades (except MAN1_4 and AUS4, estimated genome completeness 77 and 51%, respectively) comprises two genes encoding sugar-modifying enzymes and one or multiple gene(s) encoding protein(s) containing tetratricopeptide repeats (TPRs), which are putatively involved in protein-protein interaction. Interestingly, in three *Ca*. Electrothrix gigas genomes, there are multiple genes encoding PilA in this cluster. Genomes AX1_4 and AW1 have four, and AW5 has two *pilA* genes in the gene cluster (Fig. 4). The encoded PilA sequences cluster with those of other Desulfobacterota (Fig. S7).

PilA is highly expressed by cable bacteria [the most highly expressed protein detected in the proteome of *Ca*. Electronema aureum (24)]. However, it is not clear what the function of PilA in cable bacteria is. Previously, it has been hypothesized that PilA plays a role in conduction (24), based on analogy with *Geobacter* nanowires. However, recent research has revealed that conductive *Geobacter* nanowires are composed of stacked outer membrane cytochromes (83, 84), and that *Geobacter* PilA possibly is involved in secretion rather than conduction (85). Moreover, recent studies of the fiber sheath of cable bacteria suggest that the conductive fibers embed a novel nickel-sulfur cofactor that could be involved in electron conduction (86). So, PilA does not likely play a role in conduction in cable bacteria.

Functions that have been described for PilA are involvement in adhesion, virulence, motility, and secretion (85, 87 – 92). Future experiments to study the expression, structure, and subcellular localization of PilA are necessary to determine its function in cable bacteria. We assume that the additional *Ca*. Electrothrix gigas-specific gene cluster with proteins putatively involved in secretion and pilin formation may be somehow involved in the construction or functioning of *Ca*. Electrothrix gigas’ large cells. Gliding motility, as observed for cable bacteria, may involve pili and polysaccharide excretion (91, 92), so perhaps these proteins could play a role in the relatively fast motility, compared to cable bacteria in general, of *Ca*. Electrothrix gigas.

### Conclusion and perspectives

Previously, it was unclear whether the observed diversity in filament diameters of cable bacteria was a product of genomic diversity or phenotypic plasticity. In this study, we show that large cable bacteria belong to a novel species, *Candidatus* Electrothrix gigas. Moreover, the large morphology is likely linked to the presence of several genes that are present in *Ca*. Electrothrix gigas but absent in other *Ca*. Electrothrix species. These genes include a putative operon containing a gene, termed *bbp* (for big bacteria protein), encoding a newly recognized actin-like protein and a putative operon with additional copies of *pilA* and *gspF*. We hypothesize that Bbp plays a role in cell structure or intracellular transport to help overcome the challenges of diffusion limitation and/or morphological complexity presented by the large cells of *Ca*. Electrothrix gigas. The role of PilA in cable bacteria is currently unclear but one of the possibilities is that type IV pili may be involved in gliding motility. Further investigation of these proteins, such as their localization or heterologous expression, should shed further light on their precise role. We have found *bbp* in a phylogenetically and metabolically diverse set of other bacteria and available morphological data show that most of these have large cells or form multicellular structures. It may be that Bbp is important for many other large cells apart from *Ca*. Electrothrix gigas. Understanding the function of this protein will help reveal how large bacterial cells and cell structures are formed.

### Description of “*Candidatus* Electrothrix gigas” sp. nov.

gi’gas. Gr. masc. n. gigas, a giant (nominative in apposition), referring to the large filament diameter relative to other *Ca*. Electrothrix species. They are members of a clade within the candidate genus Electrothrix with cells of width greater than ~2.5 µm (commonly in the range of 2.5–8 µm), distinguishable by size, 16S rRNA gene sequence, and genome-based phylogeny. They can be detected by probe Egiga134 5′-CTATCCCGAGCATCTGGA-3′ combined with filamentous morphology.

## MATERIALS AND METHODS

### Sample collection and filament picking

Sediments were sampled at 11 different field sites in Belgium, Denmark, The Netherlands, USA, and Australia (Table 1). These sites represent different marine environments: intertidal sediments, salt marshes, mangrove, and subtidal sediments. Individual filaments of cable bacteria were either directly isolated from the natural sediment or after incubation of the sediment in the laboratory. For these laboratory incubations, sediment was sieved (<1.1 mm) and homogenized, left to settle overnight, and repacked into plexiglass core liners (diameter 40 mm) with the sediment surface level at the top of the core liner. The sediment cores were incubated at ~16°C in containers containing artificial seawater (ASW, Instant Ocean Sea Salt, salinity 30). The containers were kept in the dark to prevent photosynthesis and the overlying water was continuously aerated. Cable bacteria filaments were gently isolated from the surrounding sediment using custom-made glass hooks (23), washed repeatedly in filtered (0.22 µm) and autoclaved milliQ water, transferred to filtered (0.22 µm) and UV-sterilized TE buffer (10 mM Tris, 1 mM EDTA, pH 8) and kept at −20°C until further processing.

### Sequencing of the 16S rRNA gene of individual cable bacteria filaments

Individual cable bacteria filaments were isolated from incubated sediment, washed gently in sterile ASW, transferred into a PCR tube, and kept at −20°C. Filaments were subsequently lysed in 15 µL PCR water for 5 min at 95°C and all the components for PCR were prepared in a volume of 5 µL and added to the lysed filament. The final master mix was composed of buffer with MgCl_2_ (final concentration of 2 mM), dNTPs (0.2 mM each), forward primer (0.5 µM), reverse primer (0.5 µM), and Thermo Scientific DreamTaq polymerase (0.025 U/µL). General primers 27F (93) and 1492R (94) were used for amplification of the almost full-length 16S rRNA gene. Following initial denaturation for 2 min at 95°C, 30 cycles were performed with denaturation for 45 s at 95°C, annealing for 45 s at 46°C, and extension for 1 min 30 s at 72°C, followed by final extension for 10 min at 72°C. A second round of PCR was performed with the PCR product as template and nested primers that target Desulfobulbaceae, using forward primer DSBB280wF (95) and reverse primer DSBB + 1297R (96), as well as with the pairs 39Fc (5′-GGCTCAGAACGAACGCTG-3′)-DSBB + 1297R and DSBB280wF-1389R (5′-GGGCGGTGTGTACAAG-3′). Primer 39Fc is one base shorter, at the 5′ end, and lacks the degenerate bases compared to 39F (97). Primer 1389R is two bases shorter, at the 5′ end, compared to UNIV1389a (37). PCR conditions were as above, except for annealing at 50°C. PCR amplicon size and quantity were evaluated using gel electrophoresis. After cleanup with illustra ExoProStar (Cytiva), the amplicons were Sanger sequenced in both directions by Neuromics Support Facility at the University of Antwerp and the resulting sequences were merged.

### Whole-genome amplification and sequencing

Retrieved single filaments were lysed by ultrasonic bead-beating and the DNA was amplified using the GenomePlex Single Cell Whole Genome Amplification kit (Sigma-Aldrich) (16, 20). Paired-end sequencing libraries were prepared from amplified DNA using the Illumina TruSeq Nano DNA Library Prep kit with dual-indexed adapters. Sequencing was performed on a MiSeq Sequencer (Illumina) with 300 bp paired-end reads.

### Genome assembly

Illumina Miseq raw reads were clipped to remove adapters and quality-trimmed using Trimmomatic v0.36 [PE option, sliding window 6, average quality 20, minimum length 40 bp (98)]. Trimmed paired-end reads together with trimmed unpaired forward reads were assembled using SPAdes version 3.9.0 [using kmer lengths 21, 33, 55, 77, 99, and 127 and the option—careful (99)]. Contigs ≥1 kb were retained and binned based on tetranucleotide frequencies with MetaWatt v3.5.3 (100). Six out of 35 single filament (meta)genomic data sets required binning. The average nucleotide identity between single filament genomes was calculated with JSpecies v1.2.1. [option ANIm (101)]. Above 99.6% identity, single filament genomes were combined. For unbinned single filament genomes, all trimmed reads of the corresponding single filament data sets were used in the combined assembly. For binned single filament genomes, only trimmed reads that mapped to the binned single filament assembly (bbmap v34.25, minidentity 0.98; https://sourceforge.net/projects/bbmap/) were used for combined assembly with SPAdes v3.9.0 (same settings as above).

In addition to sequencing 35 new single cable bacterium filaments, we also reconstructed the genomes of single cable bacterium filaments previously studied for their 16S rRNA and *dsr*AB gene sequences (16). These filaments are *Ca*. Electrothrix communis N2, N3, US1, US2, US4, and US5, *Ca*. Electrothrix japonica TB, *Ca*. Electronema nielsenii F1, F5, and *Ca*. Electronema palustre F3, F4. Genome assembly was performed using the methods outlined for *Ca*. Electrothrix communis A1 and *Ca*. Electrothrix marina (24). Finally, we also included the published genomes of *Ca*. Electrothrix aarhusiensis MCF, *Ca*. Electrothrix marina A2, A3, and A5, *Ca*. Electrothrix communis A1 and *Ca*. Electronema aureum GS (24).

Genome completeness was estimated using CheckM v1.0.7 (102) using the reference data set for the order Desulfobacterales. Gene calling was performed using Prodigal v2.6.3 (103) implemented in Prokka v1.14.6 (104). Whole-genome average nucleotide identity (105) and whole-genome AAI [using both best hits (one-way AAI) and reciprocal best hits (two-way AAI)] were calculated with the online tool http://enve-omics.ce.gatech.edu/g-matrix/ (106).

### Comparative genome analysis

Orthologous genes were called using the ConSpeciFix pipeline (107) with a 0.7 nucleotide identity cutoff value for Usearch (108). Genes were filtered by coverage cutoff of 0.8 and clustered using MCL as integrated in ConSpeciFix with an inflation value of 1.6. This information, together with the phylogenomic tree, was used as input for the Count software tool (109) to determine propensity for gene loss (PGL) (Fig. S1). The PGL value indicates the likelihood of a given ortholog being a gene gain, loss, or neither. A PGL value of 0 means that the gene is spread evenly through the phylogenetic tree, suggesting no gene gain or gene loss event, while a PGL value approaching 1 means that the gene is not present in most of the genomes analyzed. We used a PGL threshold of 0.1, so that PGL <0.1 was considered an ortholog that has not been gained/lost in any *Ca*. Electrothrix lineage. This threshold was chosen as it represents a minimum in between the low-PGL and high-PGL genes. Using this method, genes gained in *Ca*. Electrothrix gigas were obtained.

For the gene gains in *Ca*. Electrothrix gigas, translated protein sequences were examined for the presence of a signal peptide using SignalP v 6.0 (110) and of transmembrane helices using TMHMM v 2.0 (111). Protein blast analysis was performed using local blast [ncbi-blast-2.10.1+ (112); against a database of translated protein sequences (generated using makeblastdb)] from all cable bacteria genomes except the 10 *Ca*. Electrothrix gigas genomes (threshold cutoff: e-value 10^-7^) and against the NCBI nr database (e-value 0.05). For selected protein sequences, conserved domains were annotated using CD-SEARCH (113) on the public NCBI BLAST server, with InterProScan (https://www.ebi.ac.uk/interpro/) and eggNOG-mapper v2.1 (114).

### Phylogenetic tree construction

16S rRNA gene trees were constructed for 16S rRNA gene sequences from 35 single filament genomes, together with already published 16S rRNA sequences of cable bacteria and other Desulfobulbaceae (16, 24). 16S rRNA gene sequences were extracted from the single filament genomes using RNAmmer v1.2 (115). Sequences were aligned using muscle (116) and a phylogenetic tree calculated using IQ-TREE v1.6.12 (117) with best-fit model GTR + F + I + G4 and 1,000 bootstrap calculations (option -b).

Whole-genome phylogenetic trees were constructed by first identifying 120 single-copy phylogenetic marker genes, aligning these genes, and concatenating the alignments using the “gtdbtk identify” and “gtdbtk align” workflows from the Genome Taxonomy Database toolkit version 0.3.2 [GTDB-Tk (118)]. A phylogenetic tree was then calculated based on this concatenated alignment using IQ-TREE version 1.6.12 (117) with best-fit model LG + F + R5 and 1,000 bootstrap calculations (option-bb).

Protein sequences for the phylogenetic tree of bacterial actin-like proteins were obtained using the publicly available NCBI BLAST server (112) against the nr database in June 2022 using the novel actin-like protein from *Ca*. Electrothrix gigas as query sequence. Database protein sequences down to a similarity value of 65% were included in the tree, along with MreB sequences identified by scanning all cable bacteria genomes using the MreB_Mbl model from Pfam version 14 (119) with hmmscan from HMMer v 3.1b1 (hmmer.org) and along with additional sequences for ParM, MreB, and MamK (46) and other actin-like proteins (120). Sequences were aligned using muscle v 3.8.31 (116) and then used to construct a tree with IQ-TREE v 1.6.5 (117) with best-fit model VT + R6 and 1,000 bootstraps (option -bb).

### Probe design, optimization, and FISH

Probe Egiga134 (5′-CTATCCCGAGCATCTGGA-3′), targeting the 16S rRNA sequences of the 10-genome species-level clade (TGC = *Ca*. Electrothrix gigas) at *E. coli* position 134–151, was designed and evaluated for its specificity based on all available cable bacterial 16S rRNA sequences using ARB (121). For *Ca*. Electrothrix gigas, the probe matched 12 out of 19 cable bacteria 16S rRNA gene sequences and two defined sequence groups within the species have a single mismatch: sequences AS4, AS5, AUS3 at position 3 and AU1, AU3, AU4, and AU5 at position 6 (Table S4). One additional cable bacterium sequence (*Ca*. Electrothrix LOE2) had a single central mismatch (position 9, A instead of C), while all other known cable bacteria sequences had at least two central mismatches. Comparison of the probe sequence to the small subunit rRNA SILVA database SSU138.1 RefNR using TestProbe 3.0 [https://www.arb-silva.de/search/testprobe/; >390,000 sequences in the database (122)] found no identical matches and identified only one sequence with one mismatch (same as for LOE2 above). There were another 40 hits with two mismatches (at central positions) which were primarily affiliated to the Desulfobulbaceae.

MathFISH (123) was applied for formamide curve simulation and mismatch analysis of probe Egiga134. *In silico* analysis at a hybridization temperature of 46°C predicted that 32% formamide in the hybridization buffer would correspond to the Td of the probe and should be sufficient to discriminate perfect match *Ca*. Electrothrix gigas sequences from the single mismatch non-target sequence LOE2, but also from two *Ca*. Electrothrix gigas-groups with a single mismatch at position 6 or 3. At more relaxed hybridization conditions (0–30% formamide), probe Egiga134 was predicted to target all members of *Ca*. Electrothrix gigas, while the cable bacterium LOE2 with one central mismatch and cable bacteria with two or more mismatches would not be targeted.

Sediment samples from Hou (intertidal sediment, Denmark, 55.9104 N 10.2467 E) and from Rattekaai (saltmarsh, The Netherlands, 51.2621 N 4.1011 E) were fixed in 50% ethanol. FISH with Atto488-, Atto550-, or Cy3-labelled probes was performed as previously described (124), including the use of positive (EUB338) and negative (NON338) control probes, and of probe DSB706 (at 35% formamide) to identify filamentous Desulfobulbaceae. The predicted hybridization conditions were first confirmed with sediment from Hou, mixed with a sediment enrichment culture containing *Ca*. Electrothrix communis. *Ca*. Electrothrix communis (diameter *d*, 0.8 ± 0.1 µm; *n* = 11) did not hybridize with probe Egiga134 at formamide concentrations from 10 to 35%, while thick filaments (*d*: ~4–6 µm) yielded bright signals. Both filament types were identified as cable bacteria by hybridization with probe DSB706. Together, this confirms the specificity of probe Egiga134 for *Ca*. Electrothrix gigas. Probe Egiga134 was subsequently used in double hybridization experiments with DSB706 at 35% formamide with sediment from Hou. FISH with probe Egiga134 was also performed with sediment from Rattekaai at 25% formamide. 16S rRNA gene sequences of thick filaments (up to ~8 µm) from the used laboratory incubated sediment showed a perfect match to probe Egiga134.

FISH was evaluated on a Nikon Eclipse Ni-U epifluorescence microscope equipped with an Andor Zyla camera, and images were processed using NIS Elements software (Nikon). Alternatively, FISH was evaluated using a Zeiss Axioplan 2 epifluorescence microscope with a Qimaging EXi Blue camera, and image processing performed with Image-Pro Insight and ImageJ software (125).

### Scanning electron microscopy

Cable bacteria were collected from Hou sediment, washed gently with water, and placed on a silicon wafer that was coated with a ~6 nm layer of platinum. After drying and sputtering with ~4 nm of platinum, the sample was imaged with Scanning Electron Microscope Versa 2D (Thermo Fisher Scientific) with ETD detector at 5 kV, 13 pA, and 10,000× magnification.

## Data Availability

All genomic data has been submitted to Genbank under Bioproject IDs PRJNA454675 and PRJNA278504. Prokka annotations of the 40 analyzed cable bacteria genomes are available at https://doi.org/10.5281/zenodo.7273704. A 16S rRNA gene sequence was deposited in Genbank under accession number OP646468.

## SUPPLEMENTAL MATERIAL

Supplemental Material spectrum.00538-23-s0001.pdf Tables S1–S5, Figures S1–S7 and Legend of Video S1.

Video S1 spectrum.00538-23-s0002.avi Gliding Ca. Electrothrix gigas. A live preparation was made of sediment from Hou, Denmark with gliding Ca. Electrothrix gigas. The movie was captured with an iPhone11 in real time and 29.97 frames per second through the eyepiece of a Leica BioMed light microscope, with 40× objective and phase contrast.

ASM does not own the copyrights to Supplemental Material that may be linked to, or accessed through, an article. The authors have granted ASM a non-exclusive, world-wide license to publish the Supplemental Material files. Please contact the corresponding author directly for reuse.

## References

[1] Schulz HN , Brinkhoff T , Ferdelman TG , Mariné MH , Teske A , Jorgensen BB . 1999. Dense populations of a giant sulfur bacterium in Namibian shelf sediments. Science 284:493–495. doi:10.1126/science.284.5413.493 10205058

[2] Zhao X , Schwartz CL , Pierson J , Giovannoni SJ , McIntosh JR , Nicastro D . 2017. “Three-dimensional structure of the Ultraoligotrophic marine bacterium "Candidatus Pelagibacter Ubique"” Appl Environ Microbiol 83:e02807-16. doi:10.1128/AEM.02807-16 27836840 PMC5244296

[3] Volland J-M , Gonzalez-Rizzo S , Gros O , Tyml T , Ivanova N , Schulz F , Goudeau D , Elisabeth NH , Nath N , Udwary D , Malmstrom RR , Guidi-Rontani C , Bolte-Kluge S , Davies KM , Jean MR , Mansot J-L , Mouncey NJ , Angert ER , Woyke T , Date SV . 2022. A centimeter-long bacterium with DNA contained in metabolically active, membrane-bound organelles. Science 376:1453–1458. doi:10.1126/science.abb3634 35737788

[4] Schulz HN , Jorgensen BB . 2001. Big bacteria. Annu Rev Microbiol 55:105–137. doi:10.1146/annurev.micro.55.1.105 11544351

[5] Hinze G . 1913. Beiträge zur Kenntnis der farblosen Schwefelbakterien. Ber deutsch bot Gesellsch 31:189–202. doi:10.1111/j.1438-8677.1913.tb05409.x

[6] Larkin JM , Shinabarger DL . 1983. Characterization of Thiothrix nivea. Int J Syst Evol Microbiol 33:841–846. doi:10.1099/00207713-33-4-841

[7] Nemoto F , Kojima H , Fukui M . 2011. Diversity of freshwater Thioploca species and their specific association with filamentous bacteria of the phylum Chloroflexi. Microb Ecol 62:753–764. doi:10.1007/s00248-011-9916-6 21800088

[8] Salman V , Yang T , Berben T , Klein F , Angert E , Teske A . 2015. Calcite-accumulating large sulfur bacteria of the genus Achromatium in Sippewissett salt marsh. ISME J 9:2503–2514. doi:10.1038/ismej.2015.62 25909974 PMC4611513

[9] Sharrar AM , Flood BE , Bailey JV , Jones DS , Biddanda BA , Ruberg SA , Marcus DN , Dick GJ . 2017. Novel large sulfur bacteria in the metagenomes of groundwater-fed chemosynthetic microbial mats in the Lake Huron basin. Front Microbiol 8:791. doi:10.3389/fmicb.2017.00791 28533768 PMC5421297

[10] Widdel F , Kohring G-W , Mayer F . 1983. Studies on dissimilatory sulfate-reducing bacteria that decompose fatty acids III. Characterization of the filamentous gliding Desulfonema limicola gen. nov. sp. nov., and Desulfonema magnum sp. nov. Arch Microbiol 134:286–294. doi:10.1007/BF00407804

[11] Ionescu D , Bizic M . 2019. Giant bacteria, p 1–10. In Encyclopedia of life sciences. John Wiley & Sons Ltd, Chichester, UK. doi:10.1002/047001590X

[12] Angert ER . 2021. Challenges faced by highly polyploid bacteria with limits on DNA inheritance. Genome Biol Evol 13:evab037. doi:10.1093/gbe/evab037 33677487 PMC8245194

[13] Ionescu D , Bizic-Ionescu M , De Maio N , Cypionka H , Grossart H-P . 2017. Community-like genome in single cells of the sulfur bacterium Achromatium oxaliferum. Nat Commun 8:455. doi:10.1038/s41467-017-00342-9 28878209 PMC5587575

[14] Cornelissen R , Bøggild A , Thiruvallur Eachambadi R , Koning RI , Kremer A , Hidalgo-Martinez S , Zetsche E-M , Damgaard LR , Bonné R , Drijkoningen J , Geelhoed JS , Boesen T , Boschker HTS , Valcke R , Nielsen LP , D’Haen J , Manca JV , Meysman FJR . 2018. The cell envelope structure of cable bacteria. Front Microbiol 9:3044. doi:10.3389/fmicb.2018.03044 30619135 PMC6307468

[15] Schauer R , Risgaard-Petersen N , Kjeldsen KU , Tataru Bjerg JJ , B Jørgensen B , Schramm A , Nielsen LP . 2014. Succession of cable bacteria and electric currents in marine sediment. ISME J 8:1314–1322. doi:10.1038/ismej.2013.239 24451206 PMC4030233

[16] Trojan D , Schreiber L , Bjerg JT , Bøggild A , Yang T , Kjeldsen KU , Schramm A . 2016. A taxonomic framework for cable bacteria and proposal of the candidate genera Electrothrix and Electronema. Syst Appl Microbiol 39:297–306. doi:10.1016/j.syapm.2016.05.006 27324572 PMC4958695

[17] Burdorf LDW , Tramper A , Seitaj D , Meire L , Hidalgo-Martinez S , Zetsche E-M , Boschker HTS , Meysman FJR . 2017. Long-distance electron transport occurs globally in marine sediments. Biogeosci 14:683–701. doi:10.5194/bg-14-683-2017

[18] Dam A-S , Marshall IPG , Risgaard-Petersen N , Burdorf LDW , Marzocchi U . 2021. Effect of salinity on cable bacteria species composition and diversity. Environ Microbiol 23:2605–2616. doi:10.1111/1462-2920.15484 33760391 PMC8252435

[19] Malkin SY , Rao AMF , Seitaj D , Vasquez-Cardenas D , Zetsche E-M , Hidalgo-Martinez S , Boschker HTS , Meysman FJR . 2014. Natural occurrence of microbial sulphur oxidation by long-range electron transport in the seafloor. ISME J 8:1843–1854. doi:10.1038/ismej.2014.41 24671086 PMC4139731

[20] Risgaard-Petersen N , Kristiansen M , Frederiksen RB , Dittmer AL , Bjerg JT , Trojan D , Schreiber L , Damgaard LR , Schramm A , Nielsen LP . 2015. Cable bacteria in freshwater sediments. Appl Environ Microbiol 81:6003–6011. doi:10.1128/AEM.01064-15 26116678 PMC4551263

[21] Scholz VV , Martin BC , Meyer R , Schramm A , Fraser MW , Nielsen LP , Kendrick GA , Risgaard-Petersen N , Burdorf LDW , Marshall IPG . 2021. Cable bacteria at oxygen-releasing roots of aquatic plants: a widespread and diverse plant-microbe association. New Phytol 232:2138–2151. doi:10.1111/nph.17415 33891715 PMC8596878

[22] Geerlings NMJ , Karman C , Trashin S , As KS , Kienhuis MVM , Hidalgo-Martinez S , Vasquez-Cardenas D , Boschker HTS , De Wael K , Middelburg JJ , Polerecky L , Meysman FJR . 2020. Division of labor and growth during electrical cooperation in multicellular cable bacteria. Proc Natl Acad Sci U S A 117:5478–5485. doi:10.1073/pnas.1916244117 32094191 PMC7071850

[23] Pfeffer C , Larsen S , Song J , Dong M , Besenbacher F , Meyer RL , Kjeldsen KU , Schreiber L , Gorby YA , El-Naggar MY , Leung KM , Schramm A , Risgaard-Petersen N , Nielsen LP . 2012. Filamentous bacteria transport electrons over centimetre distances. Nature 491:218–221. doi:10.1038/nature11586 23103872

[24] Kjeldsen KU , Schreiber L , Thorup CA , Boesen T , Bjerg JT , Yang T , Dueholm MS , Larsen S , Risgaard-Petersen N , Nierychlo M , Schmid M , Bøggild A , van de Vossenberg J , Geelhoed JS , Meysman FJR , Wagner M , Nielsen PH , Nielsen LP , Schramm A . 2019. On the evolution and physiology of cable bacteria. Proc Natl Acad Sci U S A 116:19116–19125. doi:10.1073/pnas.1903514116 31427514 PMC6754541

[25] Thiruvallur Eachambadi R , Bonné R , Cornelissen R , Hidalgo-Martinez S , Vangronsveld J , Meysman FJR , Valcke R , Cleuren B , Manca JV . 2020. An ordered and fail-safe electrical network in cable bacteria. Adv Biosyst 4:e2000006. doi:10.1002/adbi.202000006 32449305

[26] Jiang Z , Zhang S , Klausen LH , Song J , Li Q , Wang Z , Stokke BT , Huang Y , Besenbacher F , Nielsen LP , Dong M . 2018. In vitro single-cell dissection revealing the interior structure of cable bacteria. Proc Natl Acad Sci U S A 115:8517–8522. doi:10.1073/pnas.1807562115 30082405 PMC6112711

[27] Meysman FJR , Cornelissen R , Trashin S , Bonné R , Martinez SH , van der Veen J , Blom CJ , Karman C , Hou J-L , Eachambadi RT , Geelhoed JS , Wael KD , Beaumont HJE , Cleuren B , Valcke R , van der Zant HSJ , Boschker HTS , Manca JV . 2019. A highly conductive fibre network enables centimetre-scale electron transport in multicellular cable bacteria. Nat Commun 10:4120. doi:10.1038/s41467-019-12115-7 31511526 PMC6739318

[28] Bonné R , Hou J-L , Hustings J , Wouters K , Meert M , Hidalgo-Martinez S , Cornelissen R , Morini F , Thijs S , Vangronsveld J , Valcke R , Cleuren B , Meysman FJR , Manca JV . 2020. Intrinsic electrical properties of cable bacteria reveal an Arrhenius temperature dependence. Sci Rep 10:19798. doi:10.1038/s41598-020-76671-5 33188289 PMC7666173

[29] Parks DH , Chuvochina M , Rinke C , Mussig AJ , Chaumeil P-A , Hugenholtz P . 2022. GTDB: an ongoing census of bacterial and archaeal diversity through a phylogenetically consistent, rank normalized and complete genome-based taxonomy. Nucleic Acids Res 50:D785–D794. doi:10.1093/nar/gkab776 34520557 PMC8728215

[30] Müller H , Bosch J , Griebler C , Damgaard LR , Nielsen LP , Lueders T , Meckenstock RU . 2016. Long-distance electron transfer by cable bacteria in aquifer sediments. ISME J 10:2010–2019. doi:10.1038/ismej.2015.250 27058505 PMC4939269

[31] Müller H , Marozava S , Probst AJ , Meckenstock RU . 2020. Groundwater cable bacteria conserve energy by sulfur disproportionation. ISME J 14:623–634. doi:10.1038/s41396-019-0554-1 31728021 PMC6976610

[32] Konstantinidis KT , Rosselló-Móra R , Amann R . 2017. Uncultivated microbes in need of their own taxonomy. ISME J 11:2399–2406. doi:10.1038/ismej.2017.113 28731467 PMC5649169

[33] Marzocchi U , Bonaglia S , van de Velde S , Hall POJ , Schramm A , Risgaard-Petersen N , Meysman FJR . 2018. Transient bottom water oxygenation creates a niche for cable bacteria in long-term anoxic sediments of the Eastern Gotland Basin. Environ Microbiol 20:3031–3041. doi:10.1111/1462-2920.14349 29971901

[34] Yarza P , Yilmaz P , Pruesse E , Glöckner FO , Ludwig W , Schleifer K-H , Whitman WB , Euzéby J , Amann R , Rosselló-Móra R . 2014. Uniting the classification of cultured and uncultured bacteria and archaea using 16S rRNA gene sequences. Nat Rev Microbiol 12:635–645. doi:10.1038/nrmicro3330 25118885

[35] van de Velde S , Lesven L , Burdorf LDW , Hidalgo-Martinez S , Geelhoed JS , Van Rijswijk P , Gao Y , Meysman FJR . 2016. The impact of electrogenic sulfur oxidation on the biogeochemistry of coastal sediments: a field study. Geochim Cosmochim Acta 194:211–232. doi:10.1016/j.gca.2016.08.038

[36] van de Velde S , Van Lancker V , Hidalgo-Martinez S , Berelson WM , Meysman FJR . 2018. Anthropogenic disturbance keeps the coastal seafloor biogeochemistry in a transient state. Sci Rep 8:5582. doi:10.1038/s41598-018-23925-y 29615805 PMC5883055

[37] Loy A , Lehner A , Lee N , Adamczyk J , Meier H , Ernst J , Schleifer K-H , Wagner M . 2002. Oligonucleotide microarray for 16S rRNA gene-based detection of all recognized lineages of sulfate-reducing prokaryotes in the environment. Appl Environ Microbiol 68:5064–5081. doi:10.1128/AEM.68.10.5064-5081.2002 12324358 PMC126405

[38] Lücker S , Steger D , Kjeldsen KU , MacGregor BJ , Wagner M , Loy A . 2007. Improved 16S rRNA-targeted probe set for analysis of sulfate-reducing bacteria by fluorescence in situ hybridization. J Microbiol Methods 69:523–528. doi:10.1016/j.mimet.2007.02.009 17408790

[39] Geerlings NMJ , Zetsche EM , Hidalgo-Martinez S , Middelburg JJ , Meysman FJR . 2019. Mineral formation induced by cable bacteria performing long-distance electron transport in marine sediments. Biogeosci 16:811–829. doi:10.5194/bg-16-811-2019

[40] Sulu-Gambari F , Seitaj D , Meysman FJR , Schauer R , Polerecky L , Slomp CP . 2016. Cable bacteria control iron-phosphorus dynamics in sediments of a coastal hypoxic basin. Environ Sci Technol 50:1227–1233. doi:10.1021/acs.est.5b04369 26720721

[41] Bjerg JT , Damgaard LR , Holm SA , Schramm A , Nielsen LP . 2016. Motility of electric cable bacteria. Appl Environ Microbiol 82:3816–3821. doi:10.1128/AEM.01038-16 27084019 PMC4907201

[42] Bobay L-M , Ochman H . 2018. Factors driving effective population size and pan-genome evolution in bacteria. BMC Evol Biol 18:153. doi:10.1186/s12862-018-1272-4 30314447 PMC6186134

[43] Fink G , Szewczak-Harris A , Löwe J . 2016. SnapShot: the bacterial cytoskeleton. Cell 166:522–522. doi:10.1016/j.cell.2016.06.057 27419875

[44] Gerdes K , Howard M , Szardenings F . 2010. Pushing and pulling in prokaryotic DNA segregation. Cell 141:927–942. doi:10.1016/j.cell.2010.05.033 20550930

[45] Jones LJ , Carballido-López R , Errington J . 2001. Control of cell shape in bacteria: helical, actin-like filaments in Bacillus subtilis. Cell 104:913–922. doi:10.1016/s0092-8674(01)00287-2 11290328

[46] Komeili A , Li Z , Newman DK , Jensen GJ . 2006. Magnetosomes are cell membrane invaginations organized by the actin-like protein MamK. Science 311:242–245. doi:10.1126/science.1123231 16373532

[47] Møller-Jensen J , Jensen RB , Löwe J , Gerdes K . 2002. Prokaryotic DNA segregation by an actin-like filament. EMBO J 21:3119–3127. doi:10.1093/emboj/cdf320 12065424 PMC126073

[48] Møller-Jensen J , Löwe J . 2005. Increasing complexity of the bacterial cytoskeleton. Curr Opin Cell Biol 17:75–81. doi:10.1016/j.ceb.2004.11.002 15661522

[49] Polka JK , Kollman JM , Mullins RD . 2014. Accessory factors promote AlfA-dependent plasmid segregation by regulating filament nucleation, disassembly, and bundling. Proc Natl Acad Sci U S A 111:2176–2181. doi:10.1073/pnas.1304127111 24481252 PMC3926056

[50] van den Ent F , Amos LA , Löwe J . 2001. Prokaryotic origin of the actin cytoskeleton. Nature 413:39–44. doi:10.1038/35092500 11544518

[51] Hurley JH . 1996. The sugar kinase/heat shock protein 70/actin superfamily: implications of conserved structure for mechanism. Annu Rev Biophys Biomol Struct 25:137–162. doi:10.1146/annurev.bb.25.060196.001033 8800467

[52] van den Ent F , Löwe J . 2000. Crystal structure of the cell division protein FtsA from Thermotoga maritima. EMBO J 19:5300–5307. doi:10.1093/emboj/19.20.5300 11032797 PMC313995

[53] Bork P , Sander C , Valencia A . 1992. An ATPase domain common to prokaryotic cell cycle proteins, sugar kinases, actin, and hsp70 heat shock proteins. Proc Natl Acad Sci U S A 89:7290–7294. doi:10.1073/pnas.89.16.7290 1323828 PMC49695

[54] van den Ent F , Johnson CM , Persons L , de Boer P , Löwe J . 2010. Bacterial actin MreB assembles in complex with cell shape protein RodZ. EMBO J 29:1081–1090. doi:10.1038/emboj.2010.9 20168300 PMC2845281

[55] Bergeron JRC , Hutto R , Ozyamak E , Hom N , Hansen J , Draper O , Byrne ME , Keyhani S , Komeili A , Kollman JM . 2017. Structure of the magnetosome-associated actin-like MamK filament at subnanometer resolution. Protein Sci 26:93–102. doi:10.1002/pro.2979 27391173 PMC5192964

[56] Winkel M , Salman-Carvalho V , Woyke T , Richter M , Schulz-Vogt HN , Flood BE , Bailey JV , Mußmann M . 2016. Single-cell sequencing of Thiomargarita reveals genomic flexibility for adaptation to dynamic redox conditions. Front Microbiol 7:964. doi:10.3389/fmicb.2016.00964 27446006 PMC4914600

[57] Gureeva MV , Belousova EV , Dubinina GA , Novikov AA , Kopitsyn DS , Grabovich MY . 2019. Thioflexithrix psekupsensis gen. nov., sp. nov., a filamentous gliding sulfur bacterium from the family Beggiatoaceae. Int J Syst Evol Microbiol 69:798–804. doi:10.1099/ijsem.0.003240 30657444

[58] Kojima H , Ogura Y , Yamamoto N , Togashi T , Mori H , Watanabe T , Nemoto F , Kurokawa K , Hayashi T , Fukui M . 2015. Ecophysiology of Thioploca ingrica as revealed by the complete genome sequence supplemented with proteomic evidence. ISME J 9:1166–1176. doi:10.1038/ismej.2014.209 25343513 PMC4409161

[59] Schnaars V , Wöhlbrand L , Scheve S , Hinrichs C , Reinhardt R , Rabus R . 2021. Proteogenomic insights into the physiology of marine, sulfate-reducing, filamentous Desulfonema limicola and Desulfonema magnum. Microb Physiol 31:1–20. doi:10.1159/000513383 PMC8315694 33611323

[60] Lapidus A , Nolan M , Lucas S , Glavina Del Rio T , Tice H , Cheng JF , Tapia R , Han C , Goodwin L , Pitluck S , Liolios K , Pagani I , Ivanova N , Huntemann M , Mavromatis K , Mikhailova N , Pati A , Chen A , Palaniappan K , Land M , Brambilla EM , Rohde M , Abt B , Verbarg S , Göker M , Bristow J , Eisen JA , Markowitz V , Hugenholtz P , Kyrpides NC , Klenk HP , Woyke T . 2011. Genome sequence of the filamentous, gliding Thiothrix nivea neotype strain (JP2^T^). Stand Genomic Sci 5:398–406. doi:10.4056/sigs.2344929 22675589 PMC3368414

[61] Kolinko S , Richter M , Glöckner F-O , Brachmann A , Schüler D . 2016. Single-cell genomics of uncultivated deep-branching magnetotactic bacteria reveals a conserved set of magnetosome genes. Environ Microbiol 18:21–37. doi:10.1111/1462-2920.12907 26060021

[62] Wenter R , Wanner G , Schüler D , Overmann J . 2009. Ultrastructure, tactic behaviour and potential for sulfate reduction of a novel multicellular magnetotactic prokaryote from North Sea sediments. Environ Microbiol 11:1493–1505. doi:10.1111/j.1462-2920.2009.01877.x 19220395

[63] Anil Kumar P , Srinivas TNR , Thiel V , Tank M , Sasikala C , Ramana CV , Imhoff JF . 2009. Thiohalocapsa marina sp. nov., from an Indian marine aquaculture pond. Int J Syst Evol Microbiol 59:2333–2338. doi:10.1099/ijs.0.003053-0 19620368

[64] Tanaka N , Romanenko LA , Iino T , Frolova GM , Mikhailov VV . 2011. Cocleimonas flava gen. nov., sp. nov., a gammaproteobacterium isolated from sand snail (Umbonium costatum). Int J Syst Evol Microbiol 61:412–416. doi:10.1099/ijs.0.020263-0 20348322

[65] Lin W , Zhang W , Paterson GA , Zhu Q , Zhao X , Knight R , Bazylinski DA , Roberts AP , Pan Y . 2020. Expanding magnetic organelle biogenesis in the domain Bacteria. Microbiome 8:152. doi:10.1186/s40168-020-00931-9 33126926 PMC7602337

[66] Macalady JL , Lyon EH , Koffman B , Albertson LK , Meyer K , Galdenzi S , Mariani S . 2006. Dominant microbial populations in limestone-corroding stream biofilms, Frasassi cave system, Italy. Appl Environ Microbiol 72:5596–5609. doi:10.1128/AEM.00715-06 16885314 PMC1538711

[67] Bizic M , Brad T , Ionescu D , Barbu-Tudoran L , Zoccarato L , Aerts JW , Contarini P-E , Gros O , Volland J-M , Popa R , Ody J , Vellone D , Flot J-F , Tighe S , Sarbu SM . 2023. Cave Thiovulum (Candidatus Thiovulum stygium) differs metabolically and genomically from marine species. ISME J 17:340–353. doi:10.1038/s41396-022-01350-4 36528730 PMC9938260

[68] Sylvestre M-N , Jean-Louis P , Grimonprez A , Bilas P , Collienne A , Azède C , Gros O . 2021. Candidatus Thiovulum sp. strain imperiosus: the largest free-living Epsilonproteobacteraeota Thiovulum strain lives in a marine mangrove environment. Can J Microbiol 68:1–14. doi:10.1139/cjm-2021-0101 34461021

[69] Ionescu D , Zoccarato L , Zaduryan A , Schorn S , Bizic M , Pinnow S , Cypionka H , Grossart H-P . 2020. Heterozygous, polyploid, giant bacterium, Achromatium, possesses an identical functional inventory worldwide across drastically different ecosystems. Mol Biol Evol 38:1040–1059. doi:10.1093/molbev/msaa273 PMC7947748 33169788

[70] Cuadrado A , Rojo AI , Wells G , Hayes JD , Cousin SP , Rumsey WL , Attucks OC , Franklin S , Levonen A-L , Kensler TW , Dinkova-Kostova AT . 2019. Therapeutic targeting of the NRF2 and KEAP1 partnership in chronic diseases. Nat Rev Drug Discov 18:295–317. doi:10.1038/s41573-018-0008-x 30610225

[71] Shi X , Xiang S , Cao J , Zhu H , Yang B , He Q , Ying M . 2019. Kelch-like proteins: physiological functions and relationships with diseases. Pharmacol Res 148:104404. doi:10.1016/j.phrs.2019.104404 31442578

[72] Hara T , Ishida H , Raziuddin R , Dorkhom S , Kamijo K , Miki T . 2004. Novel kelch-like protein, KLEIP, is involved in actin assembly at cell-cell contact sites of Madin-Darby canine kidney cells. Mol Biol Cell 15:1172–1184. doi:10.1091/mbc.e03-07-0531 14668487 PMC363103

[73] Sasagawa K , Matsudo Y , Kang M , Fujimura L , Iitsuka Y , Okada S , Ochiai T , Tokuhisa T , Hatano M . 2002. Identification of Nd1, a novel murine kelch family protein, involved in stabilization of actin filaments. J Biol Chem 277:44140–44146. doi:10.1074/jbc.M202596200 12213805

[74] Schmid MF , Matsudaira P , Jeng TW , Jakana J , Towns-Andrews E , Bordas J , Chiu W . 1991. Crystallographic analysis of acrosomal bundle from Limulus sperm. J Mol Biol 221:711–725. doi:10.1016/0022-2836(91)80082-6 1920441

[75] Adams J , Kelso R , Cooley L . 2000. The kelch repeat superfamily of proteins: propellers of cell function. Trends Cell Biol 10:17–24. doi:10.1016/s0962-8924(99)01673-6 10603472

[76] Li X , Zhang D , Hannink M , Beamer LJ . 2004. Crystal structure of the Kelch domain of human Keap1. J Biol Chem 279:54750–54758. doi:10.1074/jbc.M410073200 15475350

[77] Snider J , Lin F , Zahedi N , Rodionov V , Yu CC , Gross SP . 2004. Intracellular actin-based transport: how far you go depends on how often you switch. Proc Natl Acad Sci U S A 101:13204–13209. doi:10.1073/pnas.0403092101 15331778 PMC516548

[78] Rodrigues-Oliveira T , Wollweber F , Ponce-Toledo RI , Xu J , Rittmann SK-MR , Klingl A , Pilhofer M , Schleper C . 2023. Actin cytoskeleton and complex cell architecture in an Asgard archaeon. Nature 613:332–339. doi:10.1038/s41586-022-05550-y 36544020 PMC9834061

[79] Korotkov KV , Sandkvist M , Hol WGJ . 2012. The type II secretion system: biogenesis, molecular architecture and mechanism. Nat Rev Microbiol 10:336–351. doi:10.1038/nrmicro2762 22466878 PMC3705712

[80] Naskar S , Hohl M , Tassinari M , Low HH . 2021. The structure and mechanism of the bacterial type II secretion system. Mol Microbiol 115:412–424. doi:10.1111/mmi.14664 33283907

[81] De Masi LG , Sturey CD , Lieberman JA , Donnenberg MS . 2013. Chapter 13 - the type 2 secretion and type 4 Pilus systems of Escherichia coli, p 387–416. In Donnenberg MS (ed), Escherichia coli, Second Edition. Academic Press, Boston. doi:10.1016/B978-0-12-397048-0.00013-9

[82] Denise R , Abby SS , Rocha EPC . 2019. Diversification of the type IV filament superfamily into machines for adhesion, protein secretion, DNA uptake, and motility. PLoS Biol 17:e3000390. doi:10.1371/journal.pbio.3000390 31323028 PMC6668835

[83] Wang F , Gu Y , O’Brien JP , Yi SM , Yalcin SE , Srikanth V , Shen C , Vu D , Ing NL , Hochbaum AI , Egelman EH , Malvankar NS . 2019. Structure of microbial nanowires reveals stacked hemes that transport electrons over micrometers. Cell 177:361–369. doi:10.1016/j.cell.2019.03.029 30951668 PMC6720112

[84] Filman DJ , Marino SF , Ward JE , Yang L , Mester Z , Bullitt E , Lovley DR , Strauss M . 2019. Cryo-EM reveals the structural basis of long-range electron transport in a cytochrome-based bacterial nanowire. Commun Biol 2:219. doi:10.1038/s42003-019-0448-9 31240257 PMC6584659

[85] Gu Y , Srikanth V , Salazar-Morales AI , Jain R , O’Brien JP , Yi SM , Soni RK , Samatey FA , Yalcin SE , Malvankar NS . 2021. Structure of Geobacter pili reveals secretory rather than nanowire behaviour. Nature 597:430–434. doi:10.1038/s41586-021-03857-w 34471289 PMC9127704

[86] Boschker HTS , Cook PLM , Polerecky L , Eachambadi RT , Lozano H , Hidalgo-Martinez S , Khalenkow D , Spampinato V , Claes N , Kundu P , Wang D , Bals S , Sand KK , Cavezza F , Hauffman T , Bjerg JT , Skirtach AG , Kochan K , McKee M , Wood B , Bedolla D , Gianoncelli A , Geerlings NMJ , Van Gerven N , Remaut H , Geelhoed JS , Millan-Solsona R , Fumagalli L , Nielsen LP , Franquet A , Manca JV , Gomila G , Meysman FJR . 2021. Efficient long-range conduction in cable bacteria through nickel protein wires. Nat Commun 12:3996. doi:10.1038/s41467-021-24312-4 34183682 PMC8238962

[87] Berne C , Ellison CK , Ducret A , Brun YV . 2018. Bacterial adhesion at the single-cell level. Nat Rev Microbiol 16:616–627. doi:10.1038/s41579-018-0057-5 30008468

[88] Lu HM , Motley ST , Lory S . 1997. Interactions of the components of the general secretion pathway: role of Pseudomonas aeruginosa type IV pilin subunits in complex formation and extracellular protein secretion. Mol Microbiol 25:247–259. doi:10.1046/j.1365-2958.1997.4561818.x 9282737

[89] Mattick JS . 2002. Type IV pili and twitching motility. Annu Rev Microbiol 56:289–314. doi:10.1146/annurev.micro.56.012302.160938 12142488

[90] Persat A , Inclan YF , Engel JN , Stone HA , Gitai Z . 2015. Type IV pili mechanochemically regulate virulence factors in Pseudomonas aeruginosa. Proc Natl Acad Sci U S A 112:7563–7568. doi:10.1073/pnas.1502025112 26041805 PMC4475988

[91] Khayatan B , Meeks JC , Risser DD . 2015. Evidence that a modified type IV pilus-like system powers gliding motility and polysaccharide secretion in filamentous cyanobacteria. Mol Microbiol 98:1021–1036. doi:10.1111/mmi.13205 26331359

[92] Varga JJ , Nguyen V , O’Brien DK , Rodgers K , Walker RA , Melville SB . 2006. Type IV pili-dependent gliding motility in the gram-positive pathogen Clostridium perfringens and other Clostridia. Mol Microbiol 62:680–694. doi:10.1111/j.1365-2958.2006.05414.x 16999833

[93] Giovannoni S . 1991. The polymerase chain reaction, p 175–201. In Stackebrandt E , M Goodfellow (ed), Nucleic acid techniques in bacterial systematics. John Wiley & Sons, New York.

[94] Loy A , Schulz C , Lücker S , Schöpfer-Wendels A , Stoecker K , Baranyi C , Lehner A , Wagner M . 2005. 16S rRNA gene-based oligonucleotide microarray for environmental monitoring of the betaproteobacterial order "Rhodocyclales". Appl Environ Microbiol 71:1373–1386. doi:10.1128/AEM.71.3.1373-1386.2005 15746340 PMC1065177

[95] Geelhoed JS , van de Velde SJ , Meysman FJR . 2020. Quantification of cable bacteria in marine sediments via qPCR. Front Microbiol 11:1506. doi:10.3389/fmicb.2020.01506 32719667 PMC7348212

[96] Kjeldsen KU , Loy A , Jakobsen TF , Thomsen TR , Wagner M , Ingvorsen K . 2007. Diversity of sulfate-reducing bacteria from an extreme hypersaline sediment, Great Salt Lake (Utah). FEMS Microbiol Ecol 60:287–298. doi:10.1111/j.1574-6941.2007.00288.x 17367515

[97] Hongoh Y , Ohkuma M , Kudo T . 2003. Molecular analysis of bacterial microbiota in the gut of the termite Reticulitermes speratus (Isoptera; Rhinotermitidae). FEMS Microbiol Ecol 44:231–242. doi:10.1016/S0168-6496(03)00026-6 19719640

[98] Bolger AM , Lohse M , Usadel B . 2014. Trimmomatic: a flexible trimmer for Illumina sequence data. Bioinform 30:2114–2120. doi:10.1093/bioinformatics/btu170 PMC4103590 24695404

[99] Bankevich A , Nurk S , Antipov D , Gurevich AA , Dvorkin M , Kulikov AS , Lesin VM , Nikolenko SI , Pham S , Prjibelski AD , Pyshkin AV , Sirotkin AV , Vyahhi N , Tesler G , Alekseyev MA , Pevzner PA . 2012. SPAdes: a new genome assembly algorithm and its applications to single-cell sequencing. J Comput Biol 19:455–477. doi:10.1089/cmb.2012.0021 22506599 PMC3342519

[100] Strous M , Kraft B , Bisdorf R , Tegetmeyer HE . 2012. The binning of rnetagenomic contings for physiology of mixed cultures. Front Microbiol 3:410. doi:10.3389/fmicb.2012.00410 23227024 PMC3514610

[101] Richter M , Rosselló-Móra R . 2009. Shifting the genomic gold standard for the prokaryotic species definition. Proc Natl Acad Sci U S A 106:19126–19131. doi:10.1073/pnas.0906412106 19855009 PMC2776425

[102] Parks DH , Imelfort M , Skennerton CT , Hugenholtz P , Tyson GW . 2015. CheckM: assessing the quality of microbial genomes recovered from isolates, single cells, and metagenomes. Genome Res 25:1043–1055. doi:10.1101/gr.186072.114 25977477 PMC4484387

[103] Hyatt D , Chen G-L , Locascio PF , Land ML , Larimer FW , Hauser LJ . 2010. Prodigal: prokaryotic gene recognition and translation initiation site identification. BMC Bioinformatics 11:119. doi:10.1186/1471-2105-11-119 20211023 PMC2848648

[104] Seemann T . 2014. Prokka: rapid prokaryotic genome annotation. Bioinform 30:2068–2069. doi:10.1093/bioinformatics/btu153 24642063

[105] Goris J , Konstantinidis KT , Klappenbach JA , Coenye T , Vandamme P , Tiedje JM . 2007. DNA–DNA hybridization values and their relationship to whole-genome sequence similarities. Int J Syst Evol Microbiol 57:81–91. doi:10.1099/ijs.0.64483-0 17220447

[106] Rodriguez-R LM , Konstantinidis KT . 2016. The enveomics collection: a toolbox for specialized analyses of microbial genomes and metagenomes. Peerj Preprints. doi:10.7287/peerj.preprints.1900v1

[107] Bobay L-M , Ellis B-H , Ochman H . 2018. ConSpeciFix: classifying prokaryotic species based on gene flow. Bioinform 34:3738–3740. doi:10.1093/bioinformatics/bty400 PMC6198855 29771275

[108] Edgar RC . 2010. Search and clustering orders of magnitude faster than BLAST. Bioinform 26:2460–2461. doi:10.1093/bioinformatics/btq461 20709691

[109] Csurös M . 2010. Count: evolutionary analysis of phylogenetic profiles with parsimony and likelihood. Bioinform 26:1910–1912. doi:10.1093/bioinformatics/btq315 20551134

[110] Teufel F , Almagro Armenteros JJ , Johansen AR , Gíslason MH , Pihl SI , Tsirigos KD , Winther O , Brunak S , von Heijne G , Nielsen H . 2022. SignalP 6.0 predicts all five types of signal peptides using protein language models. Nat Biotechnol 40:1023–1025. doi:10.1038/s41587-021-01156-3 34980915 PMC9287161

[111] Krogh A , Larsson B , von Heijne G , Sonnhammer EL . 2001. Predicting transmembrane protein topology with a hidden Markov model: application to complete genomes. J Mol Biol 305:567–580. doi:10.1006/jmbi.2000.4315 11152613

[112] Camacho C , Coulouris G , Avagyan V , Ma N , Papadopoulos J , Bealer K , Madden TL . 2009. BLAST+: architecture and applications. BMC Bioinformatics 10:421. doi:10.1186/1471-2105-10-421 20003500 PMC2803857

[113] Marchler-Bauer A , Bo Y , Han L , He J , Lanczycki CJ , Lu S , Chitsaz F , Derbyshire MK , Geer RC , Gonzales NR , Gwadz M , Hurwitz DI , Lu F , Marchler GH , Song JS , Thanki N , Wang Z , Yamashita RA , Zhang D , Zheng C , Geer LY , Bryant SH . 2017. CDD/SPARCLE: functional classification of proteins via subfamily domain architectures. Nuc Ac Res 45:D200–D203. doi:10.1093/nar/gkw1129 PMC5210587 27899674

[114] Cantalapiedra CP , Hernández-Plaza A , Letunic I , Bork P , Huerta-Cepas J . 2021. eggNOG-mapper v2: functional annotation, orthology assignments, and domain prediction at the metagenomic scale. Mol Biol Evol 38:5825–5829. doi:10.1093/molbev/msab293 34597405 PMC8662613

[115] Lagesen K , Hallin P , Rødland EA , Staerfeldt H-H , Rognes T , Ussery DW . 2007. RNAmmer: consistent and rapid annotation of ribosomal RNA genes. Nuc Ac Res 35:3100–3108. doi:10.1093/nar/gkm160 PMC1888812 17452365

[116] Edgar RC . 2004. MUSCLE: multiple sequence alignment with high accuracy and high throughput. Nuc Ac Res 32:1792–1797. doi:10.1093/nar/gkh340 PMC390337 15034147

[117] Nguyen L-T , Schmidt HA , von Haeseler A , Minh BQ . 2015. IQ-TREE: a fast and effective stochastic algorithm for estimating maximum-likelihood phylogenies. Mol Biol Evol 32:268–274. doi:10.1093/molbev/msu300 25371430 PMC4271533

[118] Chaumeil P-A , Mussig AJ , Hugenholtz P , Parks DH . 2019. GTDB-Tk: a toolkit to classify genomes with the genome taxonomy database. Bioinform 36:1925–1927. doi:10.1093/bioinformatics/btz848 PMC7703759 31730192

[119] El-Gebali S , Mistry J , Bateman A , Eddy SR , Luciani A , Potter SC , Qureshi M , Richardson LJ , Salazar GA , Smart A , Sonnhammer ELL , Hirsh L , Paladin L , Piovesan D , Tosatto SCE , Finn RD . 2019. The Pfam protein families database in 2019. Nuc Ac Res 47:D427–D432. doi:10.1093/nar/gky995 PMC6324024 30357350

[120] Derman AI , Becker EC , Truong BD , Fujioka A , Tucey TM , Erb ML , Patterson PC , Pogliano J . 2009. Phylogenetic analysis identifies many uncharacterized actin-like proteins (Alps) in bacteria: regulated polymerization, dynamic instability and treadmilling in Alp7A. Mol Microbiol 73:534–552. doi:10.1111/j.1365-2958.2009.06771.x 19602153 PMC2814180

[121] Ludwig W , Strunk O , Westram R , Richter L , Meier H , Yadhukumar , Buchner A , Lai T , Steppi S , Jobb G , Förster W , Brettske I , Gerber S , Ginhart AW , Gross O , Grumann S , Hermann S , Jost R , König A , Liss T , Lüssmann R , May M , Nonhoff B , Reichel B , Strehlow R , Stamatakis A , Stuckmann N , Vilbig A , Lenke M , Ludwig T , Bode A , Schleifer K-H . 2004. ARB: a software environment for sequence data. Nucleic Acids Res 32:1363–1371. doi:10.1093/nar/gkh293 14985472 PMC390282

[122] Quast C , Pruesse E , Yilmaz P , Gerken J , Schweer T , Yarza P , Peplies J , Glöckner FO . 2013. The SILVA ribosomal RNA gene database project: improved data processing and web-based tools. Nuc Ac Res 41:D590–D596. doi:10.1093/nar/gks1219 PMC3531112 23193283

[123] Yilmaz LS , Parnerkar S , Noguera DR . 2011. mathFISH, a web tool that uses thermodynamics-based mathematical models for in silico evaluation of oligonucleotide probes for fluorescence in situ hybridization. Appl Environ Microbiol 77:1118–1122. doi:10.1128/AEM.01733-10 21148691 PMC3028703

[124] Pernthaler J , Glöckner F-O , Schönhuber W , Amann R . 2001. Fluorescence in situ hybridization (FISH) with rRNA-targeted oligonucleotide probes. Methods in Microbiology 30:207–226. doi:10.1016/S0580-9517(01)30046-6

[125] Schindelin J , Arganda-Carreras I , Frise E , Kaynig V , Longair M , Pietzsch T , Preibisch S , Rueden C , Saalfeld S , Schmid B , Tinevez J-Y , White DJ , Hartenstein V , Eliceiri K , Tomancak P , Cardona A . 2012. Fiji: an open-source platform for biological-image analysis. Nat Methods 9:676–682. doi:10.1038/nmeth.2019 22743772 PMC3855844

