# Supplementary material for: Indications for a genetic basis for big bacteria and description of the giant cable bacterium Candidatus Electrothrix gigas sp. nov
Source: Microbiol Spectr. 2023 Sep 21;11(5):e00538-23. doi: 10.1128/spectrum.00538-23 (PMC10580974; doi:10.1128/spectrum.00538-23)
Supplement: Supplemental Material — Tables S1–S5, Figures S1–S7 and Legend of Video S1. [file spectrum.00538-23-s0001.pdf]

Supplemental Material

Indications for a Genetic Basis for Big Bacteria and Description of the Giant Cable Bacterium *Candidatus* Electrothrix gigas sp. nov.

Jeanine S. Geelhoed,[a] Casper A. Thorup,[b] Jesper J. Bjerg,[a,b] Lars Schreiber,[b*] Lars Peter Nielsen,[b] Andreas Schramm,[b] Filip J.R. Meysman,[a,c#] Ian P.G. Marshall[b]

[a]Department of Biology, Research Group Geobiology, University of Antwerp, Wilrijk, Belgium

[b]Department of Biology, Center for Electromicrobiology, Aarhus University, Aarhus, Denmark

[c]Department of Biotechnology, Delft University of Technology, Delft, The Netherlands

[#]Address correspondence to Filip J.R. Meysman, Filip.Meysman@uantwerpen.be

*present address: Lars Schreiber, Energy, Mining and Environment Research Centre, National Research Council Canada, Montreal, Canada

Supplementary Tables S1 to S5

Supplementary Figures S1 to S7

Legend to Supplementary Video S1

Table S1 Properties of cable bacteria genomes

Genome definition	Sediment enrichment culture	Genomes for co-assembly	ANI (%)*	Assembly size (Mb)	Number of contigs	N50 (kb)	Min.contig length (kb)	Max.contig length (kb)	Genome completeness (%)[#]	Contamination (%)[#]	Reference
Candidatus Electrothrix gigas strain AS4_5	Y	2	99.84	3.22	808	5.63	1.00	23.55	92.1	3.4	This study
Candidatus Electrothrix gigas strain AU1-5	Y	5	99.85	3.60	163	42.29	1.00	161.49	97.1	2.7	This study
Candidatus Electrothrix gigas strain AUS3	Y			2.71	998	3.15	1.00	18.84	78.1	3.6	This study
Candidatus Electrothrix gigas strain AW1	Y			3.39	407	15.19	1.02	57.25	96.8	3.0	This study
Candidatus Electrothrix gigas strain AW2	Y			3.35	696	7.07	1.00	34.82	90.7	2.2	This study
Candidatus Electrothrix gigas strain AW3_4	Y	2	99.87	3.47	354	16.03	1.00	72.48	95.9	3.8	This study
Candidatus Electrothrix gigas strain AW5	Y			3.35	444	12.45	1.02	78.37	94.7	4.6	This study
Candidatus Electrothrix gigas strain AX1_4	Y	2	99.82	3.43	320	17.83	1.02	62.79	96.6	2.2	This study
Candidatus Electrothrix gigas strain AX2	Y			3.15	818	5.35	1.00	21.19	87.8	3.7	This study
Candidatus Electrothrix gigas strain LOE1_4_5	N	3	99.81	3.15	784	5.51	1.00	31.88	86.0	3.3	This study
Candidatus Electrothrix aarhusiensis filament AX5	Y			3.80	1174	4.06	1.00	19.83	80.1	2.5	This study
Candidatus Electrothrix sp. AR1	Y			2.60	1402	1.94	1.00	8.03	43.5	1.2	This study
Candidatus Electrothrix sp. AR5	Y			3.54	1341	3.10	1.00	15.10	73.5	3.0	This study
Candidatus Electrothrix sp. ATG1_2 filament ATG1	Y			3.10	1441	2.33	1.00	11.76	65.4	2.7	This study
Candidatus Electrothrix sp. ATG1_2 filament ATG2	Y			4.41	2229	2.09	1.00	12.80	63.8	14.7	This study
Candidatus Electrothrix sp. AUS1_2	Y	2	99.61	2.79	1349	2.25	1.00	12.13	59.1	1.2	This study
Candidatus Electrothrix sp. AUS4	Y			2.52	1393	1.87	1.00	8.94	50.9	2.5	This study
Candidatus Electrothrix sp. EH2	N			2.80	1297	2.36	1.00	12.61	55.0	2.2	This study
Candidatus Electrothrix sp. GM3_4	N	2	99.75	4.09	1190	4.36	1.00	29.42	86.5	2.7	This study
Candidatus Electrothrix sp. LOE2	N			2.82	1070	3.12	1.00	19.56	71.2	2.6	This study
Candidatus Electrothrix sp. MAN1_4	N	2	99.77	3.13	1177	3.18	1.00	13.30	76.9	2.4	This study
Desulfobulbaceae cable bacterium filament AR4	Y			3.71	1246	3.54	1.00	13.16	80.2	3.1	This study
Desulfobulbaceae cable bacterium filament AR3	Y			3.29	1561	2.30	1.00	10.83	69.6	2.3	This study
Candidatus Electrothrix aarhusiensis isolate MCF	Y			3.73	143	44.35	1.11	136.49	92.5	1.1	Trojan et al. 2016; Kjeldsen et al. 2019
Candidatus Electrothrix marina isolate A2	Y			0.99	450	2.42	0.53	13.78	36.3	0.4	Trojan et al. 2016; Kjeldsen et al. 2019
Candidatus Electrothrix marina isolate A3	Y			0.60	349	1.85	0.58	5.01	20.8	0.0	Trojan et al. 2016; Kjeldsen et al. 2019
Candidatus Electrothrix marina isolate A5	Y			2.06	472	5.73	0.59	25.25	63.6	2.3	Trojan et al. 2016; Kjeldsen et al. 2019
Candidatus Electrothrix communis isolate A1	Y			1.07	489	2.48	0.63	7.96	34.0	1.6	Trojan et al. 2016; Kjeldsen et al. 2019
Candidatus Electrothrix communis isolate N2	Y			0.84	462	1.93	0.56	6.36	31.8	0.3	Trojan et al. 2016; This study
Candidatus Electrothrix communis isolate N3	Y			0.82	448	1.93	0.54	6.77	30.5	0.0	Trojan et al. 2016; This study
Candidatus Electrothrix communis isolate US1	Y			1.31	605	2.39	0.53	8.36	50.7	1.1	Trojan et al. 2016; This study
Candidatus Electrothrix communis isolate US2	Y			1.31	573	2.50	0.63	13.85	50.1	0.3	Trojan et al. 2016; This study
Candidatus Electrothrix communis isolate US4	Y			3.40	307	17.82	0.78	64.63	87.7	1.6	Trojan et al. 2016; This study
Candidatus Electrothrix communis isolate US5	Y			0.97	448	2.34	0.65	9.89	38.9	0.0	Trojan et al. 2016; This study
Candidatus Electrothrix japonica isolate TB	Y			2.58	539	7.23	0.51	37.05	72.6	4.4	Trojan et al. 2016; This study
Candidatus Electronema aureum isolate GS	Y			2.76	73	58.41	1.12	231.37	92.9	1.6	Kjeldsen et al. 2019
Candidatus Electronema nielsenii isolate F1	Y			0.91	348	3.08	0.62	11.98	38.1	0.0	Trojan et al. 2016; This study
Candidatus Electronema nielsenii isolate F5	Y			1.76	242	11.51	0.94	41.75	65.7	0.8	Trojan et al. 2016; This study
Candidatus Electronema palustre isolate F3	Y			0.81	330	3.11	0.62	15.35	30.3	1.5	Trojan et al. 2016; This study
Candidatus Electronema palustre isolate F4	Y			0.90	384	2.71	0.58	10.84	35.5	0.5	Trojan et al. 2016; This study

*calculated with JSpecies v1.2.1 option ANIm (Richter & Rosselló-Móra 2009 Proc Natl Acad Sci USA, 106: 19126-31)

[#]estimated with CheckM v1.0.7 using the reference dataset for the order *Desulfobacterales* (Parks et al. 2014 Genome Res, 25: 1043-1055)

Table S2 Whole-genome average nucleotide identity (%) values for *Ca*. Electrothrix genomes. Colored cells indicate values > 95%, above the proposed species-delineation threshold (Konstantinidis et al., 2017).

name	AUS3	AW2	AW3_4	AU1-5	AW1	AW5	AX2	LOE1_4	AX1_4	AS4_5	TB	MAN1_	ATG1	ATG2	A1	N2	N3	US1	US2	US4	US5
AUS3	100	97.63	97.59	97.57	97.67	97.6	97.76	97.66	97.69	98.5	89.82	81.51	81.98	81.96	80.36	80.65	80.83	80.75	80.18	79.67	81.42
AW2	97.63	100	98.02	97.84	98	98.04	98.07	98.09	98.02	97.46	89.55	81.28	82.29	81.69	80.1	80.46	80.46	80.5	80.31	79.62	80.82
AW3_4	97.59	98.02	100	97.83	98.05	98.1	98.07	98.06	98.03	97.54	89.67	81.28	81.96	81.81	80.35	80.73	80.84	80.68	80.41	79.63	80.97
AU1-5	97.57	97.84	97.83	100	97.78	97.78	97.88	97.88	97.8	97.38	89.46	80.95	81.47	81.38	80.12	80.02	80.58	80.34	80.14	79.55	80.61
AW1	97.67	98	98.05	97.78	100	98.05	98.11	98.21	99.94	97.5	89.69	81.18	82.13	81.41	80.13	80.72	81.08	80.79	80.31	79.66	81.12
AW5	97.6	98.04	98.1	97.78	98.05	100	98.07	98.11	98.03	97.48	89.7	81.09	81.87	81.46	80.31	80.52	80.44	80.41	80.22	79.48	80.79
AX2	97.76	98.07	98.07	97.88	98.11	98.07	100	98.11	98.09	97.48	89.8	81.54	82.23	81.54	80.36	80.93	80.53	80.8	80.03	79.73	80.98
LOE1_4_5	97.66	98.09	98.06	97.88	98.21	98.11	98.11	100	98.19	97.49	89.76	81.21	82.07	82.04	80.29	80.33	80.53	80.5	80.19	79.51	81.02
AX1_4	97.69	98.02	98.03	97.8	99.94	98.03	98.09	98.19	100	97.49	89.61	81.32	82.13	81.54	80.3	80.67	80.69	80.56	80.32	79.53	80.85
AS4_5	98.5	97.46	97.54	97.38	97.5	97.48	97.48	97.49	97.49	100	89.87	81.08	82.35	81.82	80.32	80.73	80.58	80.46	80.34	79.64	81.41
TB	89.82	89.55	89.67	89.46	89.69	89.7	89.8	89.76	89.61	89.87	100	80.92	80.71	80.46	80.74	80.72	80.84	80.33	80.71	79.63	80.88
MAN1_4	81.51	81.28	81.28	80.95	81.18	81.09	81.54	81.21	81.32	81.08	80.92	100	81.64	81.28	80.67	80.93	80.6	80.69	80.54	79.81	81.23
ATG1	81.98	82.29	81.96	81.47	82.13	81.87	82.23	82.07	82.13	82.35	80.71	81.64	100	97.12	81.14	80.94	80.54	80.66	80.82	80.78	81.47
ATG2	81.96	81.69	81.81	81.38	81.41	81.46	81.54	82.04	81.54	81.82	80.46	81.28	97.12	100	81.35	81.15	81.35	81.18	81.13	80.97	81.76
A1	80.36	80.1	80.35	80.12	80.13	80.31	80.36	80.29	80.3	80.32	80.74	80.67	81.14	81.35	100	99.35	99.42	99.42	99.43	99.33	99.31
N2	80.65	80.46	80.73	80.02	80.72	80.52	80.93	80.33	80.67	80.73	80.72	80.93	80.94	81.15	99.35	100	99.93	99.93	99.95	99.96	99.94
N3	80.83	80.46	80.84	80.58	81.08	80.44	80.53	80.53	80.69	80.58	80.84	80.6	80.54	81.35	99.42	99.93	100	99.92	99.93	99.94	99.92
US1	80.75	80.5	80.68	80.34	80.79	80.41	80.8	80.5	80.56	80.46	80.33	80.69	80.66	81.18	99.42	99.93	99.92	100	99.95	99.96	99.95
US2	80.18	80.31	80.41	80.14	80.31	80.22	80.03	80.19	80.32	80.34	80.71	80.54	80.82	81.13	99.43	99.95	99.93	99.95	100	99.96	99.95
US4	79.67	79.62	79.63	79.55	79.66	79.48	79.73	79.51	79.53	79.64	79.63	79.81	80.78	80.97	99.33	99.96	99.94	99.96	99.96	100	99.96
US5	81.42	80.82	80.97	80.61	81.12	80.79	80.98	81.02	80.85	81.41	80.88	81.23	81.47	81.76	99.31	99.94	99.92	99.95	99.95	99.96	100
AR1	82.14	81.71	81.82	81.72	81.8	81.39	81.61	81.93	81.73	82.36	81.13	81.72	82.98	82.97	88.89	89.2	88.76	89.13	89.05	88.69	89.11
AR5	80.83	80.31	80.33	80.29	80.38	80.28	80.61	80.44	80.16	80.72	80.56	80.5	82	82.46	87.85	87.88	87.48	87.78	87.36	87.44	87.83
AX5	80.48	80.23	80.21	80.14	80.41	80.32	80.28	80.34	80.48	80.31	80.02	80.67	81.49	81.53	86.69	86.66	86.29	86.61	86.17	86.02	86.92
MCF	79.73	79.64	79.87	79.74	79.85	79.67	79.98	79.75	79.86	79.85	79.7	80.08	80.76	80.97	86.38	86.7	86.43	86.51	86.11	86.05	86.6
GM3_4	80.58	80.24	80.11	80.23	80.04	79.99	80.61	80.27	80.17	80.45	80.01	80.39	81.42	81.16	85.43	85.51	85.55	85.68	85.47	84.88	85.72
A2	80.88	80.74	80.71	80.47	80.83	80.56	80.84	80.81	81	80.82	81.17	81.32	81.08	81.53	88.15	88.36	87.97	88.41	88.25	88.29	88.06
A3	81.29	81.22	81.39	81.57	81.48	81.13	80.9	80.91	81.88	81.54	81.58	80.9	80.96	81.72	88.6	88.5	88.36	88.3	88.05	88.15	88.31
A5	79.99	80.05	79.86	79.98	79.96	79.93	80.03	79.76	79.99	79.93	80.35	80.37	81.2	81.52	88.76	88.18	88.2	88.02	87.99	88.51	87.86
LOE2	81.17	80.75	81.15	80.83	81.09	80.94	80.76	80.92	80.97	81.14	80.3	80.93	81.45	81.74	84.22	84.06	83.87	84.12	83.89	83.78	84.41
EH2	82.67	83.64	83.79	83.61	83.65	83.38	83.48	83.08	83.86	82.4	80.11	81.97	81.96	81.74	81.41	81.45	80.88	80.84	80.4	80.27	81.53
AUS1_2	81.21	80.66	80.77	81.18	80.44	80.43	80.98	80.96	80.64	80.78	79.9	81.07	81.52	81.09	79.87	79.81	79.5	79.99	79.79	79.57	80.38
AUS4	82.03	81.2	81.02	80.86	80.88	80.69	81.07	81.47	80.7	81.17	79.36	81.84	81.33	81.08	80.14	80.56	79.77	80.65	79.69	79.38	81.13
AR4	79.79	79.62	78.97	78.96	79.22	79.28	79.39	80.05	78.99	79.74	78.13	80.99	81.94	82.08	78.68	79.6	79.02	79.64	78.95	78.64	79.88
AR3	83.29	82.18	81.76	81.17	81.81	81.64	82.6	82.41	81.29	82.95	78.68	81.61	85.25	85.38	80.96	80.91	80.72	80.22	79.45	80.39	82.68
F1	n.r.	n.r.	n.r.	n.r.	n.r.	n.r.	n.r.	n.r.	n.r.	n.r.	n.r.	n.r.	n.r.	n.r.	n.r.	n.r.	n.r.	n.r.	n.r.	n.r.	n.r.
F5	n.r.	76.05	75.25	75.93	76.54	77.44	77.15	77.62	75.25	76.76	73.36	77.21	77.06	77.94	n.r.	n.r.	n.r.	n.r.	n.r.	75.67	n.r.
GS	76.86	76.46	76.57	75.86	76.37	76.36	76.93	76.68	76.13	77.14	75.21	76.7	76.96	77.6	n.r.	n.r.	n.r.	76.33	76.31	74.93	78.1
F3	n.r.	n.r.	n.r.	n.r.	n.r.	n.r.	n.r.	n.r.	n.r.	n.r.	n.r.	n.r.	n.r.	n.r.	n.r.	n.r.	n.r.	n.r.	n.r.	n.r.	n.r.
F4	n.r.	77.29	75.86	n.r.	77.1	n.r.	n.r.	n.r.	n.r.	n.r.	n.r.	n.r.	n.r.	n.r.	n.r.	n.r.	n.r.	n.r.	n.r.	n.r.	n.r.

name	AR1	AR5	AX5	MCF	GM3_4	A2	A3	A5	LOE2	EH2	AUS1_2	AUS4	AR4	AR3	F1	F5	GS	F3	F4
AUS3	82.14	80.83	80.48	79.73	80.58	80.88	81.29	79.99	81.17	82.67	81.21	82.03	79.79	83.29	n.r.	n.r.	76.86	n.r.	n.r.
AW2	81.71	80.31	80.23	79.64	80.24	80.74	81.22	80.05	80.75	83.64	80.66	81.2	79.62	82.18	n.r.	76.05	76.46	n.r.	77.29
AW3_4	81.82	80.33	80.21	79.87	80.11	80.71	81.39	79.86	81.15	83.79	80.77	81.02	78.97	81.76	n.r.	75.25	76.57	n.r.	75.86
AU1-5	81.72	80.29	80.14	79.74	80.23	80.47	81.57	79.98	80.83	83.61	81.18	80.86	78.96	81.17	n.r.	75.93	75.86	n.r.	n.r.
AW1	81.8	80.38	80.41	79.85	80.04	80.83	81.48	79.96	81.09	83.65	80.44	80.88	79.22	81.81	n.r.	76.54	76.37	n.r.	77.1
AW5	81.39	80.28	80.32	79.67	79.99	80.56	81.13	79.93	80.94	83.38	80.43	80.69	79.28	81.64	n.r.	77.44	76.36	n.r.	n.r.
AX2	81.61	80.61	80.28	79.98	80.61	80.84	80.9	80.03	80.76	83.48	80.98	81.07	79.39	82.6	n.r.	77.15	76.93	n.r.	n.r.
LOE1_4_5	81.93	80.44	80.34	79.75	80.27	80.81	80.91	79.76	80.92	83.08	80.96	81.47	80.05	82.41	n.r.	77.62	76.68	n.r.	n.r.
AX1_4	81.73	80.16	80.48	79.86	80.17	81	81.88	79.99	80.97	83.86	80.64	80.7	78.99	81.29	n.r.	75.25	76.13	n.r.	n.r.
AS4_5	82.36	80.72	80.31	79.85	80.45	80.82	81.54	79.93	81.14	82.4	80.78	81.17	79.74	82.95	n.r.	76.76	77.14	n.r.	n.r.
TB	81.13	80.56	80.02	79.7	80.01	81.17	81.58	80.35	80.3	80.11	79.9	79.36	78.13	78.68	n.r.	73.36	75.21	n.r.	n.r.
MAN1_4	81.72	80.5	80.67	80.08	80.39	81.32	80.9	80.37	80.93	81.97	81.07	81.84	80.99	81.61	n.r.	77.21	76.7	n.r.	n.r.
ATG1	82.98	82	81.49	80.76	81.42	81.08	80.96	81.2	81.45	81.96	81.52	81.33	81.94	85.25	n.r.	77.06	76.96	n.r.	n.r.
ATG2	82.97	82.46	81.53	80.97	81.16	81.53	81.72	81.52	81.74	81.74	81.09	81.08	82.08	85.38	n.r.	77.94	77.6	n.r.	n.r.
A1	88.89	87.85	86.69	86.38	85.43	88.15	88.6	88.76	84.22	81.41	79.87	80.14	78.68	80.96	n.r.	n.r.	n.r.	n.r.	n.r.
N2	89.2	87.88	86.66	86.7	85.51	88.36	88.5	88.18	84.06	81.45	79.81	80.56	79.6	80.91	n.r.	n.r.	n.r.	n.r.	n.r.
N3	88.76	87.48	86.29	86.43	85.55	87.97	88.36	88.2	83.87	80.88	79.5	79.77	79.02	80.72	n.r.	n.r.	n.r.	n.r.	n.r.
US1	89.13	87.78	86.61	86.51	85.68	88.41	88.3	88.02	84.12	80.84	79.99	80.65	79.64	80.22	n.r.	n.r.	76.33	n.r.	n.r.
US2	89.05	87.36	86.17	86.11	85.47	88.25	88.05	87.99	83.89	80.4	79.79	79.69	78.95	79.45	n.r.	n.r.	76.31	n.r.	n.r.
US4	88.69	87.44	86.02	86.05	84.88	88.29	88.15	88.51	83.78	80.27	79.57	79.38	78.64	80.39	n.r.	75.67	74.93	n.r.	n.r.
US5	89.11	87.83	86.92	86.6	85.72	88.06	88.31	87.86	84.41	81.53	80.38	81.13	79.88	82.68	n.r.	n.r.	78.1	n.r.	n.r.
AR1	100	90.23	86.56	86.34	85.41	87.61	87.84	87.36	84.4	81.66	81.27	81.53	83.71	87.18	n.r.	n.r.	77.57	n.r.	n.r.
AR5	90.23	100	85.93	85.65	84.82	86.9	86.65	86.56	83.93	81.15	80.49	81.24	81.09	84.57	n.r.	77.44	76.3	n.r.	n.r.
AX5	86.56	85.93	100	97.49	86.31	86.2	86.77	86.24	84	80.94	80.25	80.58	80.27	83.15	79.27	77.3	75.27	n.r.	n.r.
MCF	86.34	85.65	97.49	100	86.27	86.55	86.32	86.21	83.82	80.45	79.67	79.62	78.29	81.22	77.63	76.32	74.86	n.r.	77.31
GM3_4	85.41	84.82	86.31	86.27	100	85.51	85.13	84.99	83.31	80.75	79.7	80.53	80.19	83.29	79	76.25	75.79	n.r.	n.r.
A2	87.61	86.9	86.2	86.55	85.51	100	99.26	99.46	85.74	81.7	80.9	81.09	79.37	78.88	n.r.	n.r.	76.72	n.r.	n.r.
A3	87.84	86.65	86.77	86.32	85.13	99.26	100	99.93	85.53	81.45	80.45	81.48	81.76	n.r.	n.r.	n.r.	n.r.	n.r.	n.r.
A5	87.36	86.56	86.24	86.21	84.99	99.46	99.93	100	85.66	81.32	80.49	80.39	78.23	79.38	n.r.	76.16	75.96	n.r.	n.r.
LOE2	84.4	83.93	84	83.82	83.31	85.74	85.53	85.66	100	82.41	82.25	81.62	80.72	82.33	79.24	76.52	77.07	n.r.	n.r.
EH2	81.66	81.15	80.94	80.45	80.75	81.7	81.45	81.32	82.41	100	82.86	82.31	80.22	82.6	n.r.	77.23	76.46	n.r.	n.r.
AUS1_2	81.27	80.49	80.25	79.67	79.7	80.9	80.45	80.49	82.25	82.86	100	83.97	80.88	82.86	n.r.	77.38	77.45	n.r.	n.r.
AUS4	81.53	81.24	80.58	79.62	80.53	81.09	81.48	80.39	81.62	82.31	83.97	100	82.77	84.8	n.r.	n.r.	78.86	n.r.	n.r.
AR4	83.71	81.09	80.27	78.29	80.19	79.37	81.76	78.23	80.72	80.22	80.88	82.77	100	82.51	n.r.	76.6	75.52	n.r.	n.r.
AR3	87.18	84.57	83.15	81.22	83.29	78.88	n.r.	79.38	82.33	82.6	82.86	84.8	82.51	100	76.92	74.88	75.51	n.r.	n.r.
F1	n.r.	n.r.	79.27	77.63	79	n.r.	n.r.	n.r.	79.24	n.r.	n.r.	n.r.	n.r.	76.92	100	94.98	93.98	79.43	80.98
F5	n.r.	77.44	77.3	76.32	76.25	n.r.	n.r.	76.16	76.52	77.23	77.38	n.r.	76.6	74.88	94.98	100	93.32	82.28	82.76
GS	77.57	76.3	75.27	74.86	75.79	76.72	n.r.	75.96	77.07	76.46	77.45	78.86	75.52	75.51	93.98	93.32	100	82.77	83.1
F3	n.r.	n.r.	n.r.	n.r.	n.r.	n.r.	n.r.	n.r.	n.r.	n.r.	n.r.	n.r.	n.r.	n.r.	79.43	82.28	82.77	100	99.98
F4	n.r.	n.r.	n.r.	77.31	n.r.	n.r.	n.r.	n.r.	n.r.	n.r.	n.r.	n.r.	n.r.	n.r.	80.98	82.76	83.1	99.98	100

n.r.: ANI values for these pairs of genomes are below ~75% and therefore not reported

Table S3 Whole-genome average amino acid identity (%) values for the cable bacteria genomes. Colored cells indicate values > 65%, above the proposed genus delineation value (Konstantinidis et al., 2017).

name	AUS3	AW2	AW3_4	AU1-5	AW1	AW5	AX2	LOE1_4	AX1_4	AS4_5	TB	MAN1_	ATG1	ATG2	A1	N2	N3	US1	US2	US4	US5	AR1	AR5	AX5	MCF	GM3_4	A2	A3	A5	LOE2	EH2	AUS1_	AUS4	AR4	AR3	F1	F5	GS	F3	F4
AUS3	100	94.98	95.11	94.95	95.65	95.32	94.7	94.55	95.57	96.03	85.93	74.11	70.19	68.86	71.08	70.59	70.99	73.11	73.18	74.84	72.04	68.21	72.17	74.16	75.27	72.7	71.78	67.99	73.89	71.36	69.11	67.69	67.38	67.12	62.78	62.02	63.44	63.91	60.85	61.63
AW2	94.98	100	96.07	96.05	96.36	96.47	95.86	95.92	96.19	94.41	86.35	74.77	71.08	70.14	73.12	73.28	73.75	75.14	74.71	75.06	74.6	68.51	72.26	74.37	75.44	73.3	74.4	70.34	74.97	72.81	71.61	68.71	68.07	67.5	63.21	63.1	64.07	64.22	61.86	63.32
AW3_4	95.11	96.07	100	95.99	96.23	96.52	96.1	95.93	96.31	94.57	87.1	75.25	71.81	70.91	73.95	74.46	75.65	75.9	75.51	75.7	75.29	69.69	73.14	75.05	76.1	74.41	75.12	71.79	75.26	73.45	72.45	69.82	68.9	68.27	63.75	64.89	64.23	64.33	63.63	64.36
AU1-5	94.95	96.05	95.99	100	96.45	96.35	95.86	96.09	96.35	95.03	87.23	75.51	72.58	71.3	74.75	75.16	75.6	76.26	75.9	75.81	75.85	69.97	73.79	75.52	76.23	74.53	76.23	72.9	75.8	73.5	72.97	70.78	70.44	68.55	64.35	64.33	64.89	64.68	64.26	64.85
AW1	95.65	96.36	96.23	96.45	100	96.68	96.08	96.07	99.82	95.44	87.43	75.8	72.17	70.9	74.26	74.28	75.35	76	75.52	75.26	75.68	69.42	73.18	75.15	76.22	74.27	75.57	72.37	75.99	73.77	72.56	69.8	69.41	68.25	63.69	64.62	64.8	64.7	64.06	64.01
AW5	95.32	96.47	96.52	96.35	96.68	100	96.21	96.34	96.79	95.17	87.24	75.26	72.31	71.04	74.2	73.82	75.44	76.13	75.67	75.63	75.79	69.52	73.83	75.44	76.38	74.18	75.27	72.58	75.7	72.68	72.3	69.44	68.85	68.44	64.13	64.07	64.57	64.53	63.16	64.63
AX2	94.7	95.86	96.1	95.86	96.08	96.21	100	95.58	96.09	94.36	85.88	74.79	71.28	70.55	72.42	72.53	73.22	73.99	73.82	75.23	73.37	69.47	72.01	74.14	75.86	73.79	74.22	68.7	74.53	72.48	71.19	69.11	68.11	67.71	63.08	63.01	64.28	63.96	61.59	63.12
LOE1_4_5	94.55	95.92	95.93	96.09	96.07	96.34	95.58	100	95.96	94.71	86.4	74.4	70.57	70.03	71.88	71.54	72.74	74.13	74.51	75.25	73.04	68.19	72.69	74.36	75.75	73.32	73.58	69.26	74.87	72.42	70.84	68.57	68.17	67.53	62.96	62.74	63.96	63.97	61.29	62.64
AX1_4	95.57	96.19	96.31	96.35	99.82	96.79	96.09	95.96	100	95.3	87.35	75.92	72.5	71.14	74.42	74.87	75.94	76.05	75.89	75.65	75.77	69.97	73.41	75.15	76.4	74.57	76.04	72.69	75.97	73.85	72.59	69.75	69.35	68.37	63.75	64.7	64.87	64.72	64.36	64.83
AS4_5	96.03	94.41	94.57	95.03	95.44	95.17	94.36	94.71	95.3	100	86.59	74.48	70.83	70.35	72.57	72.34	74.08	74	74.55	75.37	72.66	68.83	72.6	74.52	75.39	73.5	73.79	70.52	74.78	72.08	70.35	68.82	68.35	67.94	63.13	63.51	64.12	64.3	62.07	63.26
TB	85.93	86.35	87.1	87.23	87.43	87.24	85.88	86.4	87.35	86.59	100	74.07	70.99	70.18	71.96	72.54	72.89	74.35	73.17	75.52	72.97	68.28	72.93	74.33	75.63	73.8	73.54	69.51	73.98	71.97	70.37	67.12	67.12	68.31	62.45	62.58	63.86	64.08	61.03	61.8
MAN1_4	74.11	74.77	75.25	75.51	75.8	75.26	74.79	74.4	75.92	74.48	74.07	100	69.29	68.19	70.84	70.01	70.45	72.88	72.86	74.34	70.72	67.47	71.23	73.3	73.73	72.17	71.63	67.7	73.01	70.8	69.2	67.83	67.49	65.46	61.84	62.05	62.51	63.58	59.87	60.54
ATG1	70.19	71.08	71.81	72.58	72.17	72.31	71.28	70.57	72.5	70.83	70.99	69.29	100	87.67	68.58	68.83	68.24	72.43	71.49	74.79	70.54	67.58	71.52	72.61	74.37	71.81	70.67	66.71	73.44	67.92	65.81	65.23	65.7	65.18	60.65	59.11	60.71	61.96	57.8	58.67
ATG2	68.86	70.14	70.91	71.3	70.9	71.04	70.55	70.03	71.14	70.35	70.18	68.19	87.67	100	69.38	70.12	69.91	72.34	71.66	74.77	71.31	67.55	71.17	71.87	73.37	71.22	71.76	68.01	73.1	67.68	65.96	64.3	65.59	64.64	60.36	59.4	59.53	60.47	57.62	57.57
A1	71.08	73.12	73.95	74.75	74.26	74.2	72.42	71.88	74.42	72.57	71.96	70.84	68.58	69.38	100	85.71	83.89	90.17	90.55	93.74	88.03	71.23	77.77	78.3	82.59	78.07	78.04	73.16	80.33	71.48	63.81	64.07	64.58	67.69	59.67	59.25	61.46	64.03	57.58	58.23
N2	70.59	73.28	74.46	75.16	74.28	73.82	72.53	71.54	74.87	72.34	72.54	70.01	68.83	70.12	85.71	100	86.92	92.76	90.75	96.46	90.43	69.78	76.52	79.31	81.99	78.49	73.77	69.17	79.79	68.98	63.64	61.72	64.2	66.9	58.86	57.56	60.62	62.47	56.27	56.85
N3	70.99	73.75	75.65	75.6	75.35	75.44	73.22	72.74	75.94	74.08	72.89	70.45	68.24	69.91	83.89	86.92	100	92.4	90.52	94.69	88.61	69.26	77	79.66	82.45	79.06	74.64	72.45	78.86	70.5	63.62	63.76	65.54	67.34	59.44	58.32	60.97	63.92	57.54	58.25
US1	73.11	75.14	75.9	76.26	76	76.13	73.99	74.13	76.05	74	74.35	72.88	72.43	72.34	90.17	92.76	92.4	100	95.62	97.82	93.97	72.64	80.34	81.16	83.62	80.43	80.4	75.68	81.61	72.38	66.52	65.49	67.13	68.13	62.77	62.07	62.65	64.88	60.01	60.9
US2	73.18	74.71	75.51	75.9	75.52	75.67	73.82	74.51	75.89	74.55	73.17	72.86	71.49	71.66	90.55	90.75	90.52	95.62	100	97.35	93.2	72.11	78.89	81.24	84.32	80.44	79.57	76.23	81.62	73.04	66.82	66.46	67.48	68.68	61.37	61.83	63.73	64.82	59.88	61.36
US4	74.84	75.06	75.7	75.81	75.26	75.63	75.23	75.25	75.65	75.37	75.52	74.34	74.79	74.77	93.74	96.46	94.69	97.82	97.35	100	96.72	77.91	81.79	82.63	84.16	80.6	82.86	79.34	85.28	76.71	71.27	70.7	71.62	70.59	64.53	64.03	64.45	64.62	62.38	64.34
US5	72.04	74.6	75.29	75.85	75.68	75.79	73.37	73.04	75.77	72.66	72.97	70.72	70.54	71.31	88.03	90.43	88.61	93.97	93.2	96.72	100	71.72	79.1	80.77	83.26	79.93	77.49	74.31	80.48	72.28	64.77	64.42	66.28	67.95	61.06	59.56	61.68	63.08	56.61	57.66
AR1	68.21	68.51	69.69	69.97	69.42	69.52	69.47	68.19	69.97	68.83	68.28	67.47	67.58	67.55	71.23	69.78	69.26	72.64	72.11	77.91	71.72	100	79.05	76.48	76.32	74.73	70.23	66.87	73.66	69.56	64.64	62.73	64.1	64.36	59.98	54.46	57.49	58.87	54.38	54.37
AR5	72.17	72.26	73.14	73.79	73.18	73.83	72.01	72.69	73.41	72.6	72.93	71.23	71.52	71.17	77.77	76.52	77	80.34	78.89	81.79	79.1	79.05	100	79.91	80.94	77.73	77.76	74.13	78.08	73.76	67.76	66.94	67.75	67.28	63.32	61.19	61.47	62.57	59.58	59.62
AX5	74.16	74.37	75.05	75.52	75.15	75.44	74.14	74.36	75.15	74.52	74.33	73.3	72.61	71.87	78.3	79.31	79.66	81.16	81.24	82.63	80.77	76.48	79.91	100	93.64	82.31	81.37	78.36	80.21	75.65	69.73	69.27	69.77	67.91	64.34	63.34	64.49	64.35	62.09	62.48
MCF	75.27	75.44	76.1	76.23	76.22	76.38	75.86	75.75	76.4	75.39	75.63	73.73	74.37	73.37	82.59	81.99	82.45	83.62	84.32	84.16	83.26	76.32	80.94	93.64	100	83.78	83.7	79.8	83.04	77.1	70.6	70.54	70.81	69.97	64.76	64.77	65.4	65.81	63.92	64.8
GM3_4	72.7	73.3	74.41	74.53	74.27	74.18	73.79	73.32	74.57	73.5	73.8	72.17	71.81	71.22	78.07	78.49	79.06	80.43	80.44	80.6	79.93	74.73	77.73	82.31	83.78	100	79.14	76.81	78.84	73.29	68.4	67.58	68.34	67.85	62.98	62.98	63.8	63.57	61.38	62.9
A2	71.78	74.4	75.12	76.23	75.57	75.27	74.22	73.58	76.04	73.79	73.54	71.63	70.67	71.76	78.04	73.77	74.64	80.4	79.57	82.86	77.49	70.23	77.76	81.37	83.7	79.14	100	88.28	90.27	73.84	67.78	67.02	66.85	68.43	60.6	59.66	61.69	64.36	57.36	59.43
A3	67.99	70.34	71.79	72.9	72.37	72.58	68.7	69.26	72.69	70.52	69.51	67.7	66.71	68.01	73.16	69.17	72.45	75.68	76.23	79.34	74.31	66.87	74.13	78.36	79.8	76.81	88.28	100	87.72	70.37	63.65	63.3	63.87	65.35	58.08	56.76	58.15	61.8	53.84	54.3
A5	73.89	74.97	75.26	75.8	75.99	75.7	74.53	74.87	75.97	74.78	73.98	73.01	73.44	73.1	80.33	79.79	78.86	81.61	81.62	85.28	80.48	73.66	78.08	80.21	83.04	78.84	90.27	87.72	100	75.75	69.35	67.77	68.44	69.03	63.49	61.68	63.97	64.41	62.32	62.6
LOE2	71.36	72.81	73.45	73.5	73.77	72.68	72.48	72.42	73.85	72.08	71.97	70.8	67.92	67.68	71.48	68.98	70.5	72.38	73.04	76.71	72.28	69.56	73.76	75.65	77.1	73.29	73.84	70.37	75.75	100	68.56	69.36	67.81	65.42	61.47	60.38	62.25	63.16	58.94	59.85
EH2	69.11	71.61	72.45	72.97	72.56	72.3	71.19	70.84	72.59	70.35	70.37	69.2	65.81	65.96	63.81	63.64	63.62	66.52	66.82	71.27	64.77	64.64	67.76	69.73	70.6	68.4	67.78	63.65	69.35	68.56	100	66.17	65.84	63.56	59.22	57.9	59.69	61.08	55.77	55.99
AUS1_2	67.69	68.71	69.82	70.78	69.8	69.44	69.11	68.57	69.75	68.82	67.12	67.83	65.23	64.3	64.07	61.72	63.76	65.49	66.46	70.7	64.42	62.73	66.94	69.27	70.54	67.58	67.02	63.3	67.77	69.36	66.17	100	69.12	62.46	58.29	57.57	60.1	61.08	54.88	56.94
AUS4	67.38	68.07	68.9	70.44	69.41	68.85	68.11	68.17	69.35	68.35	67.12	67.49	65.7	65.59	64.58	64.2	65.54	67.13	67.48	71.62	66.28	64.1	67.75	69.77	70.81	68.34	66.85	63.87	68.44	67.81	65.84	69.12	100	62.04	57.13	56.21	58.24	60.4	55.63	55.23
AR4	67.12	67.5	68.27	68.55	68.25	68.44	67.71	67.53	68.37	67.94	68.31	65.46	65.18	64.64	67.69	66.9	67.34	68.13	68.68	70.59	67.95	64.36	67.28	67.91	69.97	67.85	68.43	65.35	69.03	65.42	63.56	62.46	62.04	100	65.5	61.73	63.37	64.11	61.01	60.75
AR3	62.78	63.21	63.75	64.35	63.69	64.13	63.08	62.96	63.75	63.13	62.45	61.84	60.65	60.36	59.67	58.86	59.44	62.77	61.37	64.53	61.06	59.98	63.32	64.34	64.76	62.98	60.6	58.08	63.49	61.47	59.22	58.29	57.13	65.5	100	60.11	62.3	63.55	57.85	58.16
F1	62.02	63.1	64.89	64.33	64.62	64.07	63.01	62.74	64.7	63.51	62.58	62.05	59.11	59.4	59.25	57.56	58.32	62.07	61.83	64.03	59.56	54.46	61.19	63.34	64.77	62.98	59.66	56.76	61.68	60.38	57.9	57.57	56.21	61.73	60.11	100	89.46	91.99	69.36	69.8
F5	63.44	64.07	64.23	64.89	64.8	64.57	64.28	63.96	64.87	64.12	63.86	62.51	60.71	59.53	61.46	60.62	60.97	62.65	63.73	64.45	61.68	57.49	61.47	64.49	65.4	63.8	61.69	58.15	63.97	62.25	59.69	60.1	58.24	63.37	62.3	89.46	100	91.09	73.34	74.91
GS	63.91	64.22	64.33	64.68	64.7	64.53	63.96	63.97	64.72	64.3	64.08	63.58	61.96	60.47	64.03	62.47	63.92	64.88	64.82	64.62	63.08	58.87	62.57	64.35	65.81	63.57	64.36	61.8	64.41	63.16	61.08	61.08	60.4	64.11	63.55	91.99	91.09	100	76.33	77.43
F3	60.85	61.86	63.63	64.26	64.06	63.16	61.59	61.29	64.36	62.07	61.03	59.87	57.8	57.62	57.58	56.27	57.54	60.01	59.88	62.38	56.61	54.38	59.58	62.09	63.92	61.38	57.36	53.84	62.32	58.94	55.77	54.88	55.63	61.01	57.85	69.36	73.34	76.33	100	92.87
F4	61.63	63.32	64.36	64.85	64.01	64.63	63.12	62.64	64.83	63.26	61.8	60.54	58.67	57.57	58.23	56.85	58.25	60.9	61.36	64.34	57.66	54.37	59.62	62.48	64.8	62.9	59.43	54.3	62.6	59.85	55.99	56.94	55.23	60.75	58.16	69.8	74.91	77.43	92.87	100

Table S4 Mismatches of probe Egiga134 with 16S rRNA gene sequences of cable bacteria and other Desulfobacterota

	Egiga134
	TCCAGATGCTCGGGATAG
Candidatus Electrothrix gigas strain AU1-5 filament AU2	——————————————————
Candidatus Electrothrix gigas strain AX1_4 filament AX1	——————————————————
Candidatus Electrothrix gigas strain AX1_4 filament AX4	——————————————————
Candidatus Electrothrix gigas strain AX2	——————————————————
Candidatus Electrothrix gigas strain AW3_4 filament AW3	——————————————————
Candidatus Electrothrix gigas strain AW3_4 filament AW4	——————————————————
Candidatus Electrothrix gigas strain AW1	——————————————————
Candidatus Electrothrix gigas strain AW2	——————————————————
Candidatus Electrothrix gigas strain AW5	——————————————————
Candidatus Electrothrix gigas strain LOE1_4_5 filament LOE1	——————————————————
Candidatus Electrothrix gigas strain LOE1_4_5 filament LOE4	——————————————————
Candidatus Electrothrix gigas strain LOE1_4_5 filament LOE5	——————————————————
Candidatus Electrothrix gigas strain AU1-5 filament AU1	—————G————————————
Candidatus Electrothrix gigas strain AU1-5 filament AU3	—————G————————————
Candidatus Electrothrix gigas strain AU1-5 filament AU4	—————G————————————
Candidatus Electrothrix gigas strain AU1-5 filament AU5	—————G————————————
Candidatus Electrothrix gigas strain AS4_5 filament AS4	——T———————————————
Candidatus Electrothrix gigas strain AS4_5 filament AS5	——T———————————————
Candidatus Electrothrix gigas strain AUS3	——T———————————————
Candidatus Electrothrix sp. LOE2	————————A—————————
Candidatus Electrothrix sp. AR1	——T—————A—————————
KR912339.1 Candidatus Electrothrix communis isolate AarhusBay A1	—————G——A—————————
KR912343.1 Candidatus Electrothrix communis isolate Nitrate N2	—————G——A—————————
KR912344.1 Candidatus Electrothrix communis isolate Nitrate N3	—————G——A—————————
KR912345.1 Candidatus Electrothrix communis isolate Saltmarsh US1	—————G——A—————————
KR912346.1 Candidatus Electrothrix communis isolate Saltmarsh US2	—————G——A—————————
KR912347.1 Candidatus Electrothrix communis isolate Saltmarsh US4	—————G——A—————————
KR912348.1 Candidatus Electrothrix communis isolate Saltmarsh US5	—————G——A—————————
KR912340.1 Candidatus Electrothrix marina isolate AarhusBay A2	—————G——A—————————
KR912341.1 Candidatus Electrothrix marina isolate AarhusBay A3	—————G——A—————————
KR912342.1 Candidatus Electrothrix marina isolate AarhusBay A5	—————G——A—————————
KR912349.1 Candidatus Electrothrix japonica isolate TokyoBay TB	—————G——T—————————
Desulfobulbaceae cable bacterium filament AR4	—————G——A—————————
Candidatus Electrothrix sp. ATG1_2 filament ATG1	—————G——A—————————
Candidatus Electrothrix sp. ATG1_2 filament ATG2	—————G——A—————————
Desulfobulbaceae cable bacterium filament AR3	C———————A——————C——
Candidatus Electrothrix sp. AUS4	—————G——T—T———————
Candidatus Electrothrix sp. MAN1_4 filament MAN1	————AG——A—————————
Candidatus Electrothrix sp. MAN1_4 filament MAN4	————AG——A—————————
Candidatus Electronema GS 2729875368-Ga0183576 12823	———T————AC———A—C——
KP728463.1 Candidatus Electronema palustre isolate Freshwater Gib-F3	——————C—AC———A—C——
KP728464.1 Candidatus Electronema palustre isolate Freshwater Gib-F4	——————C—AC———A—C——
Candidatus Electrothrix sp. AUS1_2 filament AUS1	———G—GC—A——————C——
Candidatus Electrothrix sp. EH2	———G—G——A————A—C——
KR912338.1 Candidatus Electrothrix aarhusiensis isolate AarhusBay MCF4-14	————AGC—A—T———————
Candidatus Electrothrix aarhusiensis filament AX5	————AGC—A—T———————
Candidatus Electrothrix sp. GM3_4 filament GM3	————AGC—A—T———————
Candidatus Electrothrix sp. GM3_4 filament GM4	————AGC—A—T———————
KP728462.1 Candidatus Electronema nielsenii isolate Freshwater Gib-F1	———TA———AC———A—C——
KP728465.1 Candidatus Electronema nielsenii isolate Freshwater Gib-F5	———TA———AC———A—C——
Candidatus Electrothrix sp. AR5	————AGC—ACT———————
NR_040977.1 Desulfobulbus japonicus strain Pro1	C—TCC———T—T——————A
NR_074331.1 Desulfotalea psychrophila LSv54 16S ribosomal RNA partial sequence	C——T———ATCT——————A
NR_025150.1 Desulfobulbus mediterraneus strain 86FS1	——TTT———TCT——————A
U12253.1 Desulfobulbus rhabdoformis 16S	——TT————TCT——A———A
NR_074446.1 Desulfovibrio vulgaris strain Hildenborough	C———TGA—T—G——A———A
NR_028895.1 Desulfobacterium catecholicum strain NZva20 16S ribosomal RNA partial sequence	C——T—G—ATCT——————A
NR_074930.1 Desulfobulbus propionicus DSM 2032	——TTC———TCT——A———A
NR_029305.1 Desulfobulbus elongatus strain FP	——TTC———TCT——A———A
NR_117882.1 Desulfobulbus alkaliphilus strain APS1	——ATAT——TCT——————A
NR_074971.1 Desulfurivibrio alkaliphilus strain AHT2	C——TTC—ATCT——————A
NR_115066.1 Desulfopila inferna strain JS_SRB250Lac	C——T—G—ATCT——A———A
NR_026354.1 Desulfofustis glycolicus strain PerGlyS 16S ribosomal RNA partial sequence	C——TTGCAT————A———A
NR_102510.1 Desulfocapsa sulfexigens strain DSM 10523	C——TCGCATCA——————A
KY418001.1 Uncultured delta proteobacterium clone Seq7 16SrRNA gene partial sequence	C—TCTGCATCT——————A
NR_028742.1 Desulforhopalus singaporensis strain Spore T1	C——CTTCATCT——A———A

Table S5 129 genes specific to Ca. Electrothrix gigas and results of blastp analysis of translated protein sequences vs the cable bacteria genomes and vs NCBI-nr

Q-locus tag	Q-annotation	Q-length	SignalP 6.0 slov predicted SP	TMHMM 2.0 predicted helices	Blastp vs Cable bacteria genomes except Candidatus Electrothrix gigas, best hit S-name	S-locus tag	S-annotation	S-length	Q-start	Q-end	S-start	S-end	Aligned length	%Al length	% identity	% positives	Bitscore	E-value	
AW1 00002	hypothetical protein	56			Candidatus Electrothrix sp. LOE2	LOE2 01267	hypothetical protein	129	1	56	58	113	56	100	67.857	82.14	82.8	1.87E-22	
AW1_00018	PqqA peptide cyclase	301			Candidatus Electrothrix sp. GM3_4	GM3_4_03381	PqqA peptide cyclase	493	24	183	2	173	172	57.14	27.907	48.26	75.1	5.54E-15	
AW1 00022	IS1595 family transposase ISCco3	320			Desulfobulbaceae cable bacterium AR3	AR3 01832	Modification methylase BspRI	240	1	233	1	231	234	73.13	72.65	85.47	355	6.58E-124	
AW1 00042	hypothetical protein	69			Candidatus Electrothrix communis isolate US1	US1 00809	hypothetical protein	157	1	69	1	66	69	100.00	79.71	88.41	114	3.51E-34	
AW1 00045	hypothetical protein	228			Candidatus Electrothrix communis isolate US4	US4 01126	hypothetical protein	235	2	224	9	230	227	99.56	59.912	71.37	253	3.37E-85	
AW1 00050	hypothetical protein	246		7	Candidatus Electrothrix sp. AR5	AR5 00621	hypothetical protein	262	2	242	9	262	255	103.66	63.922	76.08	289	7.09E-99	
AW1 00052	hypothetical protein	216			Candidatus Electrothrix sp. ATG1 2 filament ATG2	ATG2 01025	hypothetical protein	244	1	211	40	236	211	97.69	60.664	74.88	254	1.01E-85	
AW1 00059	hypothetical protein	248		2	Candidatus Electrothrix marina isolate A5	A5 00456	hypothetical protein	302	1	224	1	228	228	91.94	64.912	73.68	273	4.78E-92	
AW1 00079	hypothetical protein	195			Candidatus Electronema aureum isolate GS	GS 01850	Internalin A	353	8	164	12	168	157	80.51	45.223	59.24	107	4.81E-28	
AW1 00084	hypothetical protein	465		12	Candidatus Electrothrix japonica isolate TB	TB 01216	hypothetical protein	331	1	323	1	324	324	69.68	94.753	97.53	629	0	
AW1 00183	IS1595 family transposase ISCco3	325			Candidatus Electrothrix sp. EH2	EH2 01102	DNA-cytosine methyltransferase	413	3	301	69	402	341	104.92	45.455	57.18	242	3.89E-77	
AW1 00201	hypothetical protein	82			Candidatus Electrothrix sp. MAN1 4	MAN1 4 03003	hypothetical protein	107	31	60	59	88	30	36.59	43.333	53.33	26.2	2.2	
AW1 00235	hypothetical protein	2265	SP	1	Candidatus Electrothrix sp. MAN1 4	MAN1 4 00631	hypothetical protein	1690	614	2265	14	1690	1724	76.11	65.545	75.35	2155	0	
AW1 00243	Adaptive-response sensory-kinase	505			Candidatus Electrothrix aarhusiensis filament AX5	AX5 00141	Adaptive-response sensory-kinase SasA	536	3	502	21	527	516	102.18	63.76	79.46	663	0	
AW1 00267	hypothetical protein	138	LIPO	1	Candidatus Electrothrix communis isolate US4	US4 01084	hypothetical protein	114	24	138	1	114	115	83.33	70.435	83.48	169	1.52E-55	
AW1 00319	hypothetical protein	175			Candidatus Electrothrix sp. AUS4	AUS4 00203	GTP pyrophosphokinase	165	8	166	2	160	159	90.86	72.956	88.05	254	1.34E-87	
AW1 00321	hypothetical protein	435		2	Candidatus Electrothrix aarhusiensis filament AX5	AX5 00675	hypothetical protein	438	14	434	11	436	428	98.39	55.607	71.73	487	3.91E-171	
AW1 00322	hypothetical protein	160			Candidatus Electrothrix sp. AUS1 2	AUS1 2 01468	hypothetical protein	159	7	160	7	159	154	96.25	60.39	83.12	201	5.28E-67	
AW1 00370	hypothetical protein	702		4	Candidatus Electrothrix communis isolate US4	US4 01051	hypothetical protein	698	43	697	1	691	691	98.43	68.307	79.31	942	0	
AW1 00378	hypothetical protein	646			Candidatus Electrothrix communis isolate US4	US4 02356	hypothetical protein	796	1	646	145	796	659	102.01	60.546	76.18	766	0	
AW1 00385	hypothetical protein	63		1	Candidatus Electrothrix sp. GM3 4	GM3 4 01838	hypothetical protein	66	5	62	1	58	58	92.06	39.655	62.07	55.1	3.46E-12	
AW1 00389	hypothetical protein	358			Candidatus Electrothrix sp. EH2	EH2 03011	hypothetical protein	119	237	352	1	116	116	32.40	74.138	86.21	185	1.74E-58	partial
AW1 00390	hypothetical protein	117			Candidatus Electrothrix aarhusiensis isolate MCF	MCF 01931	hypothetical protein	804	1	86	431	524	94	80.34	27.66	44.68	33.1	0.036	
AW1 00402	Protein with DUF697	165		2	Candidatus Electrothrix sp. EH2	EH2 01963	Protein with DUF697	164	9	165	5	164	160	96.97	58.125	73.75	191	6.27E-63	
AW1 00476	hypothetical protein	213	LIPO	1	Candidatus Electrothrix aarhusiensis filament AX5	AX5 03460	hypothetical protein	191	10	212	1	191	210	98.59	53.333	67.62	201	9.15E-66	
AW1 00489	hypothetical protein	43			Candidatus Electrothrix aarhusiensis isolate MCF	MCF 02571	hypothetical protein	41	1	33	1	33	33	76.74	54.545	81.82	42.7	8.14E-08	
AW1 00530	hypothetical protein	72		1	Candidatus Electrothrix sp. ATG1 2 filament ATG2	ATG2 00337	hypothetical protein	533	27	72	372	414	46	63.89	36.957	54.35	29.6	0.15	
AW1 00613	hypothetical protein	192		3	Desulfobulbaceae cable bacterium AR3	AR3 03065	hypothetical protein	147	59	191	1	135	137	71.35	54.015	70.07	150	7.37E-47	
AW1 00630	Undecaprenyl-phosphate 4-deoxy	1070			Candidatus Electrothrix japonica isolate TB	TB 00161	hypothetical protein	625	467	1070	1	625	628	58.69	82.325	90.29	1091	0	partial
AW1 00720	hypothetical protein	326			Candidatus Electrothrix aarhusiensis isolate MCF	MCF 02261	hypothetical protein	141	1	127	1	128	130	39.88	59.231	78.46	131	1.56E-37	partial
AW1 00721	hypothetical protein	43		1	Candidatus Electronema aureum isolate GS	GS 01193	Protein-glutamate methylesterase/protein-gluta	385	6	20	55	69	15	34.88	66.667	73.33	25	1.8	
AW1 00722	hypothetical protein	256		2	Candidatus Electrothrix sp. LOE2	LOE2 03103	hypothetical protein	200	18	256	1	200	239	93.36	58.996	70.29	265	3.66E-90	
AW1 00828	hypothetical protein	278			Candidatus Electrothrix sp. ATG1 2 filament ATG2	ATG2 03287	hypothetical protein	271	10	275	4	269	266	95.68	63.534	75.56	355	3.81E-124	
AW1 00872	Endolytic peptidoglycan translyc	385	LIPO	1	Candidatus Electrothrix aarhusiensis isolate MCF	MCF 00212	Endolytic peptidoglycan transglycosylase RlpA	383	109	381	107	366	273	70.91	68.864	79.49	373	4.43E-128	
AW1 00881	hypothetical protein	123	SP	1	Candidatus Electrothrix sp. LOE2	LOE2 00615	hypothetical protein	122	1	122	1	121	122	99.19	72.131	81.15	177	9.19E-59	
AW1 00938	NH(3)-dependent NAD(+) synthet	273			Candidatus Electrothrix sp. GM3 4	GM3 4 01056	Glutamine-dependent NAD(+) synthetase	586	1	256	301	532	264	96.70	25.379	44.32	65.1	9.35E-12	
AW1 00941	Quinolinate synthase A	303			Candidatus Electrothrix japonica isolate TB	TB 01632	Quinolinate synthase A	336	13	302	39	330	293	96.70	41.297	61.43	234	2.44E-75	
AW1 00999	hypothetical protein	262			Candidatus Electrothrix communis isolate US5	US5 00343	hypothetical protein	243	6	262	6	243	263	100.38	47.148	62.74	208	3.67E-67	
AW1 01006	hypothetical protein	180			Candidatus Electrothrix communis isolate US4	US4 00927	hypothetical protein	178	1	179	1	176	180	100.00	66.667	80	236	1.41E-80	
AW1 01054	hypothetical protein	301			Candidatus Electrothrix aarhusiensis filament AX5	AX5 01997	hypothetical protein	326	1	296	1	323	326	108.31	51.84	62.88	300	1.33E-101	
AW1 01056	Virulence sensor protein BvgS	1278		3	Candidatus Electrothrix communis isolate US4	US4 02176	Virulence sensor protein BvgS	1253	22	1273	3	1244	1257	98.36	67.303	81.38	1709	0	
AW1 01066	hypothetical protein	544			Candidatus Electrothrix japonica isolate TB	TB 01141	hypothetical protein	568	1	544	1	568	579	106.43	56.822	67.7	612	0	
AW1 01076	hypothetical protein	360		4	Candidatus Electrothrix japonica isolate TB	TB 01591	hypothetical protein	206	160	360	1	202	202	56.11	90.099	94.55	364	1.78E-127	partial
AW1 01152	hypothetical protein	90			Candidatus Electrothrix sp. GM3 4	GM3 4 00924	hypothetical protein	159	24	85	11	69	62	68.89	27.419	48.39	25.8	4.4	
AW1 01194	hypothetical protein	382		2	Candidatus Electrothrix sp. MAN1 4	MAN1 4 00088	hypothetical protein	354	1	381	1	353	387	101.31	62.016	75.19	459	1.74E-162	
AW1 01249	hypothetical protein	33			Candidatus Electrothrix sp. LOE2	LOE2 01161	hypothetical protein	223	5	18	3	16	14	42.42	64.286	71.43	24.6	1.6	
AW1 01277	ATP synthase subunit b	244	SP	2	Candidatus Electrothrix japonica isolate TB	TB 02088	ATP synthase subunit b, sodium ion specific	163	82	244	1	163	163	66.80	92.025	96.93	296	4.49E-103	partial
AW1 01290	hypothetical protein	183		2	Candidatus Electrothrix sp. AR5	AR5 00867	hypothetical protein	208	5	111	29	138	114	62.30	24.561	42.98	33.9	0.04	
AW1 01299	hypothetical protein	279		1	Candidatus Electrothrix aarhusiensis filament AX5	AX5 03465	Outer membrane protein OmpV	269	27	279	16	269	254	91.04	60.63	75.2	327	4.40E-113	
AW1 01316	Flavin-dependent thymidylate syr	605			Candidatus Electrothrix communis isolate US4	US4 01673	Flavin-dependent thymidylate synthase	310	1	198	1	196	198	32.73	81.313	89.39	339	9.12E-113	partial
AW1 01321	hypothetical protein	175			Desulfobulbaceae cable bacterium AR4	AR4 02929	hypothetical protein	190	3	150	25	172	148	84.57	68.919	83.11	217	1.30E-72	
AW1 01381	hypothetical protein	410	SP	1	Candidatus Electrothrix sp. GM3 4	GM3 4 00931	hypothetical protein	406	1	410	1	406	410	100.00	64.634	77.32	552	0	
AW1 01449	hypothetical protein	198			Candidatus Electrothrix japonica isolate TB	TB 02225	hypothetical protein	92	77	163	1	90	90	45.45	44.444	57.78	65.1	5.41E-14	partial
AW1 01450	hypothetical protein	222			Candidatus Electrothrix japonica isolate TB	TB 01327	hypothetical protein	98	1	98	1	97	98	44.14	65.306	75.51	124	1.24E-36	partial
AW1 01479	hypothetical protein	302	SP	4	Candidatus Electrothrix sp. MAN1 4	MAN1 4 00402	hypothetical protein	308	1	300	8	307	303	100.33	61.056	74.92	391	1.40E-137	
AW1 01499	hypothetical protein	43			Candidatus Electrothrix sp. AUS4	AUS4 02092	hypothetical protein	177	1	33	1	34	34	79.07	58.824	79.41	42.7	7.41E-07	
AW1_01514	hypothetical protein	249			Desulfobulbaceae cable bacterium AR3	AR3_02007	hypothetical protein	174	127	149	150	172	23	9.24	56.522	78.26	33.9	0.058	
AW1 01641	hypothetical protein	235		1	Candidatus Electrothrix japonica isolate TB	TB 00039	hypothetical protein	219	31	229	1	217	226	96.17	51.77	63.27	174	1.74E-54	
AW1 01678	hypothetical protein	922	SP	1	Candidatus Electrothrix communis isolate US2	US2 00331	hypothetical protein	925	1	694	1	740	747	81.02	61.446	72.42	816	0	
AW1 01694	hypothetical protein	29			Candidatus Electrothrix communis isolate US2	US2 00076	hypothetical protein	29	1	28	1	28	28	96.55	39.286	57.14	21.6	7.4	
AW1 01701	hypothetical protein	38		1	Candidatus Electrothrix sp. ATG1 2 filament ATG2	ATG2 04553	hypothetical protein	65	1	38	1	32	38	100.00	42.105	68.42	24.6	1.1	
AW1 01702	hypothetical protein	359	SP	1	Candidatus Electrothrix sp. AR1	AR1 00864	hypothetical protein	352	1	354	1	352	360	100.28	45	64.44	254	3.50E-82	
AW1 01735	hypothetical protein	343	SP		Candidatus Electrothrix aarhusiensis isolate MCF	MCF 00036	hypothetical protein	359	169	343	190	359	176	51.31	67.045	74.43	231	1.53E-73	partial
AW1 01805	hypothetical protein	324			Candidatus Electrothrix aarhusiensis isolate MCF	MCF 02955	hypothetical protein	353	5	322	33	351	321	99.07	70.405	79.44	447	7.03E-159	
AW1 01806	hypothetical protein	113			Candidatus Electrothrix japonica isolate TB	TB 00752	hypothetical protein	90	1	77	1	84	84	74.34	79.762	79.76	135	1.04E-42	
AW1 01833	hypothetical protein	429		1	Candidatus Electrothrix sp. AR5	AR5 03143	hypothetical protein	432	1	427	1	430	456	106.29	48.684	62.28	348	8.55E-117	
AW1 01838	hypothetical protein	142	SP	1	Candidatus Electrothrix sp. GM3 4	GM3 4 03558	hypothetical protein	182	1	140	1	124	140	98.59	62.857	72.86	163	4.97E-52	
AW1 01886	hypothetical protein	212		4	Candidatus Electrothrix marina isolate A5	A5 01291	hypothetical protein	208	1	203	1	203	203	95.75	64.039	78.82	279	3.20E-96	
AW1 01911	hypothetical protein	116		4	Candidatus Electrothrix communis isolate US4	US4 01291	Sialic acid TRAP transporter permease protein Si	443	23	105	25	114	90	77.59	28.889	47.78	33.9	0.019	
AW1 01912	hypothetical protein	123			Candidatus Electronema aureum isolate GS	GS 01563	hypothetical protein	365	33	108	43	121	80	65.04	32.5	52.5	35.4	0.006	
AW1 01926	hypothetical protein	234	SP		Candidatus Electrothrix aarhusiensis filament AX5	AX5 00922	hypothetical protein	225	1	231	1	217	238	101.71	44.538	57.14	146	1.31E-43	
AW1 01952	hypothetical protein	115			Candidatus Electrothrix japonica isolate TB	TB 01941	hypothetical protein	65	51	115	1	65	65	56.52	92.308	93.85	122	4.82E-38	partial
AW1 01980	hypothetical protein	87	SP		Candidatus Electrothrix sp. ATG1 2 filament ATG2	ATG2 02107	hypothetical protein	94	1	86	1	85	86	98.85	63.953	76.74	110	2.40E-33	
AW1 01981	hypothetical protein	58			Candidatus Electrothrix sp. ATG1 2 filament ATG1	ATG1 00676	hypothetical protein	202	1	57	1	57	57	98.28	77.193	84.21	89	4.15E-24	
AW1 01985	hypothetical protein	155			Candidatus Electrothrix sp. AUS1 2	AUS1 2 00851	hypothetical protein	152	3	149	4	150	147	94.84	56.463	77.55	174	1.28E-56	
AW1 02045	hypothetical protein	69	LIPO	1	Desulfobulbaceae cable bacterium AR4	AR4 01558	hypothetical protein	75	1	69	1	73	75	108.70	44	57.33	46.6	1.58E-08	
AW1 02061	hypothetical protein	49			Candidatus Electrothrix sp. MAN1 4	MAN1 4 00573	Glycerol-3-phosphate acyltransferase	210	1	13	77	89	13	26.53	69.231	92.31	24.6	3.4	
AW1_02104	hypothetical protein	309		1	Candidatus Electrothrix sp. AR5	AR5_03038	Cytochrome c-552	119	55	171	1	117	117	37.86	85.47	95.73	201	1.39E-65	
AW1 02127	hypothetical protein	187	SP	4	Candidatus Electrothrix sp. EH2	EH2 02967	hypothetical protein	203	1	187	1	203	203	108.56	61.084	73.89	236	5.51E-80	
AW1 02179	hypothetical protein	167	SP	1	Candidatus Electrothrix sp. MAN1 4	MAN1 4 01367	hypothetical protein	166	1	167	1	166	168	100.60	60.119	75	193	6.72E-64	
AW1 02199	Na(+)/H(+) antiporter subunit B	147		4	Candidatus Electrothrix sp. ATG1 2 filament ATG1	ATG1 01387	Na(+)/H(+) antiporter subunit B	89	1	88	1	88	88	59.86	96.591	97.73	158	2.05E-51	partial
AW1 02239	hypothetical protein	166		1	Candidatus Electrothrix sp. GM3 4	GM3 4 03533	hypothetical protein	163	1	159	1	157	166	100.00	54.819	69.28	171	5.97E-55	
AW1 02251	hypothetical protein	563		4	Candidatus Electrothrix sp. GM3 4	GM3 4 01176	hypothetical protein	562	1	563	1	562	581	103.20	63.855	76.59	734	0	
AW1 02256	hypothetical protein	65			Desulfobulbaceae cable bacterium AR3	AR3 02393	hypothetical protein	70	1	61	11	70	61	93.85	32.787	45.9	24.6	4.2	
AW1 02264	hypothetical protein	195	SP		Candidatus Electrothrix sp. AR5	AR5 00979	hypothetical protein	266	68	121	216	266	55	28.21	34.545	49.09	31.2	0.49	
AW1 02265	hypothetical protein	210	SP		Candidatus Electrothrix communis isolate US4	US4 00011	hypothetical protein	70	98	150	11	63	53	25.24	32.075	47.17	33.1	0.019	
AW1 02272	hypothetical protein	210		1	Candidatus Electrothrix sp. AUS1 2	AUS1 2 01310	hypothetical protein	305	1	210	64	305	242	115.24	57.438	68.6	262	2.28E-88	
AW1 02300	hypothetical protein	83		1	Candidatus Electronema aureum isolate GS	GS 00148	Chaperone protein DnaJ	401	59	74	149	164	16	19.28	50	87.5	26.6	2.7	
AW1 02322	Chaperone protein DnaJ	446		1	Candidatus Electrothrix japonica isolate TB	TB 00518	Chaperone protein DnaJ	349	1	265	92	349	267	59.87	77.154	82.4	385	2.31E-132	partial
AW1 02330	hypothetical protein	294			Candidatus Electrothrix sp. EH2	EH2 02287	hypothetical protein	294	1	294	1	294	294	100.00	63.946	81.97	417	2.82E-148	
AW1_02336	hypothetical protein	97			Candidatus Electrothrix sp. MAN1_4	MAN1_4_01903	Manganese transport system membrane proteir	218	35	68	179	212	34	35.05	29.412	50	26.2	4.8	
AW1 02350	hypothetical protein	176		2	Candidatus Electrothrix aarhusiensis filament AX5	AX5 00866	hypothetical protein	176	1	175	1	175	175	99.43	64	81.71	245	5.57E-84	
AW1 02351	hypothetical protein	205			Candidatus Electrothrix japonica isolate TB	TB 02225	hypothetical protein	92	83	170	1	92	92	44.88	48.913	60.87	79.3	2.32E-19	
AW1 02353	hypothetical protein	408	LIPO	1	Candidatus Electrothrix communis isolate US5	US5 00660	hypothetical protein	439	49	401	22	362	354	86.76	57.91	74.58	428	2.44E-148	
AW1 02372	hypothetical protein	182			Candidatus Electrothrix sp. EH2	EH2 02642	hypothetical protein	183	3	182	2	183	182	100.00	62.088	80.77	244	1.72E-83	
AW1 02386	hypothetical protein	402		1	Candidatus Electrothrix sp. ATG1 2 filament ATG2	ATG2 03078	hypothetical protein	366	107	388	1	250	284	70.65	48.592	65.49	241	2.29E-76	
AW1 02418	hypothetical protein	106	SP		Candidatus Electrothrix sp. GM3 4	GM3 4 02899	hypothetical protein	97	6	87	10	97	91	85.85	34.066	53.85	51.6	8.10E-10	
AW1 02446	Fimbrial protein	165	PILIN	1	Candidatus Electrothrix sp. AUS1 2	AUS1 2 02108	hypothetical protein	130	10	129	1	118	120	72.73	47.5	65.83	111	6.22E-32	
AW1_02447	hypothetical protein	167	PILIN	1	Candidatus Electrothrix sp. AUS1_2	AUS1_2_02108	hypothetical protein	130	11	135	3	122	125	74.85	38.4	57.6	95.5	8.10E-10	
AW1_02449	Fimbrial protein	182	PILIN	1	Candidatus Electrothrix sp. ATG1_2 filament ATG1	ATG1_00044	Fimbrial protein	161	12	180	2	160	169	92.86	28.994	51.48	81.3	1.28E-19	
AW1 02487	hypothetical protein	64			Desulfobulbaceae cable bacterium AR4	AR4 02240	hypothetical protein	205	1	62	1	63	63	98.44	87.302	90.48	112	3.58E-33	
AW1_02509	Inner membrane protein YiaA	165		4	Desulfobulbaceae cable bacterium AR3	AR3_01362	hypothetical protein	243	16	77	146	208	63	38.18	31.746	52.38	34.3	0.03	
AW1 02594	hypothetical protein	90	SP		Candidatus Electrothrix sp. AUS4	AUS4 02391	hypothetical protein	94	1	63	1	63	64	71.11	64.062	78.12	75.1	3.01E-19	
AW1 02623	hypothetical protein	29			Candidatus Electrothrix sp. GM3 4	GM3 4 00270	hypothetical protein	242	1	29	1	26	29	100.00	34.483	58.62	24.6	1.5	
AW1 02648	hypothetical protein	209	LIPO	1	Candidatus Electrothrix sp. MAN1 4	MAN1 4 00784	hypothetical protein	188	1	188	1	145	188	89.95	53.723	63.3	187	2.38E-60	
AW1 02649	hypothetical protein	111	LIPO	1	Candidatus Electrothrix japonica isolate TB	TB 01585	hypothetical protein	212	1	97	84	183	100	90.09	88	91	183	4.01E-60	
AW1 02658	Actin-like protein MamK	335			Candidatus Electrothrix sp. EH2	EH2 01191	Cell shape-determining protein MreB	345	7	291	16	304	302	90.15	22.185	43.71	37	0.014	
		335			Candidatus Electrothrix sp. MAN1 4	MCI5150067.1*	hypothetical protein	192	1	188	1	188	188	56.12	84.043	94.15	328	4.65E-114	partial
AW1 02659	hypothetical protein	126			Candidatus Electrothrix sp. MAN1 4	MAN1 4 02180/I	hypothetical protein	125	1	126	1	125	132	104.76	54.545	67.42	115	4.80E-34	
AW1_02660	hypothetical protein	425			Candidatus Electronema aureum isolate GS	GS_01168	hypothetical protein	394	80	402	29	387	377	88.71	33.156	50.4	142	2.70E-38	
AW1 02672	hypothetical protein	109			Candidatus Electrothrix aarhusiensis filament AX5	AX5 01448	hypothetical protein	78	1	54	19	71	54	49.54	62.963	72.22	53.5	1.01E-10	partial
AW1 02679	hypothetical protein	231		4	Candidatus Electrothrix japonica isolate TB	TB 02100	hypothetical protein	103	88	185	5	102	98	42.42	86.735	94.9	172	3.37E-55	partial
AW1 02680	hypothetical protein	225		3	Candidatus Electrothrix sp. LOE2	LOE2 03277	hypothetical protein	218	2	220	1	217	219	97.33	49.772	64.84	224	4.05E-74	
AW1 02705	hypothetical protein	176	LIPO	1	Candidatus Electrothrix sp. MAN1 4	MAN1 4 03290	hypothetical protein	101	22	109	1	100	104	59.09	64.423	66.35	94	2.76E-25	partial
AW1 02719	Vitamin B12 transporter BtuB	781	SP	1	Candidatus Electrothrix sp. EH2	EH2 01147	Vitamin B12 transporter BtuB	513	72	584	1	513	513	65.69	92.203	96.88	966	0	partial
AW1 02753	hypothetical protein	843		1	Candidatus Electrothrix sp. MAN1 4	MAN1 4 00310	hypothetical protein	803	81	841	36	803	806	95.61	62.407	73.33	930	0	
AW1 02775	hypothetical protein	452	SP		Candidatus Electrothrix sp. MAN1 4	MAN1 4 01499	hypothetical protein	343	21	265	5	257	255	56.42	56.471	70.2	272	4.68E-88	partial
AW1 02825	hypothetical protein	341		1	Candidatus Electrothrix sp. AR5	AR5 03174	hypothetical protein	347	1	328	1	334	345	101.17	58.841	70.72	365	2.94E-126	
AW1 02828	hypothetical protein	702		4	Candidatus Electrothrix japonica isolate TB	TB 01241	hypothetical protein	525	1	525	1	521	529	75.36	88.847	93.57	953	0	
AW1 02845	Adenine deaminase	590			Candidatus Electrothrix marina isolate A3	A3 00046	Adenine deaminase	604	18	583	24	588	583	98.81	42.882	58.15	424	4.69E-142	
AW1 02849	hypothetical protein	282			Candidatus Electrothrix sp. ATG1 2 filament ATG1	ATG1 00796	hypothetical protein	268	22	272	12	256	251	89.01	70.916	83.27	362	5.11E-127	
AW1 02950	hypothetical protein	96			Candidatus Electrothrix japonica isolate TB	TB 01614	hypothetical protein	175	10	96	15	101	87	90.63	87.356	93.1	154	1.25E-49	
AW1 02964	hypothetical protein	66	SP		Candidatus Electrothrix sp. AUS1 2	AUS1 2 00485	hypothetical protein	50	18	52	10	40	35	53.03	31.429	57.14	24.6	3.1	
AW1 03034	hypothetical protein	154		3	Candidatus Electrothrix japonica isolate TB	TB 02166	hypothetical protein	297	1	153	136	288	153	99.35	90.196	94.12	285	3.25E-98	
AW1 03134	hypothetical protein	179			Candidatus Electrothrix communis isolate US2	US2 01161	hypothetical protein	148	28	169	1	142	142	79.33	80.282	91.55	245	1.68E-84	
AW1_03167	hypothetical protein	132			Candidatus Electrothrix sp. AUS1_2	AUS1_2_00947	UDP-3-O-acyl-N-acetylglucosamine deacetylase	333	81	132	247	300	54	40.91	29.63	50	30.8	0.25	
AU1-5 00075	hypothetical protein	48		1	Candidatus Electrothrix aarhusiensis filament AX5	AX5 02208	hypothetical protein	306	28	47	284	303	20	41.67	70	80	28.1	0.22	
AU1-5 00862	hypothetical protein	264			Candidatus Electronema palustris isolate F3	F3 00434	hypothetical protein	286	2	256	35	283	257	97.35	48.638	64.98	228	1.97E-74	
AU1-5 01232	hypothetical protein	35			Candidatus Electrothrix sp. EH2	EH2 00860	hypothetical protein	215	1	28	1	28	28	80.00	96.429	100	62.4	2.87E-14	
AU1-5 02388	Sec-independent protein transloc	278		5	Candidatus Electrothrix sp. ATG1 2 filament ATG1	ATG1 02810	Sec-independent protein translocase protein Tat	210	68	276	1	209	209	75.18	74.641	84.69	315	1.49E-109	

Q-locus tag	CD-SEARCH conserved domains	InterProScan	eggNOG-mapper v2.1	Blastp vs NCBI-nr database [January 2022][%], threshold cutoff: -evalue 0.05, best hit, omitting cable bacteria S-name	S-locus tag	S-annotation	S-length	Q-start	Q-end	S-start	S-end	Aligned length	%Al.length	% identity	% positive	Bitscore	E-value
AW1_00018	pfam04055 Radical SAM superfamily protein	IPR013785, Aldolase-type TIM barrel	Iron-sulfur cluster-binding domain containing protein	Deltaproteobacteria bacterium	tpg|HGM98119.1|	radical SAM protein	300	8	279	8	280	274	91.03	51.825	68.61	301	2.72E-98
AW1 00201	no sign. hit	-	-														
AW1 00390	no sign. hit	-	-														
AW1 00530	no sign. hit	-	-														
AW1 00721	no sign. hit	-	-														
AW1 00938	cd00553 NAD(+)-synthase	IPR003694, NAD(+) synthetase	NH(3)-dependent NAD(+) synthetase	Bacteroidetes bacterium GWF2	gb|OFY18560.1|	NAD(+) synthase	266	7	271	1	265	265	97.07	62.264	76.6	347	#######
AW1 01152	no sign. hit	-	-														
AW1 01249	no sign. hit	-	-														
AW1 01290	no CD	-	-	Aureivirga marina	ref|WP 196895818.	hypothetical protein	178	5	172	2	172	171	93.44	53.216	67.25	175	2.65E-52
AW1_01514	pfam13240 Zinc-ribbon domain (single)	IPR029024, TerB-like; IPR026870, Zinc-ribbon domain	-	Thiotrichales bacterium HS_08	emb|SEH04567.1|	Uncharacterised protein	426	1	246	1	246	246	98.80	62.195	76.83	319	#######
AW1 01694	no sign. hit	-	-														
AW1 01701	no sign. hit	-	-														
AW1 01911	no sign. hit	-	-														
AW1 01912	no sign. hit	-	-														
AW1 02061	no sign. hit	-	-														
AW1_02104	No CD, 1 CxxCH heme binding domain	IPR036909, Cytochrome c-like domain superfamily	-	Bacillus	ref|WP 023441086.	MULTISPECIES: DNA translocase	1318	170	305	496	632	137	44.34	49.635	63.5	103	3.79E-20
AW1 02256	no sign. hit	-	-														
AW1 02264	No CD	-	-	Chromatiaceae bacterium	gb|MCC7278879.1|	hypothetical protein	439	32	186	278	438	165	84.62	38.788	54.55	116	9.36E-27
AW1 02265	No CD	-	-	Chromatiaceae bacterium	gb|MCC7278879.1|	hypothetical protein	439	35	192	283	438	168	80.00	35.714	51.79	92	1.26E-17
AW1 02300	no sign. hit	-	-														
AW1_02336	No CD	-	Nucleic-acid-binding protein containing Zn-ribbon domain	Persicirhabdus sediminis	ref|WP 200311904.	hypothetical protein	93	1	97	1	93	97	100.00	47.423	65.98	101	1.09E-25
AW1 02351	No CD	-	-	Nostoc edaphicum	ref|WP 181929776.	hypothetical protein	200	1	203	1	198	210	102.44	38.095	55.24	120	5.30E-30
AW1 02418	no sign. hit	-	-														
AW1_02447	PRK10574 putative major pilin subunit	IPR012902, Prokaryotic N-terminal methylation site	-	Pseudoalteromonas	ref|WP 193372024.	MULTISPECIES: pilin	162	13	167	6	160	156	93.41	37.821	64.1	127	1.09E-33
AW1_02449	COG2165 Type II secretory pathway, pseudopilin PulG	IPR045584, Pilin-like	-	Xanthomonadaceae bacterium	gb|MBN8741853.1|	pilin	163	17	181	10	163	165	90.66	33.939	61.21	109	1.23E-26
AW1_02509	COG4682 Uncharacterized membrane protein YiaA	IPR038972, Inner membrane protein YiaA-like	YiaAB two helix	Azoarcus sp. TTM-91	ref|WP 169149768.	YiaA/YiaB family protein	146	5	132	3	130	128	77.58	70.312	89.06	202	1.37E-63
AW1 02623	no sign. hit	-	-														
AW1_02660	pfam14312 FG-GAP repeat	IPR011043, Galactose oxidase/kelch, beta-propeller	-	Sandaracinaceae bacterium	gb|MBX7193266.1|	hypothetical protein	1368	85	389	268	617	354	83.29	40.395	53.11	187	8.09E-48
AW1 02964	no sign. hit	-	-														
AW1_03167	cd03033 Arsenate Reductase (ArsC) family, 15kD protein subfamily	IPR006660, Arsenate reductase-like	Arsenate reductase (glutaredoxin) activity	Nitrospinae bacterium	gb|MBC8288573.1|	hypothetical protein	132	1	132	1	132	132	100.00	66.667	80.3	194	2.58E-61
AU1-5 00075	no sign. hit	-	-														

[%]Database NCBI-nr [Jan 2022] contained protein sequences for cable bacterium genomes Ca.Electrothrix aarhusiensis MCF, Ca.Electrothrix communis A1, Ca.Electrothrix marina A2, A3, A5 and Ca.Electronema aureum GS. Hits to these cable bacteria genomes were omitted.

*Partial hit. Gene was not predicted with Prokka v1.14.6, but was predicted with GeneMarkS+.

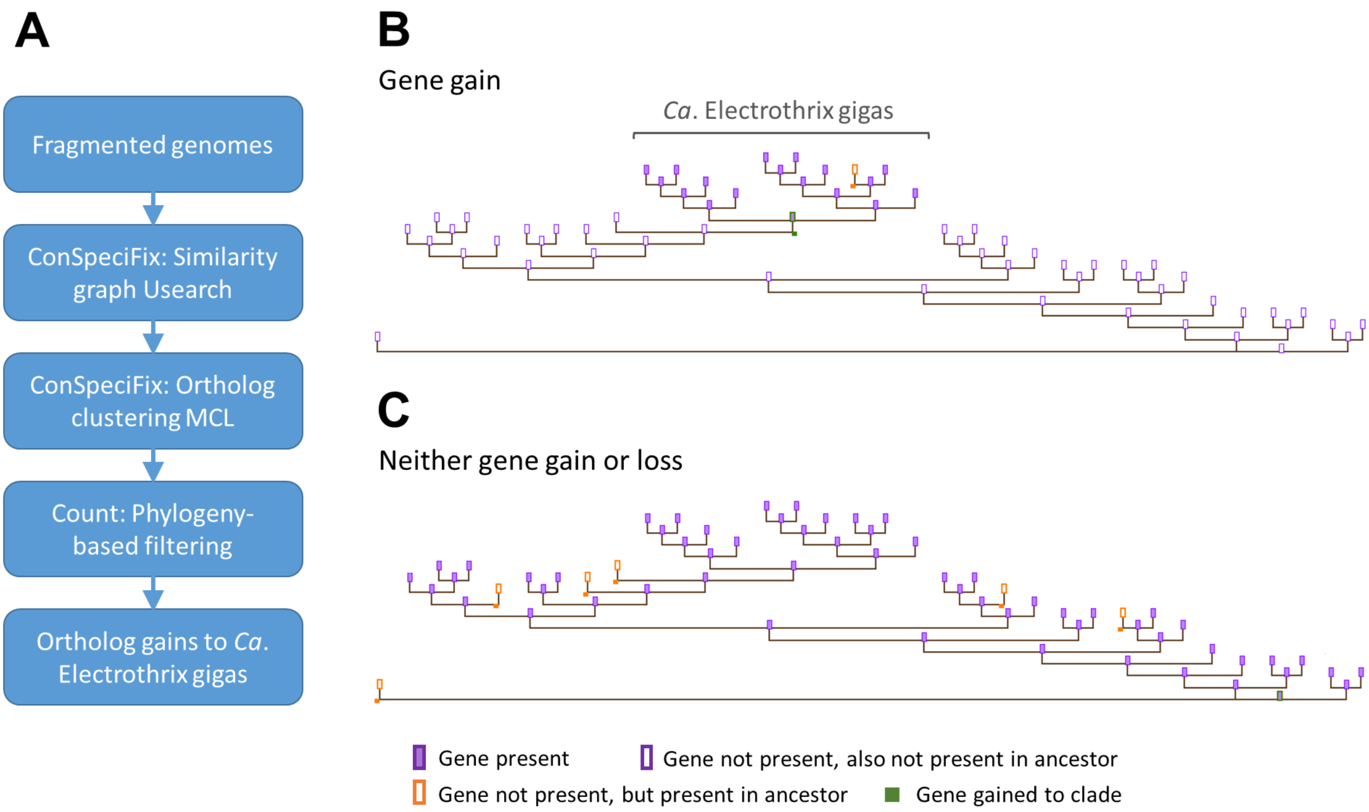

Fig. S1 Method employed for phylogeny-based analysis of gene gain/gene loss in incomplete, fragmented genomes. **(A)** Pipeline consisting of ortholog calling and clustering using ConSpeciFix followed by phylogeny-based analysis of the propensity for gene gain/gene loss using Count **(B)** Example of a gene unique to *Ca*. Electrothrix gigas and **(C)** example of an ortholog that is not present in some genomes, but present in the ancestor suggesting that gene absence is due to genome incompleness.

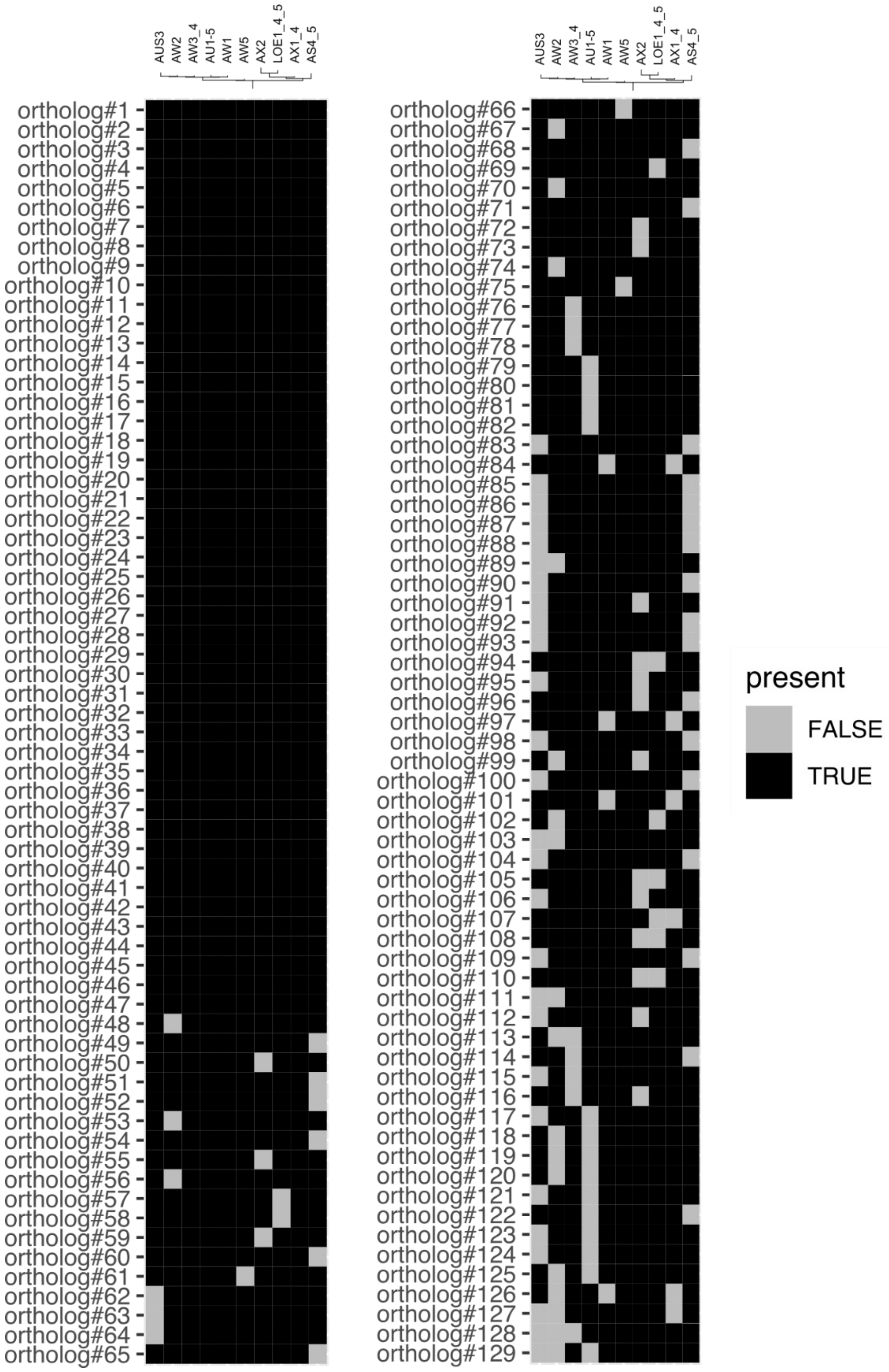

Fig. S2 Presence/absence plot of gene gains in *Ca*. Electrothrix gigas genomes

A

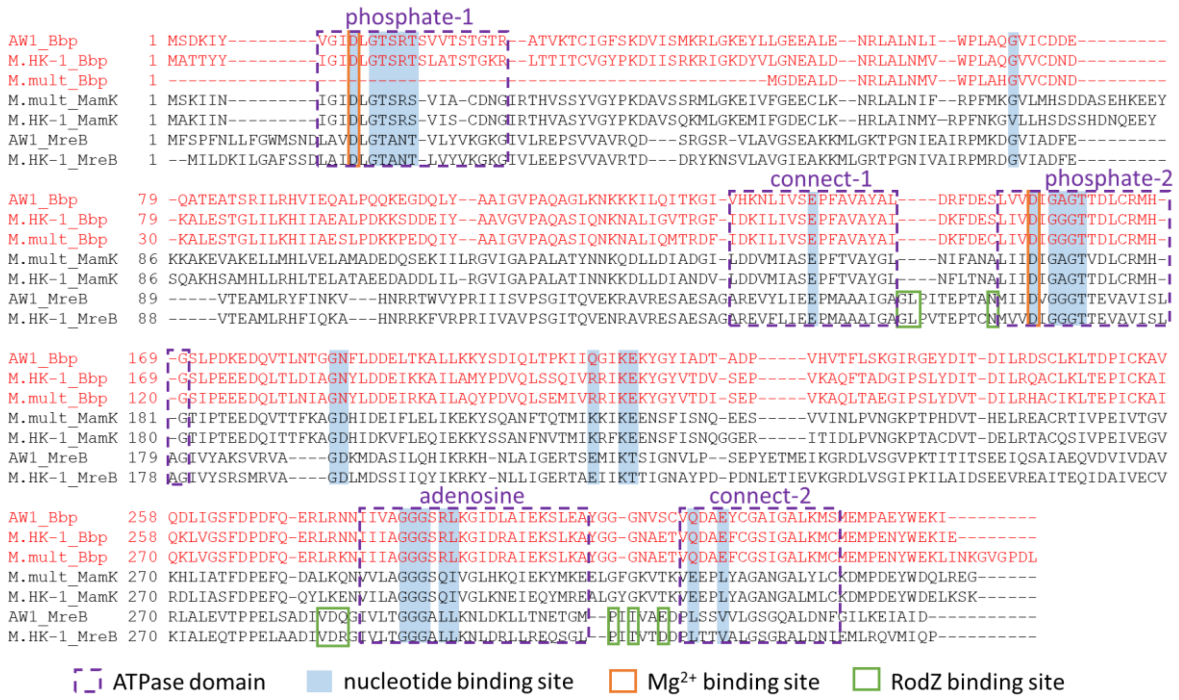

B

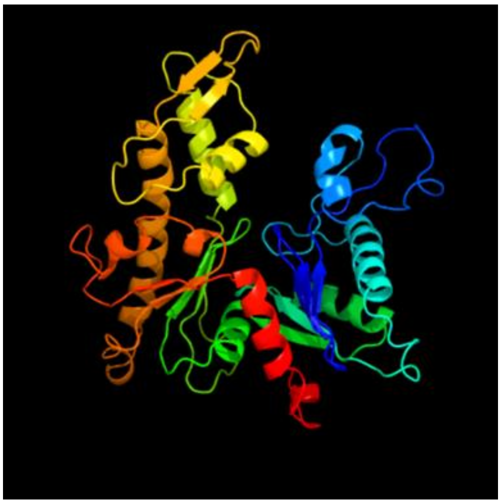

Fig. S3 **(A)** Multiple alignment of sequences of Bbp, MamK and MreB showing the five parts of the ATPase domain and nucleotide and Mg^{2+} binding sites. MreB interacts with the transmembrane protein RodZ. RodZ interacting sites are missing in Bbp. AW1: *Ca.* Electrothrix gigas AW1, M.HK-1: *Ca.* Magnetomorum sp. HK-1, M.mult: *Ca.* Magnetoglobus multicellularis strain Araruama **(B)** Predicted 3D structure of Bbp with Phyre2 using homology detection of experimentally determined 3D protein structures (Kelley et al. 2015 Nat. Protocols 10, 845-858). 98% of the sequence was modelled with 100% confidence to MamK of *Magnetospirillum magneticum* AMB-1 (PDB 5JYG).

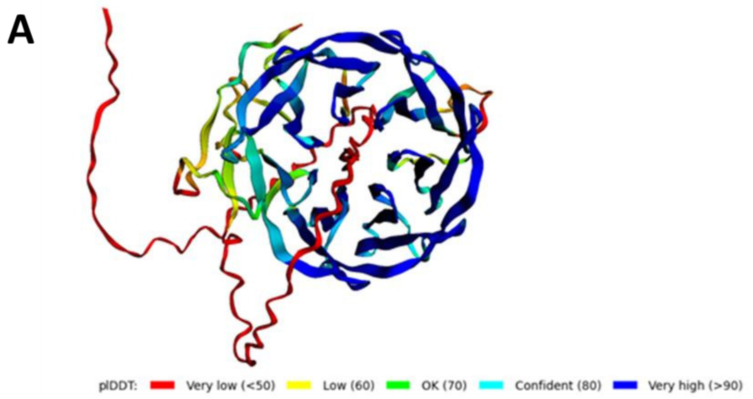

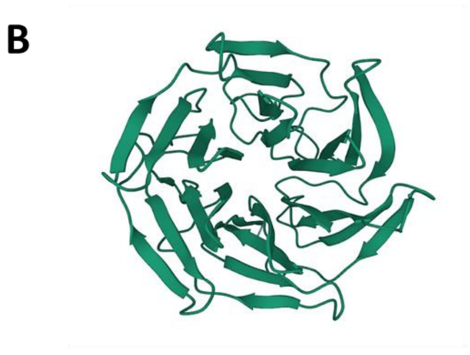

C

```
102-140   ............ISASGNTVAIdshelST....NQFD...V......VYIMTRDDTfCWHPQAK
141-181   LSI.PKINFFDSVSLSSDTLAV.....GA....A..D..........VYIFVRSGT.SWSQQAK
182-235   LSI.PDADWLDSVSLSGDTLAV.....STrydgSSDD...SpvdatvVHIFVRSGT.SWSQQAK
236-279   LSI.PDTSLLDGVSLSGNTLAV.....GT....SSRD...A......VHIFVRSGT.SWSQQAK
280-323   LSI.PDTTSLDSISLSGNTLAV.....GT....SSRD...A......VHIFVRSGS.SWSQQAK
324-369   LNIpPGTDRLDSVSLSGNTLAV.....SA....RYNDdanV......VHIFVRSGT.SWSQQAK
          LsI P    lDsvSlSgnTlAv              D          V IfvRsgT sWsqQAK
```

D

RMSD	TM-score	Sequence Identity	Equivalent Residues	Reference Coverage	Target Coverage
3.65	0.45	5%	232	81%	55%

Fig. S4 (A) Predicted 3D structure of protein AW1_02660 showing a Kelch-like domain. This protein is encoded in a gene cluster with *bbp* (AW1_02658) in *Ca*. Electrothrix gigas. Colours display per-residue confidence of the structure predicted with AlphaFold2. 3D structure was predicted with AlphaFold2 (Jumper et al. 2021 Nature 596, 583–589) using the ColabFold platform (Mirdita et al. 2022 Nat. Methods 19, 679–682) **(B)** 3D protein structure of the Kelch protein KEAP1 (human) (1U6D; Li et al. 2004 J. Biol. Chem. 279, 54750-54758) **(C)** Six repeat motifs of the Kelch domain of *Ca*. Electrothrix gigas protein AW1_02660 with conserved residues indicated. Sequence repeats were identified using the RADAR tool (Maderia et al. 2022 Nuc. Ac. Res. 50, W276-W279; https://www.ebi.ac.uk/Tools/pfa/radar/) **(D)** Structural alignment of the predicted structure of AW1_02660 to KEAP1 (reference) shows low sequence identity but well-aligned structures. 3D structural comparison was performed using the Pairwise Structure Alignment tool of the Research Collaboratory for Structural Bioinformatics (https://www.rcsb.org/alignment).

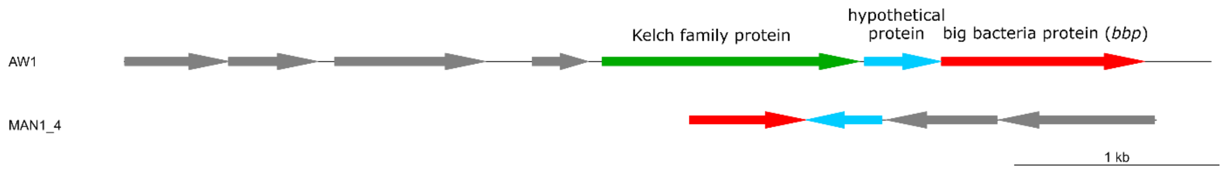

Fig. S5 Gene clusters containing the gene encoding the actin-like big bacteria protein (Bbp) in *Ca*. Electrothrix gigas AW1 and in Ca. Electrothrix MAN1_4 (partial *bbp* gene at the end of a contig). The Kelch family protein was not found in the MAN1_4 genome.

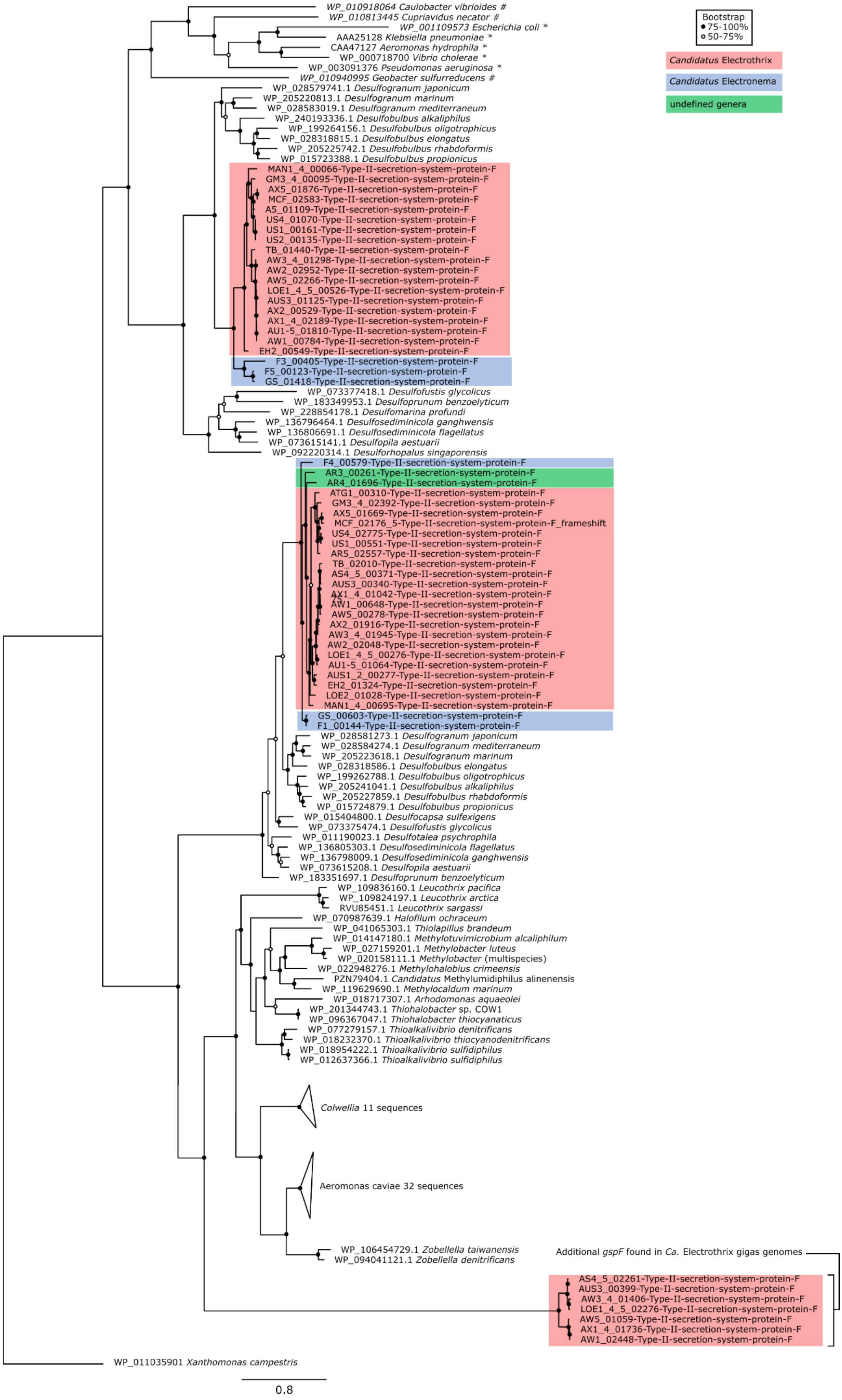

Bootstrap
75-100%
50-75%
Candidatus Electrothrix
Candidatus Electronema
undefined genera
WP_010918064 Caulobacter vibrioides #
WP_010813445 Cupriavidus necator #
WP_001109573 Escherichia coli *
AAA25128 Klebsiella pneumoniae *
CAA47127 Aeromonas hydrophila *
WP_000718700 Vibrio cholerae *
WP_003091376 Pseudomonas aeruginosa *
WP_010940995 Geobacter sulfurreducens #
WP_028579741.1 Desulfogranum japonicum
WP_205220813.1 Desulfogranum marinum
WP_028583019.1 Desulfogranum mediterraneum
WP_240193336.1 Desulfobulbus alkaliphilus
WP_199264156.1 Desulfobulbus oligotrophicus
WP_028318815.1 Desulfobulbus elongatus
WP_205225742.1 Desulfobulbus rhabdoformis
WP_015723388.1 Desulfobulbus propionicus
MAN1_4_00066-Type-II-secretion-system-protein-F
GM3_4_00095-Type-II-secretion-system-protein-F
AX5_01876-Type-II-secretion-system-protein-F
MCF_02583-Type-II-secretion-system-protein-F
A5_01109-Type-II-secretion-system-protein-F
US4_01070-Type-II-secretion-system-protein-F
US1_00161-Type-II-secretion-system-protein-F
US2_00135-Type-II-secretion-system-protein-F
TB_01440-Type-II-secretion-system-protein-F
AW3_4_01298-Type-II-secretion-system-protein-F
AW2_02952-Type-II-secretion-system-protein-F
AW5_02266-Type-II-secretion-system-protein-F
LOE1_4_5_00526-Type-II-secretion-system-protein-F
AUS3_01125-Type-II-secretion-system-protein-F
AX2_00529-Type-II-secretion-system-protein-F
AX1_4_02189-Type-II-secretion-system-protein-F
AU1-5_01810-Type-II-secretion-system-protein-F
AW1_00784-Type-II-secretion-system-protein-F
EH2_00549-Type-II-secretion-system-protein-F
F3_00405-Type-II-secretion-system-protein-F
F5_00123-Type-II-secretion-system-protein-F
GS_01418-Type-II-secretion-system-protein-F
WP_073377418.1 Desulfofustis glycolicus
WP_183349953.1 Desulfoprunum benzoelyticum
WP_228854178.1 Desulfomarina profundi
WP_136796464.1 Desulfosediminicola ganghwensis
WP_136806691.1 Desulfosediminicola flagellatus
WP_073615141.1 Desulfopila aestuarii
WP_092220314.1 Desulforhopalus singaporensis
F4_00579-Type-II-secretion-system-protein-F
AR3_00261-Type-II-secretion-system-protein-F
AR4_01696-Type-II-secretion-system-protein-F
ATG1_00310-Type-II-secretion-system-protein-F
GM3_4_02392-Type-II-secretion-system-protein-F
AX5_01669-Type-II-secretion-system-protein-F
MCF_02176_5-Type-II-secretion-system-protein-F_frameshift
US4_02775-Type-II-secretion-system-protein-F
US1_00551-Type-II-secretion-system-protein-F
AR5_02557-Type-II-secretion-system-protein-F
TB_02010-Type-II-secretion-system-protein-F
AS4_5_00371-Type-II-secretion-system-protein-F
AUS3_00340-Type-II-secretion-system-protein-F
AX1_4_01042-Type-II-secretion-system-protein-F
AW1_00648-Type-II-secretion-system-protein-F
AW5_00278-Type-II-secretion-system-protein-F
AX2_01916-Type-II-secretion-system-protein-F
AW3_4_01945-Type-II-secretion-system-protein-F
AW2_02048-Type-II-secretion-system-protein-F
LOE1_4_5_00276-Type-II-secretion-system-protein-F
AU1-5_01064-Type-II-secretion-system-protein-F
AUS1_2_00277-Type-II-secretion-system-protein-F
EH2_01324-Type-II-secretion-system-protein-F
LOE2_01028-Type-II-secretion-system-protein-F
MAN1_4_00695-Type-II-secretion-system-protein-F
GS_00603-Type-II-secretion-system-protein-F
F1_00144-Type-II-secretion-system-protein-F
WP_028581273.1 Desulfogranum japonicum
WP_028584274.1 Desulfogranum mediterraneum
WP_205223618.1 Desulfogranum marinum
WP_028318586.1 Desulfobulbus elongatus
WP_199262788.1 Desulfobulbus oligotrophicus
WP_205241041.1 Desulfobulbus alkaliphilus
WP_205227859.1 Desulfobulbus rhabdoformis
WP_015724879.1 Desulfobulbus propionicus
WP_015404800.1 Desulfocapsa sulfexigens
WP_073375474.1 Desulfofustis glycolicus
WP_011190023.1 Desulfotalea psychrophila
WP_136805303.1 Desulfosediminicola flagellatus
WP_136798009.1 Desulfosediminicola ganghwensis
WP_073615208.1 Desulfopila aestuarii
WP_183351697.1 Desulfoprunum benzoelyticum
WP_109836160.1 Leucothrix pacifica
WP_109824197.1 Leucothrix arctica
RVU85451.1 Leucothrix sargassi
WP_070987639.1 Halofilum ochraceum
WP_041065303.1 Thiolapillus brandeum
WP_014147180.1 Methylotuvimicrobium alcaliphilum
WP_027159201.1 Methylobacter luteus
WP_020158111.1 Methylobacter (multispecies)
WP_022948276.1 Methylohalobius crimeensis
PZN79404.1 Candidatus Methylumidiphilus alinenensis
WP_119629690.1 Methylocaldum marinum
WP_018717307.1 Arhodomonas aquaeolei
WP_201344743.1 Thiohalobacter sp. COW1
WP_096367047.1 Thiohalobacter thiocyanaticus
WP_077279157.1 Thioalkalivibrio denitrificans
WP_018232370.1 Thioalkalivibrio thiocyanodenitrificans
WP_018954222.1 Thioalkalivibrio sulfidiphilus
WP_012637366.1 Thioalkalivibrio sulfidiphilus
Colwellia 11 sequences
Aeromonas caviae 32 sequences
WP_106454729.1 Zobellella taiwanensis
WP_094041121.1 Zobellella denitrificans
Additional gspF found in Ca. Electrothrix gigas genomes
AS4_5_02261-Type-II-secretion-system-protein-F
AUS3_00399-Type-II-secretion-system-protein-F
AW3_4_01406-Type-II-secretion-system-protein-F
LOE1_4_5_02276-Type-II-secretion-system-protein-F
AW5_01059-Type-II-secretion-system-protein-F
AX1_4_01736-Type-II-secretion-system-protein-F
AW1_02448-Type-II-secretion-system-protein-F
WP_011035901 Xanthomonas campestris
0.8

Fig. S6 Phylogenetic tree of type II secretion platform protein (GspF) amino acid sequences. Cable bacteria genomes encode GspF proteins that are closely related to GspF from other Desulfobulbaceae. *Ca*. Electrothrix gigas genomes encode an additional GspF protein that is affiliated to Gammaproteobacteria. Circles at nodes depict bootstrap support; the scale bar shows mean substitutions per site. Amino acid sequences for the phylogenetic tree of GspF were retrieved from the UniProt database-Reviewed (indicated with *), together with sequences from bacteria from other phyla that encode a functional type II secretion system (indicated with # (White and Cianciotto, 2019 Microbial Genomics 5)). Blastp hits to cable bacteria GspF sequences of cultured species were also included in the analysis. The sequences were aligned with muscle 3.8.31 (Edgar, 2004 Nuc. Ac. Res. 32, 1792-1797) and a tree was constructed with IQ-TREE v. 1.6.12 (Nguyen et al., 2015 Mol. Biol. Evol. 32, 268-274) with best-fit model LG+F+R6 and 1000 bootstraps (option -bb).

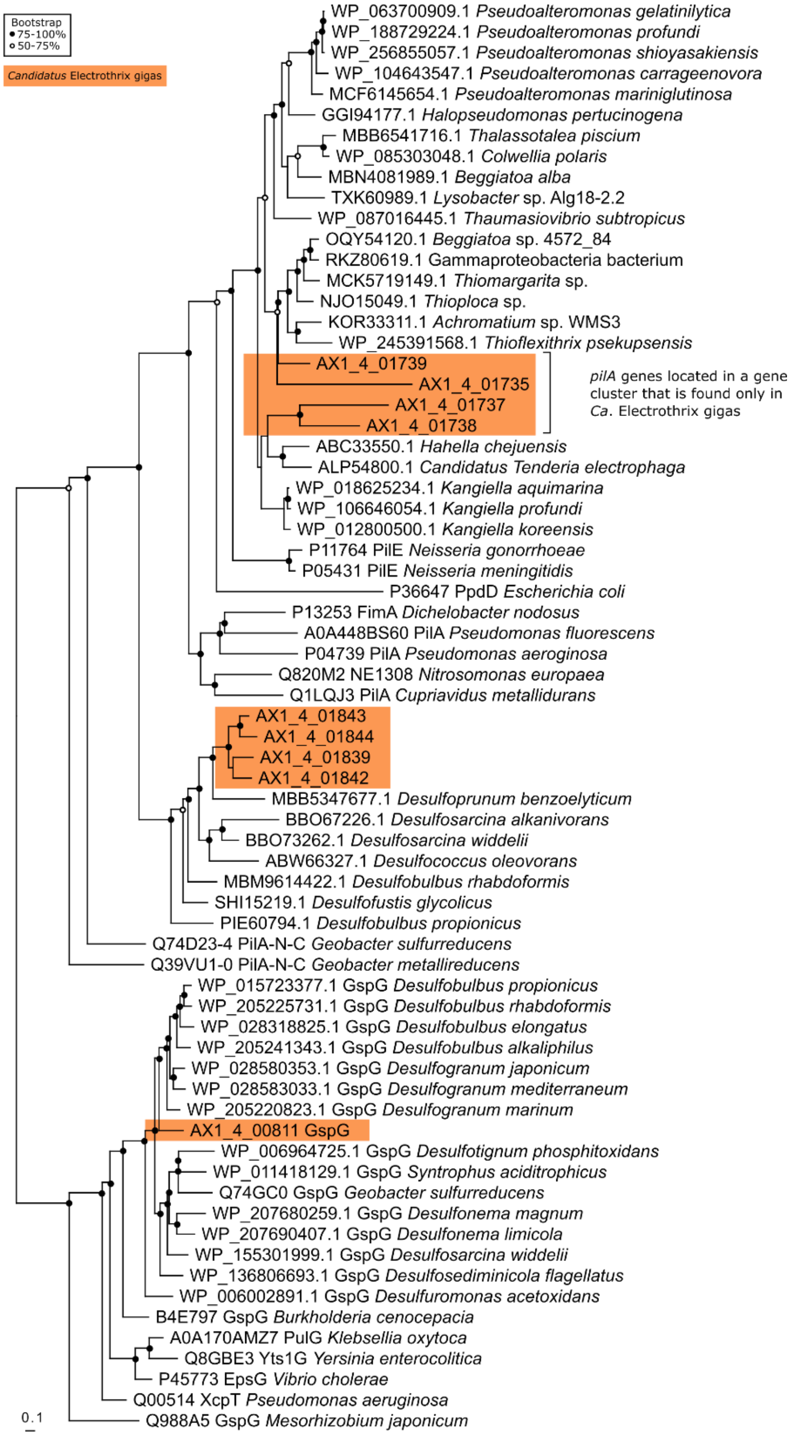

Fig. S7 Phylogenetic tree of type IV pilin (upper part) and pseudopilin (lower part of the tree) amino acid sequences. Circles at nodes depict bootstrap support; the scale bar shows mean substitutions per site. Amino acid sequences for the phylogenetic tree of type IV pilin and type II secretion pseudopilin were retrieved from the UniProt database. For *G. sulfurreducens* and *G. metallireducens*, PilA-N and PilA-C sequences were fused together. Blastp hits to *Ca.* Electrothrix gigas PilA and GspG sequences were included. Sequences were aligned with muscle 3.8.31 (Edgar, 2004 Nuc. Ac. Res. 32, 1792-1797) and a tree was constructed with IQ-TREE v. 1.6.12 (Nguyen et al., 2015 Mol. Biol. Evol. 32, 268-274) with best-fit model WAG+R4 and 1000 bootstraps (option -bb).

Movie S1

A live preparation was made of sediment from Hou, Denmark with gliding *Ca*. Electrothrix gigas. The movie was captured with an iPhone11 in real time and 29.97 frames per second through the eyepiece of a Leica BioMed light microscope, with 40× objective and phase contrast.
The Movie is available as a separate file.
